# Supplementary material for: Calibration adjustments to address bias in mortality analyses due to informative sampling—a census-linked survey analysis in Switzerland
Source: PeerJ. 2018 Feb 13;6:e4376. doi: 10.7717/peerj.4376 (PMC5815334; doi:10.7717/peerj.4376)
Supplement: Table S1 — * IP weights were derived from a logistic model with outcome variable of being in the structural enquiry 2010 and covariates as described in the Methods section. [file peerj-06-4376-s001.docx]

|  |  | CS weighted | IP weighted | |
| --- | --- | --- | --- | --- |
| *STATPOP characteristic* | |  |  |  |
| Age categories* | [15, 20) | 6.8% | 6.8% | |
|  | [20, 25) | 7.3% | 7.5% | |
|  | [25, 30) | 7.8% | 8.0% | |
|  | [30, 35) | 8.0% | 8.0% | |
|  | [35, 40) | 8.4% | 8.4% | |
|  | [40, 45) | 9.6% | 9.5% | |
|  | [45, 50) | 9.8% | 9.7% | |
|  | [50, 55) | 8.6% | 8.4% | |
|  | [55, 60) | 7.4% | 7.2% | |
|  | [60, 65) | 7.0% | 6.8% | |
|  | [65, 70) | 6.0% | 5.9% | |
|  | [70, 75) | 4.6% | 4.5% | |
|  | [75, 80) | 3.8% | 3.8% | |
|  | [80, 85) | 2.8% | 2.9% | |
|  | >=85 | 2.2% | 2.6% | |
| Gender | Men | 49.1% | 49.1% | |
|  | Women | 50.9% | 50.9% | |
| Nationality | Swiss | 78.2% | 77.4% | |
|  | EEA | 14.1% | 14.7% | |
|  | Other Europe | 4.8% | 4.7% | |
|  | Other World | 2.9% | 3.2% | |
| Civil status | Single | 32.6% | 33.2% | |
|  | Married | 52.6% | 51.7% | |
|  | Widowed | 5.7% | 6.0% | |
|  | Other | 9.1% | 9.1% | |
| *Structural enquiry characteristics* | |  |  |  |
| Education | Compulsory or less | 27.2% | 27.4% | |
|  | Upper secondary | 47.9% | 47.7% | |
|  | Tertiary | 24.9% | 24.9% | |
| Employment status | Full-time employed | 44.4% | 44.6% | |
|  | Part-time employed | 18.7% | 18.3% | |
|  | Unemployed | 3.0% | 3.1% | |
|  | Inactive person | 33.9% | 34.0% | |
| Religion | Roman Catholic Church | 38.6% | 38.6% | |
|  | Protestant Churches | 28.0% | 27.9% | |
|  | No religious affiliation | 20.1% | 20.1% | |
|  | Other/No response | 13.3% | 13.4% | |
