# Supplementary material for: Calibration adjustments to address bias in mortality analyses due to informative sampling—a census-linked survey analysis in Switzerland
Source: PeerJ. 2018 Feb 13;6:e4376. doi: 10.7717/peerj.4376 (PMC5815334; doi:10.7717/peerj.4376)
Supplement: Table S2 — Reading example: In the columns for 2010 the deaths by end of 2011, for each month are tabulated. [file peerj-06-4376-s002.docx]

|  | 2010 | | | | 2011 | | | | 2012 | | | | 2013 | | | |
| --- | --- | --- | --- | --- | --- | --- | --- | --- | --- | --- | --- | --- | --- | --- | --- | --- |
|  | STATPOP | | SE | | STATPOP | | SE | | STATPOP | | SE | | STATPOP | | SE | |
| Month | n | % | n | % | n | % | n | % | n | % | n | % | n | % | n | % |
| January | 5,732 | 9.3 | 95 | 4.8 | 5,827 | 9.2 | 67 | 4.2 | 6,073 | 9.4 | 50 | 3.2 | 5,762 | 9.0 | 34 | 2.3 |
| February | 4,994 | 8.1 | 108 | 5.5 | 5,796 | 9.1 | 101 | 6.4 | 5,871 | 9.1 | 104 | 6.6 | 5,351 | 8.4 | 86 | 5.7 |
| March | 5,511 | 9.0 | 166 | 8.4 | 5,855 | 9.2 | 128 | 8.1 | 6,204 | 9.6 | 145 | 9.2 | 5,700 | 8.9 | 121 | 8.0 |
| April | 5,019 | 8.2 | 164 | 8.3 | 5,413 | 8.5 | 132 | 8.3 | 5,421 | 8.4 | 110 | 7.0 | 5,136 | 8.0 | 135 | 9.0 |
| May | 4,975 | 8.1 | 147 | 7.5 | 5,017 | 7.9 | 128 | 8.1 | 5,000 | 7.7 | 135 | 8.6 | 5,204 | 8.1 | 143 | 9.5 |
| June | 4,731 | 7.7 | 162 | 8.2 | 4,799 | 7.5 | 124 | 7.8 | 4,927 | 7.6 | 121 | 7.7 | 4,844 | 7.6 | 104 | 6.9 |
| July | 4,942 | 8.0 | 161 | 8.2 | 4,863 | 7.6 | 141 | 8.9 | 5,160 | 8.0 | 147 | 9.3 | 5,152 | 8.0 | 139 | 9.2 |
| August | 5,032 | 8.2 | 182 | 9.2 | 4,936 | 7.8 | 131 | 8.2 | 5,052 | 7.8 | 146 | 9.3 | 4,981 | 7.8 | 143 | 9.5 |
| September | 4,807 | 7.8 | 191 | 9.7 | 4,914 | 7.7 | 153 | 9.6 | 4,965 | 7.7 | 154 | 9.8 | 4,953 | 7.7 | 123 | 8.2 |
| October | 5,186 | 8.4 | 199 | 10.1 | 5,327 | 8.4 | 153 | 9.6 | 5,215 | 8.1 | 150 | 9.5 | 5,404 | 8.4 | 157 | 10.4 |
| November | 5,087 | 8.3 | 191 | 9.7 | 5,199 | 8.2 | 156 | 9.8 | 5,182 | 8.0 | 160 | 10.2 | 5,582 | 8.7 | 159 | 10.6 |
| December | 5,523 | 9.0 | 205 | 10.4 | 5,658 | 8.9 | 176 | 11.1 | 5,712 | 8.8 | 151 | 9.6 | 5,951 | 9.3 | 162 | 10.8 |
