# Supplementary material for: Calibration adjustments to address bias in mortality analyses due to informative sampling—a census-linked survey analysis in Switzerland
Source: PeerJ. 2018 Feb 13;6:e4376. doi: 10.7717/peerj.4376 (PMC5815334; doi:10.7717/peerj.4376)
Supplement: Table S3 — Abbreviations: STATPOP Registry-based population. [file peerj-06-4376-s003.docx]

|  |  | STATPOP 2011 | STATPOP 2012 | STATPOP 2013 |
| --- | --- | --- | --- | --- |
|  |  | OR (95% CI) | OR (95% CI) | OR (95% CI) |
| Age categories | [15, 20) | Reference | Reference | Reference |
|  | [20, 25) | 0.94 (0.92, 0.96) | 0.92 (0.90, 0.94) | 0.96 (0.93, 0.98) |
|  | [25, 30) | 0.95 (0.93, 0.97) | 0.92 (0.90, 0.94) | 0.97 (0.94, 0.99) |
|  | [30, 35) | 1.00 (0.98, 1.02) | 0.97 (0.95, 1.00) | 1.04 (1.01, 1.06) |
|  | [35, 40) | 1.04 (1.02, 1.07) | 1.02 (0.99, 1.04) | 1.07 (1.04, 1.09) |
|  | [40, 45) | 1.05 (1.03, 1.07) | 1.05 (1.03, 1.08) | 1.08 (1.05, 1.11) |
|  | [45, 50) | 1.06 (1.04, 1.08) | 1.06 (1.04, 1.08) | 1.09 (1.06, 1.12) |
|  | [50, 55) | 1.09 (1.07, 1.11) | 1.06 (1.04, 1.09) | 1.10 (1.07, 1.12) |
|  | [55, 60) | 1.08 (1.06, 1.10) | 1.08 (1.06, 1.11) | 1.11 (1.08, 1.14) |
|  | [60, 65) | 1.11 (1.09, 1.13) | 1.08 (1.05, 1.10) | 1.13 (1.10, 1.16) |
|  | [65, 70) | 1.12 (1.10, 1.14) | 1.11 (1.09, 1.14) | 1.15 (1.11, 1.18) |
|  | [70, 75) | 1.10 (1.07, 1.12) | 1.09 (1.06, 1.12) | 1.13 (1.10, 1.17) |
|  | [75, 80) | 1.10 (1.07, 1.13) | 1.07 (1.04, 1.10) | 1.11 (1.08, 1.15) |
|  | [80, 85) | 0.99 (0.96, 1.01) | 1.00 (0.97, 1.03) | 1.02 (0.99, 1.05) |
|  | >=85 years | 0.77 (0.75, 0.79) | 0.75 (0.73, 0.78) | 0.78 (0.75, 0.81) |
| Gender | Men | Reference | Reference | Reference |
|  | Women | 1.03 (1.02, 1.04) | 1.04 (1.03, 1.05) | 1.03 (1.02, 1.04) |
| Nationality | Swiss | Reference | Reference | Reference |
|  | EEA | 0.91 (0.90, 0.92) | 0.91 (0.90, 0.92) | 0.92 (0.91, 0.93) |
|  | Other Europe | 0.82 (0.81, 0.84) | 0.83 (0.81, 0.84) | 0.81 (0.80, 0.83) |
|  | Other World | 0.77 (0.75, 0.79) | 0.76 (0.74, 0.78) | 0.79 (0.77, 0.81) |
| Civil status | Single | Reference | Reference | Reference |
|  | Married | 1.14 (1.14, 1.15) | 1.15 (1.14, 1.16) | 1.14 (1.13, 1.15) |
|  | Widowed | 1.01 (0.99, 1.03) | 1.00 (0.98, 1.01) | 1.00 (0.98, 1.02) |
|  | Other | 1.07 (1.05, 1.08) | 1.06 (1.04, 1.07) | 1.06 (1.05, 1.08) |
| Vital status by end of year | Alive | Reference | Reference | Reference |
|  | Death | 0.59 (0.56, 0.62) | 0.57 (0.54, 0.60) | 0.57 (0.54, 0.61) |
