# Supplementary figures and images for: Calibration adjustments to address bias in mortality analyses due to informative sampling—a census-linked survey analysis in Switzerland
Source: PeerJ. 2018 Feb 13;6:e4376. doi: 10.7717/peerj.4376 (PMC5815334; doi:10.7717/peerj.4376)

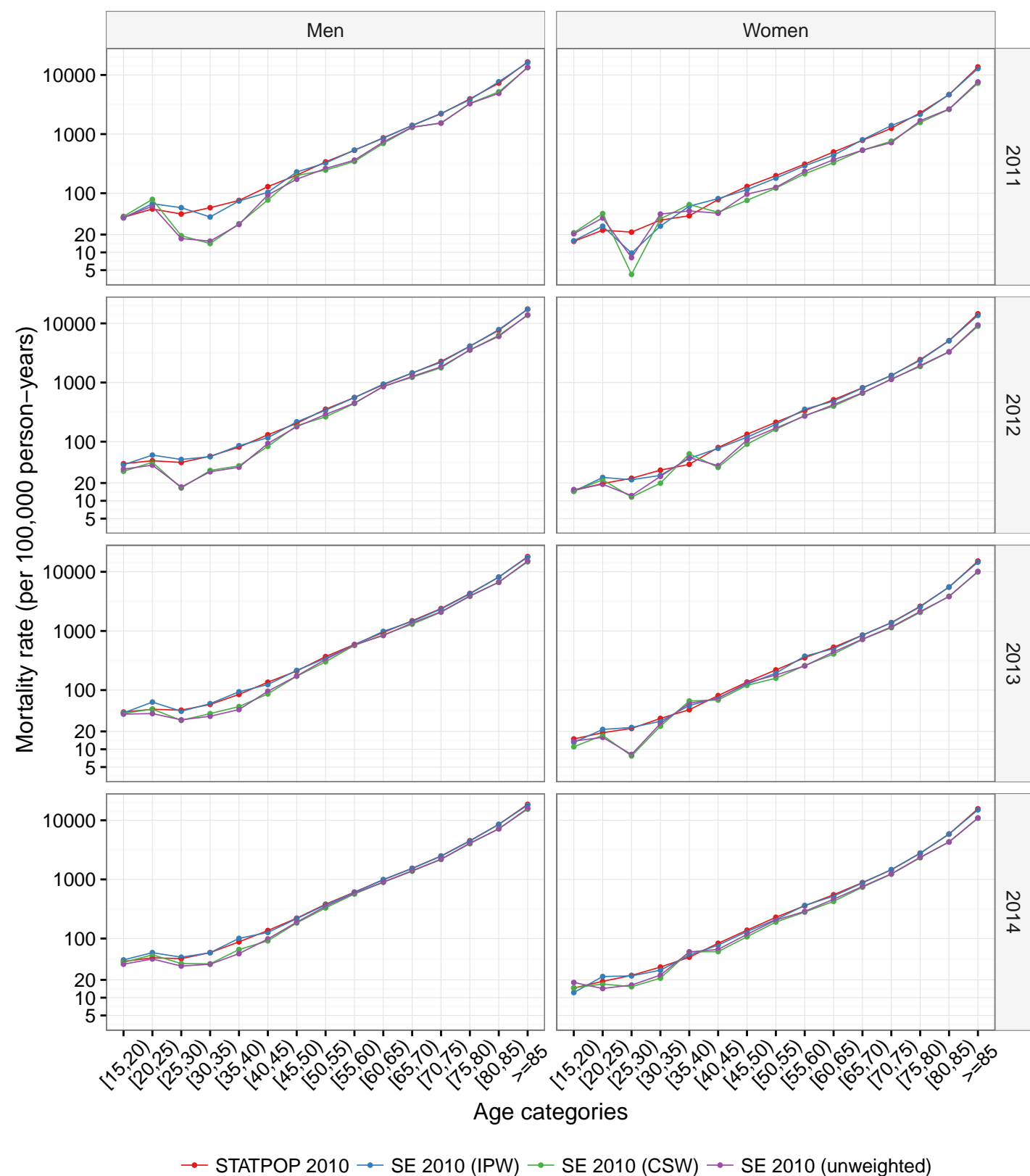

Supplement: Figure S1 — Abbreviations: CSW, Calibrated survey weights; IPW, Inverse probability weights; SE, Structural enquiry; STATPOP Registry-based population. * Permanent residents living in Switzerland aged 15 years or older at December 31, 2010. Y-axis is on a logarithmic scale. [file peerj-06-4376-s004.pdf]

### 10% deaths

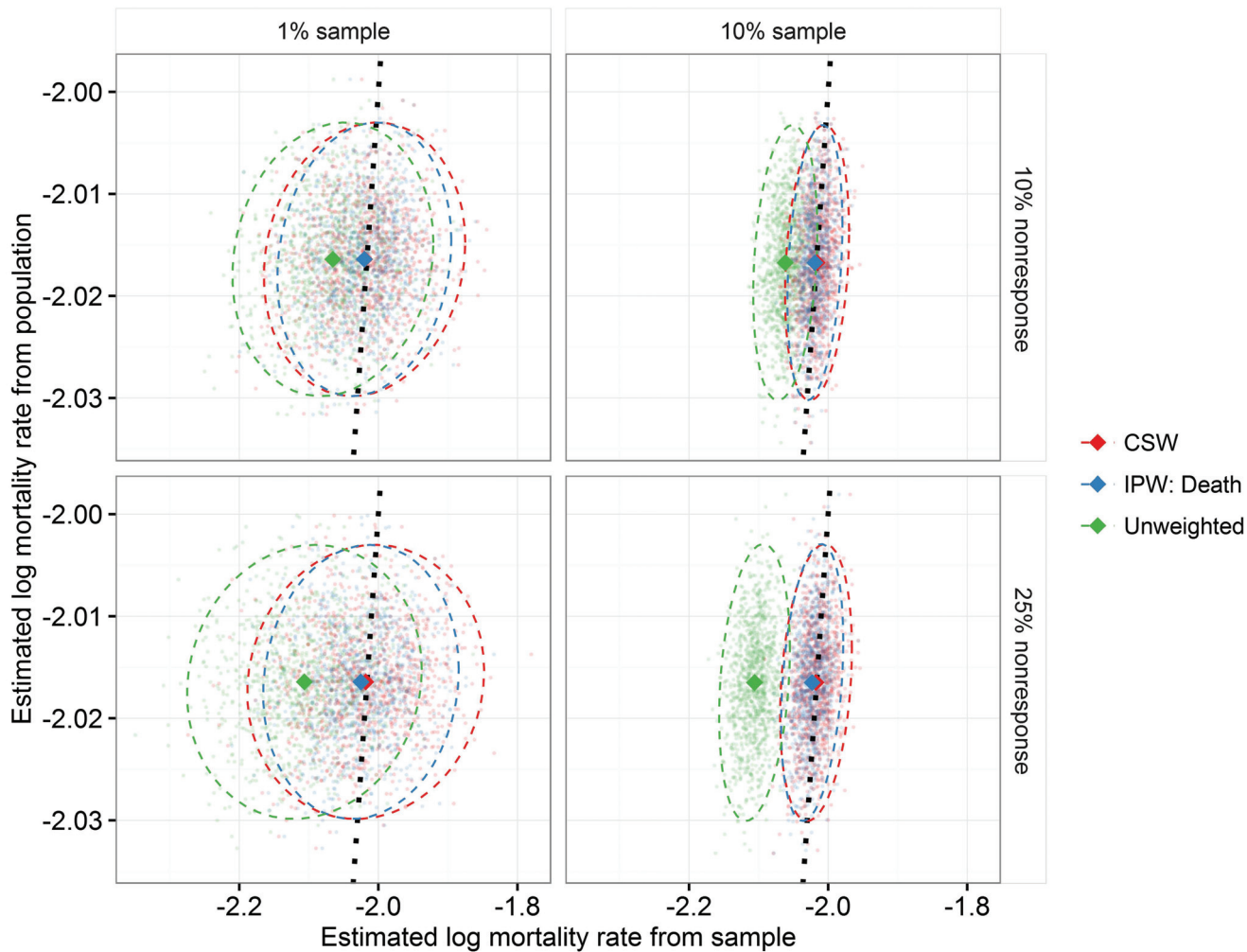

### 25% deaths

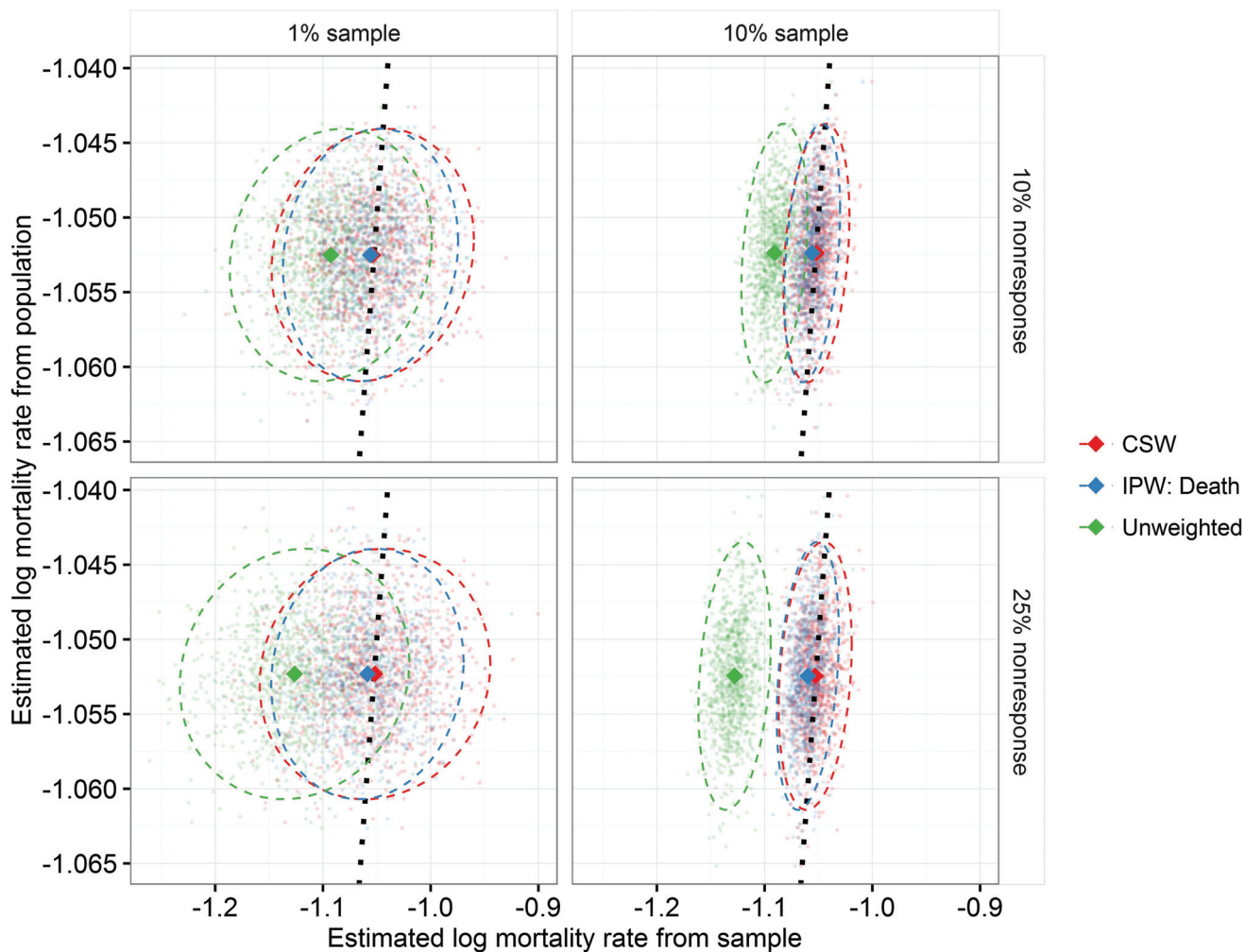

Supplement: Figure S2 — Abbreviations: CSW, Calibrated survey weights; IPW, Inverse probability weights using survival information. * Coloured solid lines from generalized additive model, modelling log mortality rate of population against log mortality rate of sample. Dotted black line indicates a line through the origin with slope one. [file peerj-06-4376-s005.pdf]

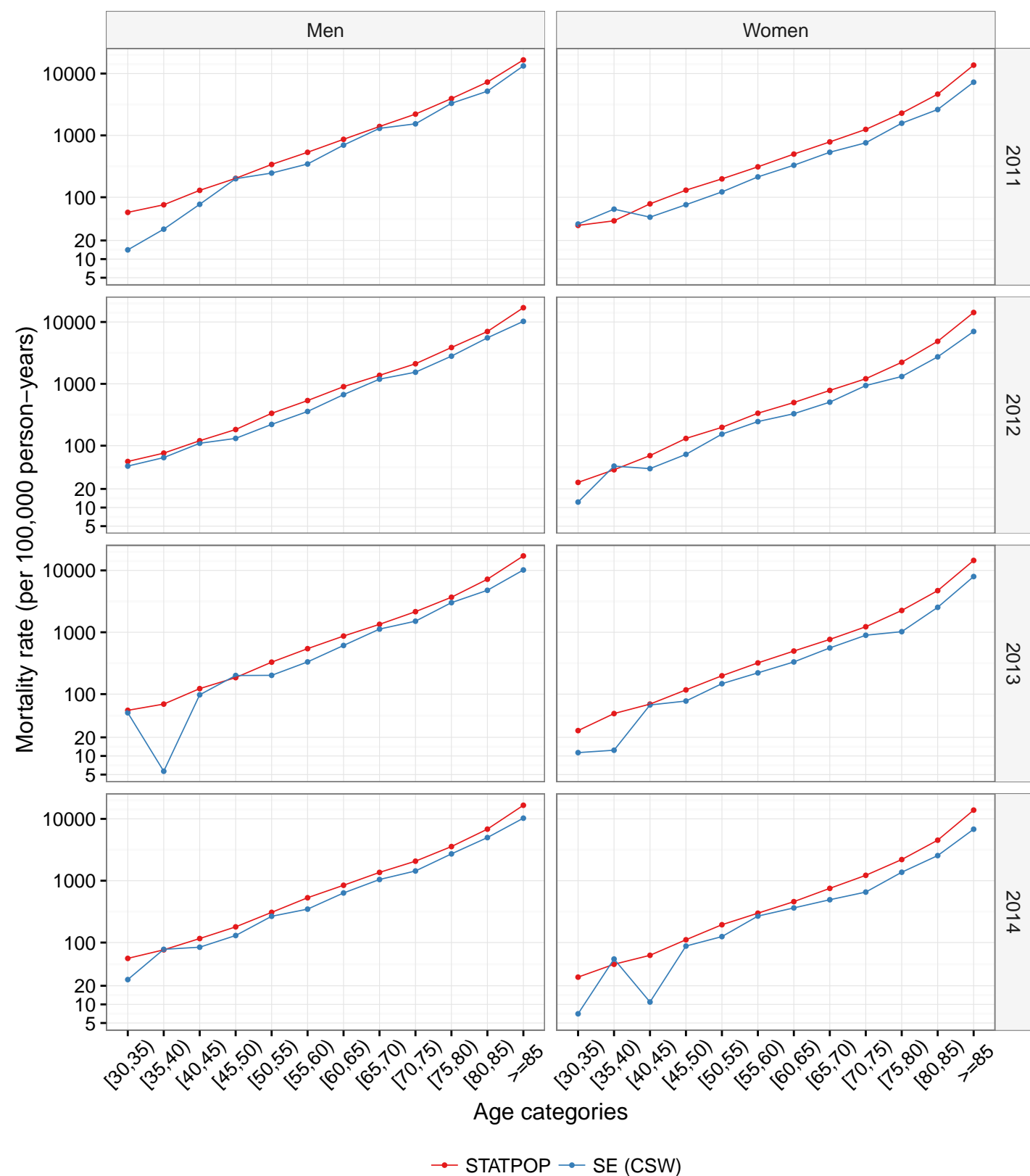

Supplement: Figure S3 — Abbreviations: CSW, Calibrated survey weights; SE, Structural enquiry; STATPOP Registry-based population. * Permanent residents living in Switzerland aged 30 years or older at December 31 of the corresponding sampling year. Y-axis is on a logarithmic scale. [file peerj-06-4376-s006.pdf]
