# Supplementary material for: Calibration adjustments to address bias in mortality analyses due to informative sampling—a census-linked survey analysis in Switzerland
Source: PeerJ. 2018 Feb 13;6:e4376. doi: 10.7717/peerj.4376 (PMC5815334; doi:10.7717/peerj.4376)
Supplement: Supplemental Information 1 — Statistical and analysis for main results, using anonymised dataset (SwissCensus2010.csv). [file peerj-06-4376-s007.pdf]

Calibration adjustments to address bias in mortality analyses due to informative sampling - a census-linked survey analysis in Switzerland

André Moser

November 06, 2017

To ensure reproducibility of our main results, we provide an anonymised dataset (comma-seperated file "SwissCensus2010.csv") of the 2010 study population (STATPOP and SE). Because of privacy preservation of study participants we were not allowed to deliver exact age information - as in the original analysis -, but only rounded to one decimal digit. We highlight that rounding had no influence on the interpretation of our results (i.e., mortality rates changed at maximum at the first decimal place), compared to exact information. IP weights were constructed in Stata, as described in a seperate code.

We provide data and analysis code for Tables 1 and 2, and Figure 1 and 2, in the main manuscript. Estimates for life expectancy (Figure 2) are shown as Stata output, using the same dataset (SwissCensus2010.csv).

Variables used:

- female: Gender (0: Male; 1 Female)
- agestart_rounded: Age at December 31, 2010, rounded to one decimal place
- ageend_rounded: Age at December 31, 2011, or death, rounded to one decimal place
- statpop_agecat5: 15 age categories (0: [15,20); 1: [20,25); ...), at December 31, 2010
- civil: Civil status (1: Single; 2: Married; 3: Widowed; 4: Other)
- nat: Nationality (1: Swiss; 2: EEA; 3: Other Europe; 4: Other World)
- canton: 29 regions
- died2011: Individual died between December 31, 2010, and December 31, 2011
- inse2010: Binary dndicator for "being sampled"" in structural enquiry (SE) 2010 (0: Not sampled; 1: Sampled)
- fupt_rounded: Follow-up time (in years), rounded to two decimal places
- weight2010: Calibrated survey weights (CSW)
- ipw2010: Inverse probability weights (IPW)

```
library(reporttools)
library(rms)
library(ggplot2)

data <- read.csv( paste(path, "SwissCensus2010.csv", sep=""))
# Ordering of columns
data <- data[, c("female", "agestart_rounded", "ageend_rounded",
                 "statpop_agecat5", "civil", "nat", "canton",
                 "died2011", "inse2010", "fupt_rounded",
                 "weight2010", "ipw2010")]
```

The dataset contains 12 variables,

```
head(data[data$died2011==1 & data$inse2010==1, ], 3)
```

```
##        female agestart_rounded ageend_rounded statpop_agecat5 civil nat
## 1896        0             79.3           79.8              12     2   1
## 10821       1             96.4           97.1              14     3   1
## 12955       0             47.1           47.5               6     4   2
##        canton died2011 inse2010 fupt_rounded weight2010  ipw2010
## 1896       23        1        1          0.5   32.89608 41.50185
## 10821      12        1        1          0.7   28.83645 62.33053
## 12955      21        1        1          0.4   18.05437 23.09814
```

with a total of 6,729,363 individuals (STATPOP 2010),

```
nrow(data)
```

```
## [1] 6729363
```

and 317,079 individuals sampled in the structural enquiry (SE2010).

```
nrow(data[data$inse2010==1, ])
```

```
## [1] 317079
```

Table 1

```
tableNominal(vars=data[,c("female", "statpop_agecat5", "civil", "nat",
                          "canton")], group=data$died2011, cumsum=F,
                          cap="STATPOP 2010: Categorical variables")
```

Variable	Levels	n_0	$\%_0$	n_1	$\%_1$	n_{all}	$\%_{all}$
female	0	3270394	49.0	29804	48.4	3300198	49.0
	1	3397430	51.0	31735	51.6	3429165	51.0
	all	6667824	100.0	61539	100.0	6729363	100.0
statpop_agecat5	0	453418	6.8	125	0.2	453543	6.7
	1	497932	7.5	195	0.3	498127	7.4
	2	535327	8.0	179	0.3	535506	8.0
	3	542177	8.1	250	0.4	542427	8.1
	4	562531	8.4	330	0.5	562861	8.4
	5	636715	9.6	662	1.1	637377	9.5
	6	651880	9.8	1087	1.8	652967	9.7
	7	565305	8.5	1525	2.5	566830	8.4
	8	484779	7.3	2048	3.3	486827	7.2
	9	456872	6.8	3110	5.0	459982	6.8
	10	393251	5.9	4259	6.9	397510	5.9
	11	298540	4.5	5082	8.3	303622	4.5
	12	248981	3.7	7565	12.3	256546	3.8
	13	184460	2.8	10711	17.4	195171	2.9
	14	155656	2.3	24411	39.7	180067	2.7
	all	6667824	100.0	61539	100.0	6729363	100.0
civil	1	2224776	33.4	7455	12.1	2232231	33.2
	2	3449816	51.7	24937	40.5	3474753	51.6

	3	387352	5.8	23109	37.5	410461	6.1
	4	605880	9.1	6038	9.8	611918	9.1
	all	6667824	100.0	61539	100.0	6729363	100.0
nat	1	5147117	77.2	56418	91.7	5203535	77.3
	2	989417	14.8	4309	7.0	993726	14.8
	3	317233	4.8	538	0.9	317771	4.7
	4	214057	3.2	274	0.4	214331	3.2
	all	6667824	100.0	61539	100.0	6729363	100.0
canton	1	837660	12.6	6681	10.9	844341	12.6
	2	689868	10.3	7072	11.5	696940	10.4
	3	318109	4.8	2750	4.5	320859	4.8
	4	29375	0.4	315	0.5	29690	0.4
	5	122585	1.8	1053	1.7	123638	1.8
	6	29675	0.4	249	0.4	29924	0.4
	7	34471	0.5	270	0.4	34741	0.5
	8	32785	0.5	364	0.6	33149	0.5
	9	94883	1.4	704	1.1	95587	1.4
	10	228838	3.4	1948	3.2	230786	3.4
	11	216854	3.2	2161	3.5	219015	3.2
	12	162021	2.4	2026	3.3	164047	2.4
	13	234029	3.5	2195	3.6	236224	3.5
	14	65688	1.0	713	1.2	66401	1.0
	15	43940	0.7	444	0.7	44384	0.7
	16	12738	0.2	135	0.2	12873	0.2
	17	405528	6.1	3657	5.9	409185	6.1
	18	174072	2.6	1615	2.6	175687	2.6
	19	513802	7.7	4350	7.1	518152	7.7
	20	209311	3.1	1881	3.1	211192	3.1
	21	284417	4.3	2856	4.6	287273	4.3
	22	603119	9.1	5165	8.4	608284	9.0
	23	265481	4.0	2492	4.0	267973	4.0
	24	143272	2.1	1501	2.4	144773	2.1
	25	365634	5.5	3013	4.9	368647	5.5
	26	57600	0.9	642	1.0	58242	0.9
	27	43374	0.6	518	0.8	43892	0.6
	28	113968	1.7	1331	2.2	115299	1.7
	29	334727	5.0	3438	5.6	338165	5.0
	all	6667824	100.0	61539	100.0	6729363	100.0

Table 1: STATPOP 2010: Categorical variables

```
tableNominal(vars=data[,c("female", "statpop_agecat5", "civil", "nat",
                    "canton", "inse2010")], group=data$died2011,
        data$inse2010==1, cumsum=F, cap="SE 2010: Categorical variables")
```

**Variable**	**Levels**	$\mathbf{n}_0$	$\%_0$	$\mathbf{n}_1$	$\%_1$	$\mathbf{n}_{\text{all}}$	$\%_{\text{all}}$
female	0	151684	48.1	1099	55.8	152783	48.2
	1	163424	51.9	872	44.2	164296	51.8
	all	315108	100.0	1971	100.0	317079	100.0
statpop_agecat5	0	19890	6.3	6	0.3	19896	6.3
	1	20467	6.5	10	0.5	20477	6.5
	2	23935	7.6	3	0.1	23938	7.5
	3	26445	8.4	8	0.4	26453	8.3
	4	27456	8.7	11	0.6	27467	8.7
	5	30282	9.6	21	1.1	30303	9.6
	6	31172	9.9	42	2.1	31214	9.8

	7	26896	8.5	52	2.6	26948	8.5
	8	23522	7.5	70	3.5	23592	7.4
	9	22204	7.0	122	6.2	22326	7.0
	10	19730	6.3	180	9.1	19910	6.3
	11	15000	4.8	163	8.3	15163	4.8
	12	12520	4.0	300	15.2	12820	4.0
	13	9040	2.9	323	16.4	9363	3.0
	14	6549	2.1	660	33.5	7209	2.3
	all	315108	100.0	1971	100.0	317079	100.0
civil	1	101868	32.3	189	9.6	102057	32.2
	2	166452	52.8	999	50.7	167451	52.8
	3	17942	5.7	592	30.0	18534	5.8
	4	28846	9.2	191	9.7	29037	9.2
	all	315108	100.0	1971	100.0	317079	100.0
nat	1	249184	79.1	1779	90.3	250963	79.2
	2	45200	14.3	172	8.7	45372	14.3
	3	12503	4.0	14	0.7	12517	4.0
	4	8221	2.6	6	0.3	8227	2.6
	all	315108	100.0	1971	100.0	317079	100.0
canton	1	24948	7.9	119	6.0	25067	7.9
	2	21957	7.0	130	6.6	22087	7.0
	3	19208	6.1	96	4.9	19304	6.1
	4	976	0.3	5	0.2	981	0.3
	5	3589	1.1	19	1.0	3608	1.1
	6	828	0.3	4	0.2	832	0.3
	7	1039	0.3	6	0.3	1045	0.3
	8	914	0.3	7	0.4	921	0.3
	9	5205	1.6	23	1.2	5228	1.6
	10	6570	2.1	39	2.0	6609	2.1
	11	6598	2.1	54	2.7	6652	2.1
	12	5113	1.6	26	1.3	5139	1.6
	13	7366	2.3	44	2.2	7410	2.3
	14	2001	0.6	18	0.9	2019	0.6
	15	1382	0.4	15	0.8	1397	0.4
	16	381	0.1	2	0.1	383	0.1
	17	12174	3.9	57	2.9	12231	3.9
	18	5070	1.6	32	1.6	5102	1.6
	19	30046	9.5	164	8.3	30210	9.5
	20	12137	3.8	73	3.7	12210	3.8
	21	17343	5.5	123	6.2	17466	5.5
	22	34465	10.9	232	11.8	34697	10.9
	23	7112	2.3	49	2.5	7161	2.3
	24	9295	3.0	73	3.7	9368	3.0
	25	20510	6.5	112	5.7	20622	6.5
	26	3723	1.2	30	1.5	3753	1.2
	27	2899	0.9	22	1.1	2921	0.9
	28	13139	4.2	111	5.6	13250	4.2
	29	39120	12.4	286	14.5	39406	12.4
	all	315108	100.0	1971	100.0	317079	100.0
inse2010	1	315108	100.0	1971	100.0	317079	100.0
	all	315108	100.0	1971	100.0	317079	100.0

Table 2: SE 2010: Categorical variables

```
tableContinuous(vars=data[,c("weight2010", "ipw2010")], group=data$died2011,
                cap="STATPOP 2010: Continuous variables")
```

Variable	Levels	n	Min	q_1	$\tilde{x}$	$\bar{x}$	q_3	Max	s	IQR	#NA
weight2010	0	315108	3.3	15.0	16.8	20.6	30.3	75.0	9.4	15.3	6352716
	1	1971	6.4	14.6	16.1	19.4	30.1	43.4	9.3	15.5	59568
	all	317079	3.3	15.0	16.8	20.6	30.3	75.0	9.4	15.3	6412284
ipw2010	0	315108	7.3	15.2	17.6	21.2	30.2	61.8	9.6	15.0	6352716
	1	1971	5.8	17.0	26.0	30.2	39.3	113.4	17.3	22.3	59568
	all	317079	5.8	15.2	17.6	21.2	30.2	113.4	9.7	15.0	6412284

Table 3: STATPOP 2010: Continuous variables

```
tableContinuous(vars=data[,c("weight2010", "ipw2010")], group=data$died2011,
                subset=data$inse2010==1, cap="SE 2010: Continuous variables")
```

Variable	Levels	n	Min	q_1	$\tilde{x}$	$\bar{x}$	q_3	Max	s	IQR	#NA
weight2010	0	315108	3.3	15.0	16.8	20.6	30.3	75.0	9.4	15.3	0
	1	1971	6.4	14.6	16.1	19.4	30.1	43.4	9.3	15.5	0
	all	317079	3.3	15.0	16.8	20.6	30.3	75.0	9.4	15.3	0
ipw2010	0	315108	7.3	15.2	17.6	21.2	30.2	61.8	9.6	15.0	0
	1	1971	5.8	17.0	26.0	30.2	39.3	113.4	17.3	22.3	0
	all	317079	5.8	15.2	17.6	21.2	30.2	113.4	9.7	15.0	0

Table 4: SE 2010: Continuous variables

Table 2

```
output <- c()

mod <- glm(inse2010 ~ factor(statpop_agecat5), data=data, family = binomial())

output <- rbind(output,
                data.frame(var=names(mod$coefficients[2:length(mod$coefficients)]),
                           est=exp(mod$coefficients[2:length(mod$coefficients)]),
                           lci=exp(confint.default(mod)[2:length(mod$coefficients),1]),
                           uci=exp(confint.default(mod)[2:length(mod$coefficients),2])))
rm(mod)

mod <- glm(inse2010 ~ female, data=data, family = binomial())

output <- rbind(output,
                data.frame(var=names(mod$coefficients[2:length(mod$coefficients)]),
                           est=exp(mod$coefficients[2:length(mod$coefficients)]),
                           lci=exp(confint.default(mod)[2:length(mod$coefficients),1]),
                           uci=exp(confint.default(mod)[2:length(mod$coefficients),2])))
rm(mod)

mod <- glm(inse2010 ~ factor(nat), data=data, family = binomial())

output <- rbind(output,
```

```r
                data.frame(var=names(mod$coefficients[2:length(mod$coefficients)]),
                           est=exp(mod$coefficients[2:length(mod$coefficients)]),
                           lci=exp(confint.default(mod)[2:length(mod$coefficients),1]),
                           uci=exp(confint.default(mod)[2:length(mod$coefficients),2])))
rm(mod)

mod <- glm(inse2010 ~ factor(civil), data=data, family = binomial())

output <- rbind(output,
                data.frame(var=names(mod$coefficients[2:length(mod$coefficients)]),
                           est=exp(mod$coefficients[2:length(mod$coefficients)]),
                           lci=exp(confint.default(mod)[2:length(mod$coefficients),1]),
                           uci=exp(confint.default(mod)[2:length(mod$coefficients),2])))
rm(mod)

mod <- glm(inse2010 ~ factor(canton), data=data, family = binomial())

output <- rbind(output,
                data.frame(var=names(mod$coefficients[2:length(mod$coefficients)]),
                           est=exp(mod$coefficients[2:length(mod$coefficients)]),
                           lci=exp(confint.default(mod)[2:length(mod$coefficients),1]),
                           uci=exp(confint.default(mod)[2:length(mod$coefficients),2])))
rm(mod)

mod <- glm(inse2010 ~ died2011, data=data, family = binomial())

output <- rbind(output,
                data.frame(var=names(mod$coefficients[2:length(mod$coefficients)]),
                           est=exp(mod$coefficients[2:length(mod$coefficients)]),
                           lci=exp(confint.default(mod)[2:length(mod$coefficients),1]),
                           uci=exp(confint.default(mod)[2:length(mod$coefficients),2])))
rm(mod)

dimnames(output)[[1]] <- 1:length(dimnames(output)[[1]])

output
```

```
##                             var       est       lci       uci
## 1   factor(statpop_agecat5)1 0.9343878 0.9159445 0.9532024
## 2   factor(statpop_agecat5)2 1.0198941 1.0004681 1.0396972
## 3   factor(statpop_agecat5)3 1.1174232 1.0965803 1.1386622
## 4   factor(statpop_agecat5)4 1.1181712 1.0974791 1.1392534
## 5   factor(statpop_agecat5)5 1.0879641 1.0682335 1.1080591
## 6   factor(statpop_agecat5)6 1.0942136 1.0744830 1.1143065
## 7   factor(statpop_agecat5)7 1.0879232 1.0677159 1.1085130
## 8   factor(statpop_agecat5)8 1.1100281 1.0887962 1.1316740
## 9   factor(statpop_agecat5)9 1.1118561 1.0903167 1.1338210
```

```
## 10 factor(statpop_agecat5)10 1.1492376 1.1263416 1.1725991
## 11 factor(statpop_agecat5)11 1.1457018 1.1211694 1.1707710
## 12 factor(statpop_agecat5)12 1.1464542 1.1206745 1.1728270
## 13 factor(statpop_agecat5)13 1.0983005 1.0710174 1.1262786
## 14 factor(statpop_agecat5)14 0.9089838 0.8843204 0.9343351
## 15                     female 1.0366692 1.0292986 1.0440927
## 16              factor(nat)2 0.9441447 0.9345296 0.9538587
## 17              factor(nat)3 0.8092078 0.7945234 0.8241636
## 18              factor(nat)4 0.7877280 0.7702867 0.8055643
## 19            factor(civil)2 1.0567839 1.0483939 1.0652410
## 20            factor(civil)3 0.9870434 0.9713627 1.0029773
## 21            factor(civil)4 1.0397864 1.0259945 1.0537636
## 22           factor(canton)2 1.0696812 1.0502078 1.0895156
## 23           factor(canton)3 2.0922211 2.0523785 2.1328372
## 24           factor(canton)4 1.1168060 1.0466629 1.1916499
## 25           factor(canton)5 0.9824343 0.9482447 1.0178567
## 26           factor(canton)6 0.9347093 0.8714731 1.0025340
## 27           factor(canton)7 1.0135957 0.9518682 1.0793261
## 28           factor(canton)8 0.9340134 0.8737523 0.9984305
## 29           factor(canton)9 1.8909976 1.8340432 1.9497207
## 30          factor(canton)10 0.9635439 0.9374064 0.9904102
## 31          factor(canton)11 1.0237648 0.9960445 1.0522566
## 32          factor(canton)12 1.0569627 1.0252226 1.0896855
## 33          factor(canton)13 1.0584307 1.0309285 1.0866666
## 34          factor(canton)14 1.0249405 0.9788160 1.0732386
## 35          factor(canton)15 1.0621506 1.0055655 1.1219198
## 36          factor(canton)16 1.0022202 0.9046300 1.1103382
## 37          factor(canton)17 1.0070443 0.9851831 1.0293907
## 38          factor(canton)18 0.9775217 0.9481088 1.0078472
## 39          factor(canton)19 2.0235285 1.9891880 2.0584619
## 40          factor(canton)20 2.0055278 1.9615391 2.0505030
## 41          factor(canton)21 2.1157632 2.0742793 2.1580767
## 42          factor(canton)22 1.9770581 1.9445223 2.0101383
## 43          factor(canton)23 0.8973728 0.8737921 0.9215899
## 44          factor(canton)24 2.2612001 2.2066489 2.3170998
## 45          factor(canton)25 1.9366322 1.9004721 1.9734803
## 46          factor(canton)26 2.2511091 2.1728492 2.3321876
## 47          factor(canton)27 2.3301389 2.2397066 2.4242225
## 48          factor(canton)28 4.2435951 4.1511140 4.3381365
## 49          factor(canton)29 4.3109022 4.2408682 4.3820927
## 50                  died2011 0.6670748 0.6377409 0.6977579
```

```
rm(mod)
```

Figure 1

```
output <- c()

### Mortality rates of STATPOP population (type=1), by gender

type <- 1

sex <- 1
mod <- glm(died2011 ~ factor(statpop_agecat5)-1, data=data,
           offset=log(fupt_rounded), subset=female==sex, family=poisson())
output <- rbind(output, data.frame(agecat=as.numeric(mod$xlevels[[1]]),
                               female=sex, rate=exp(mod$coeff), type=type))
sex <- 0
mod <- glm(died2011 ~ factor(statpop_agecat5)-1, data=data,
           offset=log(fupt_rounded), subset=female==sex, family=poisson())
output <- rbind(output, data.frame(agecat=as.numeric(mod$xlevels[[1]]),
                               female=sex, rate=exp(mod$coeff), type=type))


### Restricte to SE 2010 population
data <- data[data$inse2010==1, ]

### Mortality rates of SE 2010 population using IPW weights (type=2), by gender

type <- 2

sex <- 1
mod <- glm(died2011 ~ factor(statpop_agecat5)-1, data=data, offset=log(fupt_rounded),
           subset=female==sex, family=poisson(), weights=ipw2010)
output <- rbind(output, data.frame(agecat=as.numeric(mod$xlevels[[1]]),
                               female=sex, rate=exp(mod$coeff), type=type))
sex <- 0
mod <- glm(died2011 ~ factor(statpop_agecat5)-1, data=data, offset=log(fupt_rounded),
           subset=female==sex, family=poisson(), weights=ipw2010)
output <- rbind(output, data.frame(agecat=as.numeric(mod$xlevels[[1]]),
                               female=sex, rate=exp(mod$coeff), type=type))


### Mortality rates of SE 2010 population using CSW weights (type=3), by gender

type <- 3

sex <- 1
mod <- glm(died2011 ~ factor(statpop_agecat5)-1, data=data, offset=log(fupt_rounded),
           subset=female==sex, family=poisson(), weights=weight2010)
output <- rbind(output, data.frame(agecat=as.numeric(mod$xlevels[[1]]),
```

```
                             female=sex, rate=exp(mod$coeff), type=type))

sex <- 0
mod <- glm(died2011 ~ factor(statpop_agecat5)-1, data=data, offset=log(fupt_rounded),
           subset=female==sex, family=poisson(), weights=weight2010)
output <- rbind(output, data.frame(agecat=as.numeric(mod$xlevels[[1]]),
                             female=sex, rate=exp(mod$coeff), type=type))


### Mortality rates of SE 2010 population using no weights (type=4), by gender

type <- 4

sex <- 1
mod <- glm(died2011 ~ factor(statpop_agecat5)-1, data=data, offset=log(fupt_rounded),
           subset=female==sex, family=poisson())
output <- rbind(output, data.frame(agecat=as.numeric(mod$xlevels[[1]]),
                             female=sex, rate=exp(mod$coeff), type=type))

sex <- 0
mod <- glm(died2011 ~ factor(statpop_agecat5)-1, data=data, offset=log(fupt_rounded),
           subset=female==sex, family=poisson())
output <- rbind(output, data.frame(agecat=as.numeric(mod$xlevels[[1]]),
                             female=sex, rate=exp(mod$coeff), type=type))



output$agecat <- factor(output$agecat, levels=0:14,
                            labels=c("[15,20)", "[20,25)", "[25,30)", "[30,35)",
                                     "[35,40)", "[40,45)", "[45,50)", "[50,55)",
                                     "[55,60)", "[60,65)", "[65,70)", "[70,75)",
                                     "[75,80)", "[80,85)", ">=85" ))
output$female <- factor(output$female, levels=0:1, labels=c("Men", "Women"))
output$type <- factor(output$type, levels=1:4, labels=c("STATPOP 2010",
                             "SE 2010 (IPW)", "SE 2010 (CSW)", "SE 2010 (unweighted)"))

factor <- 100000
output$rate <- output$rate*factor
dimnames(output)[[1]] <- 1:length(dimnames(output)[[1]])
```

```
### Show results
output
```

```
##      agecat female        rate               type
## 1   [15,20)  Women   15.377923       STATPOP 2010
## 2   [20,25)  Women   23.696305       STATPOP 2010
## 3   [25,30)  Women   21.996134       STATPOP 2010
## 4   [30,35)  Women   35.067326       STATPOP 2010
```

```
## 5   [35,40)  Women    41.586601         STATPOP 2010
## 6   [40,45)  Women    78.266849         STATPOP 2010
## 7   [45,50)  Women   130.144776         STATPOP 2010
## 8   [50,55)  Women   198.182110         STATPOP 2010
## 9   [55,60)  Women   310.278384         STATPOP 2010
## 10  [60,65)  Women   498.732925         STATPOP 2010
## 11  [65,70)  Women   784.287264         STATPOP 2010
## 12  [70,75)  Women  1251.000126         STATPOP 2010
## 13  [75,80)  Women  2286.724441         STATPOP 2010
## 14  [80,85)  Women  4644.773608         STATPOP 2010
## 15     >=85  Women 13657.925525         STATPOP 2010
## 16  [15,20)    Men    39.199816         STATPOP 2010
## 17  [20,25)    Men    54.180393         STATPOP 2010
## 18  [25,30)    Men    44.578863         STATPOP 2010
## 19  [30,35)    Men    56.975437         STATPOP 2010
## 20  [35,40)    Men    75.497232         STATPOP 2010
## 21  [40,45)    Men   129.308223         STATPOP 2010
## 22  [45,50)    Men   202.020019         STATPOP 2010
## 23  [50,55)    Men   338.941367         STATPOP 2010
## 24  [55,60)    Men   532.887068         STATPOP 2010
## 25  [60,65)    Men   864.189059         STATPOP 2010
## 26  [65,70)    Men  1392.530477         STATPOP 2010
## 27  [70,75)    Men  2209.014961         STATPOP 2010
## 28  [75,80)    Men  3934.881354         STATPOP 2010
## 29  [80,85)    Men  7271.082613         STATPOP 2010
## 30     >=85    Men 16579.584135         STATPOP 2010
## 31  [15,20)  Women    15.679440        SE 2010 (IPW)
## 32  [20,25)  Women    27.720144        SE 2010 (IPW)
## 33  [25,30)  Women     9.714367        SE 2010 (IPW)
## 34  [30,35)  Women    27.847254        SE 2010 (IPW)
## 35  [35,40)  Women    60.422426        SE 2010 (IPW)
## 36  [40,45)  Women    81.052945        SE 2010 (IPW)
## 37  [45,50)  Women   114.689100        SE 2010 (IPW)
## 38  [50,55)  Women   179.922461        SE 2010 (IPW)
## 39  [55,60)  Women   291.687019        SE 2010 (IPW)
## 40  [60,65)  Women   438.695440        SE 2010 (IPW)
## 41  [65,70)  Women   800.636296        SE 2010 (IPW)
## 42  [70,75)  Women  1393.834663        SE 2010 (IPW)
## 43  [75,80)  Women  2161.236569        SE 2010 (IPW)
## 44  [80,85)  Women  4665.285213        SE 2010 (IPW)
## 45     >=85  Women 12813.540168        SE 2010 (IPW)
## 46  [15,20)    Men    38.200556        SE 2010 (IPW)
## 47  [20,25)    Men    66.468834        SE 2010 (IPW)
## 48  [25,30)    Men    56.942586        SE 2010 (IPW)
## 49  [30,35)    Men    39.686788        SE 2010 (IPW)
## 50  [35,40)    Men    73.910474        SE 2010 (IPW)
## 51  [40,45)    Men   103.654033        SE 2010 (IPW)
## 52  [45,50)    Men   228.540248        SE 2010 (IPW)
```

```
## 53  [50,55)    Men   324.563608        SE 2010 (IPW)
## 54  [55,60)    Men   535.784586        SE 2010 (IPW)
## 55  [60,65)    Men   852.768369        SE 2010 (IPW)
## 56  [65,70)    Men  1405.105336        SE 2010 (IPW)
## 57  [70,75)    Men  2235.427910        SE 2010 (IPW)
## 58  [75,80)    Men  3811.316794        SE 2010 (IPW)
## 59  [80,85)    Men  7671.756630        SE 2010 (IPW)
## 60     >=85    Men 16225.323782        SE 2010 (IPW)
## 61  [15,20)  Women    21.449628        SE 2010 (CSW)
## 62  [20,25)  Women    45.284756        SE 2010 (CSW)
## 63  [25,30)  Women     4.242638        SE 2010 (CSW)
## 64  [30,35)  Women    36.903647        SE 2010 (CSW)
## 65  [35,40)  Women    64.170143        SE 2010 (CSW)
## 66  [40,45)  Women    47.662749        SE 2010 (CSW)
## 67  [45,50)  Women    75.698020        SE 2010 (CSW)
## 68  [50,55)  Women   121.975222        SE 2010 (CSW)
## 69  [55,60)  Women   213.247322        SE 2010 (CSW)
## 70  [60,65)  Women   329.160980        SE 2010 (CSW)
## 71  [65,70)  Women   533.659322        SE 2010 (CSW)
## 72  [70,75)  Women   759.558373        SE 2010 (CSW)
## 73  [75,80)  Women  1573.501336        SE 2010 (CSW)
## 74  [80,85)  Women  2619.861638        SE 2010 (CSW)
## 75     >=85  Women  7250.399009        SE 2010 (CSW)
## 76  [15,20)    Men    40.598662        SE 2010 (CSW)
## 77  [20,25)    Men    78.755315        SE 2010 (CSW)
## 78  [25,30)    Men    19.119003        SE 2010 (CSW)
## 79  [30,35)    Men    14.102527        SE 2010 (CSW)
## 80  [35,40)    Men    30.456613        SE 2010 (CSW)
## 81  [40,45)    Men    76.866091        SE 2010 (CSW)
## 82  [45,50)    Men   199.396834        SE 2010 (CSW)
## 83  [50,55)    Men   246.019683        SE 2010 (CSW)
## 84  [55,60)    Men   344.718246        SE 2010 (CSW)
## 85  [60,65)    Men   696.754791        SE 2010 (CSW)
## 86  [65,70)    Men  1299.106508        SE 2010 (CSW)
## 87  [70,75)    Men  1538.654401        SE 2010 (CSW)
## 88  [75,80)    Men  3302.448952        SE 2010 (CSW)
## 89  [80,85)    Men  5181.721530        SE 2010 (CSW)
## 90     >=85    Men 13284.599004        SE 2010 (CSW)
## 91  [15,20)  Women    20.639409 SE 2010 (unweighted)
## 92  [20,25)  Women    38.143917 SE 2010 (unweighted)
## 93  [25,30)  Women     8.166107 SE 2010 (unweighted)
## 94  [30,35)  Women    44.391832 SE 2010 (unweighted)
## 95  [35,40)  Women    50.256307 SE 2010 (unweighted)
## 96  [40,45)  Women    45.867352 SE 2010 (unweighted)
## 97  [45,50)  Women    96.508950 SE 2010 (unweighted)
## 98  [50,55)  Women   125.417752 SE 2010 (unweighted)
## 99  [55,60)  Women   233.759945 SE 2010 (unweighted)
## 100 [60,65)  Women   369.350910 SE 2010 (unweighted)
```

```
## 101 [65,70)  Women   534.364495 SE 2010 (unweighted)
## 102 [70,75)  Women   718.236012 SE 2010 (unweighted)
## 103 [75,80)  Women  1683.780501 SE 2010 (unweighted)
## 104 [80,85)  Women  2647.884340 SE 2010 (unweighted)
## 105    >=85  Women  7640.140785 SE 2010 (unweighted)
## 106 [15,20)    Men    39.237998 SE 2010 (unweighted)
## 107 [20,25)    Men    60.179334 SE 2010 (unweighted)
## 108 [25,30)    Men    17.125634 SE 2010 (unweighted)
## 109 [30,35)    Men    15.489708 SE 2010 (unweighted)
## 110 [35,40)    Men    29.587331 SE 2010 (unweighted)
## 111 [40,45)    Men    93.277988 SE 2010 (unweighted)
## 112 [45,50)    Men   172.683956 SE 2010 (unweighted)
## 113 [50,55)    Men   262.191924 SE 2010 (unweighted)
## 114 [55,60)    Men   362.957586 SE 2010 (unweighted)
## 115 [60,65)    Men   735.294118 SE 2010 (unweighted)
## 116 [65,70)    Men  1312.997626 SE 2010 (unweighted)
## 117 [70,75)    Men  1532.783714 SE 2010 (unweighted)
## 118 [75,80)    Men  3270.615905 SE 2010 (unweighted)
## 119 [80,85)    Men  4885.625530 SE 2010 (unweighted)
## 120    >=85    Men 13489.527867 SE 2010 (unweighted)
```

```r
p <- ggplot(data=output, aes(x=agecat, y=rate, color=type))
p1 <- p + geom_point(size=0.7) + geom_line(aes(group=type), size=0.3)
p1 <- p1 + scale_y_log10(breaks=c(5,10,20,100,1000,10000)) + facet_grid(female~.)
p1 <- p1 +  ylab("Mortality rate (per 100,000 person-years)") + xlab("Age categories")
p1 <- p1 + theme_bw()
p1 <- p1 + theme(strip.background = element_rect(fill="#F5F5F5"),
                 legend.key = element_blank(),
                 axis.text.x = element_text(angle=45, vjust=0.6))
p1 <- p1 + scale_color_brewer("", palette = "Set1")
p1
```

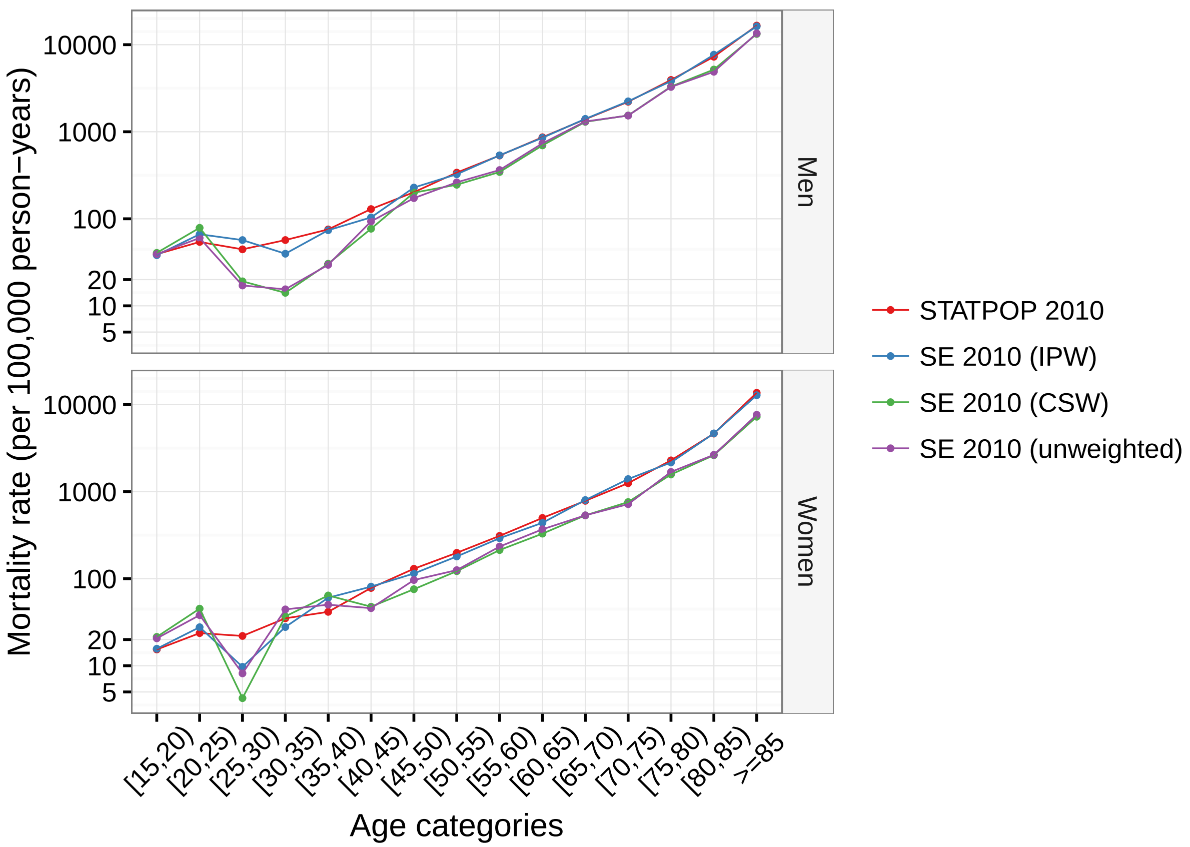

Mortality rate (per 100,000 person−years)
10000
1000
100
20
10
5
Men
Women
[15,20)
[20,25)
[25,30)
[30,35)
[35,40)
[40,45)
[45,50)
[50,55)
[55,60)
[60,65)
[65,70)
[70,75)
[75,80)
[80,85)
>=85
Age categories
STATPOP 2010
SE 2010 (IPW)
SE 2010 (CSW)
SE 2010 (unweighted)

Life expectancy (Stata code)

Own written Stata command based on:

Moser A., Clough-Gorr K., Zwahlen M. 2015. Modeling absolute differences in life expectancy with a censored skew-normal regression approach. PeerJ 3:e1162. DOI: 10.7717/peerj.1162.

Stata code and command is available at https://github.com/MoserGitHub/censn.

```
-----------------------------------------------------------------------------------
------
      name:  <unnamed>
       log:  F:\SNC\SurveyMortality\LE_Nov2017.txt
  log type:  text
 opened on:   4 Nov 2017, 14:50:14

.
. import delim using "F:\SNC\SurveyMortality\data\SwissCensus2010.csv", clear
(12 vars, 6,729,363 obs)

.
. adopath + "F:\SNC\SurveyMortality\ado\"
  [1]  (BASE)      "C:\Program Files (x86)\Stata14\ado\base/"
  [2]  (SITE)      "C:\Program Files (x86)\Stata14\ado\site/"
  [3]              "."
  [4]  (PERSONAL)  "c:\ado\personal/"
  [5]  (PLUS)      "c:\ado\plus/"
  [6]  (OLDPLACE)  "c:\ado/"
  [7]              "F:\SNC\SurveyMortality\ado\"

.
. replace agestart_rounded=agestart_rounded-30
(6,729,363 real changes made)

. replace ageend_rounded=ageend_rounded-30
(6,729,363 real changes made)

.
. * Restrict to individuals aged 30 years or older
. drop if agestart_rounded<0
(1,481,413 observations deleted)

.
. * STATPOP 2010
.
. censn ageend_rounded if female==0, lefttrun(agestart_rounded)
failure(died2011)

Fitting constant-only model:

Iteration 0:   log likelihood = -204064.16  (not concave)
Iteration 1:   log likelihood = -127946.07  (not concave)
Iteration 2:   log likelihood = -120713.33
Iteration 3:   log likelihood = -119590.76
Iteration 4:   log likelihood = -119490.28
Iteration 5:   log likelihood =  -119483.2
Iteration 6:   log likelihood = -119483.17
Iteration 7:   log likelihood = -119483.17

                                                Number of obs     =  2,546,113
                                                Wald chi2(0)      =          .
Log likelihood = -119483.17                     Prob > chi2       =          .

------------------------------------------------------------------------------
             |      Coef.   Std. Err.      z    P>|z|     [95% Conf. Interval]
-------------+----------------------------------------------------------------
mu           |
       _cons |   50.91508   .0568905   894.97   0.000     50.80358    51.02659
-------------+----------------------------------------------------------------
sigma        |
       _cons |   11.81383   .0443428   266.42   0.000     11.72692    11.90074
-------------+----------------------------------------------------------------
shape        |
       _cons |  -4.936167   .1105058   -44.67   0.000    -5.152754   -4.719579
------------------------------------------------------------------------------

Skewness parameter in CP
```

```
Coef.: -.84768077

Std. Err.: .00029225

[95% CI]: -.84825357 , -.84710797

. censn ageend_rounded if female==1, lefttrun(agestart_rounded)
failure(died2011)

Fitting constant-only model:

Iteration 0:   log likelihood = -204947.75  (not concave)
Iteration 1:   log likelihood = -197645.83  (not concave)
Iteration 2:   log likelihood = -124982.25
Iteration 3:   log likelihood = -121747.83
Iteration 4:   log likelihood = -120931.22
Iteration 5:   log likelihood = -120908.33
Iteration 6:   log likelihood = -120908.13
Iteration 7:   log likelihood = -120908.13

                                                Number of obs     =  2,701,837
                                                Wald chi2(0)      =          .
Log likelihood = -120908.13                     Prob > chi2       =          .

------------------------------------------------------------------------------
             |      Coef.   Std. Err.      z    P>|z|     [95% Conf. Interval]
-------------+----------------------------------------------------------------
mu           |
       _cons |   54.78439   .0525072  1043.37   0.000     54.68148     54.8873
-------------+----------------------------------------------------------------
sigma        |
       _cons |   10.93951   .0401325   272.58   0.000     10.86085    11.01817
-------------+----------------------------------------------------------------
shape        |
       _cons |  -5.189691   .1015395   -51.11   0.000    -5.388704   -4.990677
------------------------------------------------------------------------------

Skewness parameter in CP

Coef.: -.86013039

Std. Err.: .00022696

[95% CI]: -.86057523 , -.85968556

.
. * SE 2010
.
. * Unweighted
. censn ageend_rounded if female==0 & inse2010==1, lefttrun(agestart_rounded)
failure(
> died2011) difficult

Fitting constant-only model:

Iteration 0:   log likelihood = -9159.9159  (not concave)
Iteration 1:   log likelihood = -5627.0971  (not concave)
Iteration 2:   log likelihood =  -5606.496  (not concave)
Iteration 3:   log likelihood = -5321.1561  (not concave)
Iteration 4:   log likelihood = -5248.5118  (not concave)
Iteration 5:   log likelihood = -4964.3642  (not concave)
Iteration 6:   log likelihood = -4760.7278  (not concave)
Iteration 7:   log likelihood = -4711.1978
Iteration 8:   log likelihood = -4700.0464
Iteration 9:   log likelihood = -4699.5688
Iteration 10:  log likelihood = -4699.5669
Iteration 11:  log likelihood = -4699.5669
```

```
                                                Number of obs     =    121,021
                                                Wald chi2(0)      =          .
Log likelihood = -4699.5669                     Prob > chi2       =          .

------------------------------------------------------------------------------
             |      Coef.   Std. Err.      z    P>|z|     [95% Conf. Interval]
-------------+----------------------------------------------------------------
mu           |
       _cons |   53.32098   .2829822   188.43   0.000     52.76634    53.87561
-------------+----------------------------------------------------------------
sigma        |
       _cons |   11.60652   .2229127    52.07   0.000     11.16962    12.04343
-------------+----------------------------------------------------------------
shape        |
       _cons |  -5.181367   .7839979    -6.61   0.000    -6.717974   -3.644759
------------------------------------------------------------------------------

Skewness parameter in CP

Coef.: -.85974592

Std. Err.: .07764274

[95% CI]: -1.0119229 , -.70756894

. censn ageend_rounded if female==1 & inse2010==1, lefttrun(agestart_rounded)
failure(
> died2011) difficult

Fitting constant-only model:

Iteration 0:   log likelihood = -8933.3732  (not concave)
Iteration 1:   log likelihood = -4910.6544  (not concave)
Iteration 2:   log likelihood = -4737.8554  (not concave)
Iteration 3:   log likelihood = -4515.3801  (not concave)
Iteration 4:   log likelihood = -4455.4714  (not concave)
Iteration 5:   log likelihood = -4288.4417  (not concave)
Iteration 6:   log likelihood = -4246.4338  (not concave)
Iteration 7:   log likelihood = -4101.2046
Iteration 8:   log likelihood = -4054.2681
Iteration 9:   log likelihood = -4044.9889
Iteration 10:  log likelihood = -4044.8558
Iteration 11:  log likelihood = -4023.6899
Iteration 12:  log likelihood = -3983.7399
Iteration 13:  log likelihood =  -3975.368
Iteration 14:  log likelihood = -3973.2685
Iteration 15:  log likelihood = -3972.3757
Iteration 16:  log likelihood = -3972.2047
Iteration 17:  log likelihood = -3972.1824
Iteration 18:  log likelihood = -3972.1818
Iteration 19:  log likelihood = -3972.1818

                                                Number of obs     =    131,990
                                                Wald chi2(0)      =          .
Log likelihood = -3972.1818                     Prob > chi2       =          .

------------------------------------------------------------------------------
             |      Coef.   Std. Err.      z    P>|z|     [95% Conf. Interval]
-------------+----------------------------------------------------------------
mu           |
       _cons |   58.86656    .320969   183.40   0.000     58.23747    59.49565
-------------+----------------------------------------------------------------
sigma        |
       _cons |   11.50494   .2489221    46.22   0.000     11.01706    11.99282
-------------+----------------------------------------------------------------
shape        |
       _cons |  -10.89448   6.909487    -1.58   0.115    -24.43682    2.647869
```

```
------------------------------------------------------------------------------

Skewness parameter in CP

Coef.: -.96163395

Std. Err.: .91502505

[95% CI]: -2.7550501 , .83178219

.
. * CS weighted
. censn ageend_rounded [pw=weight2010] if female==0 & inse2010==1,
lefttrun(agestart_r
> ounded) failure(died2011) difficult

Fitting constant-only model:

Iteration 0:   log pseudolikelihood =  -185108.1  (not concave)
Iteration 1:   log pseudolikelihood = -117415.53  (not concave)
Iteration 2:   log pseudolikelihood = -113323.03  (not concave)
Iteration 3:   log pseudolikelihood = -109658.08  (not concave)
Iteration 4:   log pseudolikelihood = -105581.28  (not concave)
Iteration 5:   log pseudolikelihood = -103178.11  (not concave)
Iteration 6:   log pseudolikelihood = -100420.17  (not concave)
Iteration 7:   log pseudolikelihood = -96223.153
Iteration 8:   log pseudolikelihood = -96040.641  (backed up)
Iteration 9:   log pseudolikelihood = -94241.685
Iteration 10:  log pseudolikelihood = -94222.974
Iteration 11:  log pseudolikelihood = -94210.911
Iteration 12:  log pseudolikelihood =  -94210.89
Iteration 13:  log pseudolikelihood =  -94210.89

                                                Number of obs     =    121,021
                                                Wald chi2(0)      =          .
Log pseudolikelihood =  -94210.89               Prob > chi2       =          .

------------------------------------------------------------------------------
             |               Robust
             |      Coef.   Std. Err.      z    P>|z|     [95% Conf. Interval]
-------------+----------------------------------------------------------------
mu           |
       _cons |   53.35384   .3199026   166.78   0.000     52.72684    53.98084
-------------+----------------------------------------------------------------
sigma        |
       _cons |   11.58996   .2629378    44.08   0.000     11.07461    12.10531
-------------+----------------------------------------------------------------
shape        |
       _cons |  -4.435974   .4977457    -8.91   0.000    -5.411538    -3.46041
------------------------------------------------------------------------------

Skewness parameter in CP

Coef.: -.81784394

Std. Err.: .02362381

[95% CI]: -.86414576 , -.77154213

. censn ageend_rounded [pw=weight2010] if female==1 & inse2010==1,
lefttrun(agestart_r
> ounded) failure(died2011) difficult

Fitting constant-only model:

Iteration 0:   log pseudolikelihood = -173967.84  (not concave)
Iteration 1:   log pseudolikelihood = -92477.105  (not concave)
Iteration 2:   log pseudolikelihood = -90265.987  (not concave)
```

```
Iteration 3:   log pseudolikelihood = -84392.726  (not concave)
Iteration 4:   log pseudolikelihood = -84378.809  (not concave)
Iteration 5:   log pseudolikelihood = -81224.765  (not concave)
Iteration 6:   log pseudolikelihood = -79049.605  (not concave)
Iteration 7:   log pseudolikelihood = -76734.701
Iteration 8:   log pseudolikelihood = -76305.367
Iteration 9:   log pseudolikelihood =  -76038.98
Iteration 10:  log pseudolikelihood = -76037.754
Iteration 11:  log pseudolikelihood = -76037.691
Iteration 12:  log pseudolikelihood = -76037.688
Iteration 13:  log pseudolikelihood = -75585.041
Iteration 14:  log pseudolikelihood = -74925.628
Iteration 15:  log pseudolikelihood = -74804.441
Iteration 16:  log pseudolikelihood = -74765.511
Iteration 17:  log pseudolikelihood = -74757.048
Iteration 18:  log pseudolikelihood = -74754.789
Iteration 19:  log pseudolikelihood = -74754.544
Iteration 20:  log pseudolikelihood = -74754.537
Iteration 21:  log pseudolikelihood = -74754.537

                                                Number of obs     =    131,990
                                                Wald chi2(0)      =          .
Log pseudolikelihood = -74754.537               Prob > chi2       =          .

------------------------------------------------------------------------------
             |               Robust
             |      Coef.   Std. Err.      z    P>|z|     [95% Conf. Interval]
-------------+----------------------------------------------------------------
mu           |
       _cons |   59.19851   .3831018   154.52   0.000     58.44764    59.94937
-------------+----------------------------------------------------------------
sigma        |
       _cons |   11.49247   .3472371    33.10   0.000      10.8119    12.17305
-------------+----------------------------------------------------------------
shape        |
       _cons |  -8.634522   3.368181    -2.56   0.010    -15.23604   -2.033009
------------------------------------------------------------------------------

Skewness parameter in CP

Coef.: -.94260365

Std. Err.: .71855036

[95% CI]: -2.3509365 , .46572918

.
. * IP weighted
. censn ageend_rounded [pw=ipw2010] if female==0 & inse2010==1, 
lefttrun(agestart_roun
> ded) failure(died2011) difficult

Fitting constant-only model:

Iteration 0:   log pseudolikelihood = -202293.98  (not concave)
Iteration 1:   log pseudolikelihood = -135357.74  (not concave)
Iteration 2:   log pseudolikelihood = -133832.29  (not concave)
Iteration 3:   log pseudolikelihood = -126260.43  (not concave)
Iteration 4:   log pseudolikelihood =    -122469
Iteration 5:   log pseudolikelihood = -119868.08
Iteration 6:   log pseudolikelihood = -118332.94
Iteration 7:   log pseudolikelihood = -118293.09
Iteration 8:   log pseudolikelihood = -118292.42
Iteration 9:   log pseudolikelihood = -118292.42

                                                Number of obs     =    121,021
                                                Wald chi2(0)      =          .
Log pseudolikelihood = -118292.42               Prob > chi2       =          .
```

```
------------------------------------------------------------------------------
             |               Robust
             |      Coef.   Std. Err.      z    P>|z|     [95% Conf. Interval]
-------------+----------------------------------------------------------------
mu           |
       _cons |   50.92614   .3478712   146.39   0.000     50.24433    51.60796
-------------+----------------------------------------------------------------
sigma        |
       _cons |   11.71864   .3027535    38.71   0.000     11.12525    12.31202
-------------+----------------------------------------------------------------
shape        |
       _cons |  -4.373761    .445647    -9.81   0.000    -5.247213   -3.500309
------------------------------------------------------------------------------

Skewness parameter in CP

Coef.: -.81355052

Std. Err.: .01738051

[95% CI]: -.84761569 , -.77948535

. censn ageend_rounded [pw=ipw2010] if female==1 & inse2010==1,
lefttrun(agestart_roun
> ded) failure(died2011) difficult

Fitting constant-only model:

Iteration 0:   log pseudolikelihood = -204835.01  (not concave)
Iteration 1:   log pseudolikelihood = -142195.92  (not concave)
Iteration 2:   log pseudolikelihood = -130641.31  (not concave)
Iteration 3:   log pseudolikelihood = -120798.93  (not concave)
Iteration 4:   log pseudolikelihood = -117473.54  (not concave)
Iteration 5:   log pseudolikelihood = -116953.74
Iteration 6:   log pseudolikelihood = -116720.59  (not concave)
Iteration 7:   log pseudolikelihood = -116161.54
Iteration 8:   log pseudolikelihood =  -116139.6
Iteration 9:   log pseudolikelihood = -116131.58
Iteration 10:  log pseudolikelihood = -116131.56
Iteration 11:  log pseudolikelihood = -116131.56

                                                Number of obs     =    131,990
                                                Wald chi2(0)      =          .
Log pseudolikelihood = -116131.56               Prob > chi2       =          .

------------------------------------------------------------------------------
             |               Robust
             |      Coef.   Std. Err.      z    P>|z|     [95% Conf. Interval]
-------------+----------------------------------------------------------------
mu           |
       _cons |   54.80675   .3479427   157.52   0.000      54.1248    55.48871
-------------+----------------------------------------------------------------
sigma        |
       _cons |   10.70856   .2874598    37.25   0.000     10.14515    11.27197
-------------+----------------------------------------------------------------
shape        |
       _cons |  -6.421754   .7594985    -8.46   0.000    -7.910343   -4.933164
------------------------------------------------------------------------------

Skewness parameter in CP

Coef.: -.90335602

Std. Err.: .07179521

[95% CI]: -1.044072 , -.76264
```

```
.
. replace agestart_rounded=agestart_rounded-35
(5,247,950 real changes made)

. replace ageend_rounded=ageend_rounded-35
(5,247,950 real changes made)

.
. * Restrict to individuals aged 65 years or older
. drop if agestart_rounded<0
(3,910,542 observations deleted)

.
. * STATPOP 2010
.
. censn ageend_rounded if female==0, lefttrun(agestart_rounded)
failure(died2011)

Fitting constant-only model:

Iteration 0:   log likelihood =  -102837.2  (not concave)
Iteration 1:   log likelihood = -98421.569  (not concave)
Iteration 2:   log likelihood = -94910.871  (not concave)
Iteration 3:   log likelihood = -93907.237  (not concave)
Iteration 4:   log likelihood = -91186.706  (not concave)
Iteration 5:   log likelihood = -84202.104  (not concave)
Iteration 6:   log likelihood = -83998.888
Iteration 7:   log likelihood = -83731.752
Iteration 8:   log likelihood = -83718.549
Iteration 9:   log likelihood = -83718.462
Iteration 10:  log likelihood = -83718.462

                                                Number of obs     =    575,143
                                                Wald chi2(0)      =          .
Log likelihood = -83718.462                     Prob > chi2       =          .

------------------------------------------------------------------------------
             |      Coef.   Std. Err.      z    P>|z|     [95% Conf. Interval]
-------------+----------------------------------------------------------------
mu           |
       _cons |   17.62199   .0851398   206.98   0.000     17.45512    17.78886
-------------+----------------------------------------------------------------
sigma        |
       _cons |   9.904003   .0824305   120.15   0.000     9.742442    10.06556
-------------+----------------------------------------------------------------
shape        |
       _cons |   -3.33546   .0985455   -33.85   0.000    -3.528606   -3.142315
------------------------------------------------------------------------------

Skewness parameter in CP

Coef.: -.71442172

Std. Err.: .00020754

[95% CI]: -.71482849 , -.71401495

. censn ageend_rounded if female==1, lefttrun(agestart_rounded)
failure(died2011)

Fitting constant-only model:

Iteration 0:   log likelihood =  -122946.3  (not concave)
Iteration 1:   log likelihood =  -100337.4  (not concave)
Iteration 2:   log likelihood = -98131.809  (not concave)
Iteration 3:   log likelihood = -97824.239
Iteration 4:   log likelihood = -97492.706
Iteration 5:   log likelihood = -97486.484
```

```
Iteration 6:   log likelihood = -97486.482

                                                Number of obs     =    762,265
                                                Wald chi2(0)      =          .
Log likelihood = -97486.482                     Prob > chi2       =          .

------------------------------------------------------------------------------
             |      Coef.   Std. Err.      z    P>|z|     [95% Conf. Interval]
-------------+----------------------------------------------------------------
mu           |
       _cons |    21.6647   .0562523   385.13   0.000     21.55445    21.77496
-------------+----------------------------------------------------------------
sigma        |
       _cons |    8.82385   .0511099   172.64   0.000     8.723677    8.924024
-------------+----------------------------------------------------------------
shape        |
       _cons |  -3.321786   .0746158   -44.52   0.000     -3.46803   -3.175542
------------------------------------------------------------------------------

Skewness parameter in CP

Coef.: -.71267205

Std. Err.: .00009029

[95% CI]: -.71284902 , -.71249508

.
. * SE 2010
.
. * Unweighted
. censn ageend_rounded if female==0 & inse2010==1, lefttrun(agestart_rounded)
failure(
> died2011) difficult

Fitting constant-only model:

Iteration 0:   log likelihood = -4710.5291  (not concave)
Iteration 1:   log likelihood = -3605.2863  (not concave)
Iteration 2:   log likelihood =  -3568.683  (not concave)
Iteration 3:   log likelihood =  -3511.469  (not concave)
Iteration 4:   log likelihood = -3409.5631
Iteration 5:   log likelihood = -3385.0388
Iteration 6:   log likelihood = -3384.4301
Iteration 7:   log likelihood = -3384.3049
Iteration 8:   log likelihood = -3384.3048

                                                Number of obs     =     27,987
                                                Wald chi2(0)      =          .
Log likelihood = -3384.3048                     Prob > chi2       =          .

------------------------------------------------------------------------------
             |      Coef.   Std. Err.      z    P>|z|     [95% Conf. Interval]
-------------+----------------------------------------------------------------
mu           |
       _cons |   19.31354    .394144    49.00   0.000     18.54103    20.08605
-------------+----------------------------------------------------------------
sigma        |
       _cons |   10.35302   .3984034    25.99   0.000      9.57216    11.13387
-------------+----------------------------------------------------------------
shape        |
       _cons |  -3.995615   .6438635    -6.21   0.000    -5.257564   -2.733665
------------------------------------------------------------------------------

Skewness parameter in CP

Coef.: -.78404767
```

```
Std. Err.: .04714702

[95% CI]: -.87645412 , -.69164121

. censn ageend_rounded if female==1 & inse2010==1, lefttrun(agestart_rounded)
failure(
> died2011) difficult

Fitting constant-only model:

Iteration 0:   log likelihood = -5247.3335  (not concave)
Iteration 1:   log likelihood = -3242.8946  (not concave)
Iteration 2:   log likelihood = -3165.9234  (not concave)
Iteration 3:   log likelihood = -3108.3634
Iteration 4:   log likelihood = -3089.9284
Iteration 5:   log likelihood =   -3084.47
Iteration 6:   log likelihood = -3084.4142
Iteration 7:   log likelihood = -3084.4118
Iteration 8:   log likelihood = -3084.4117
Iteration 9:   log likelihood = -3074.3715
Iteration 10:  log likelihood = -3068.4501
Iteration 11:  log likelihood = -3059.6613
Iteration 12:  log likelihood = -3059.5725
Iteration 13:  log likelihood =  -3059.572
Iteration 14:  log likelihood =  -3059.572

                                                Number of obs     =     36,669
                                                Wald chi2(0)      =          .
Log likelihood =  -3059.572                     Prob > chi2       =          .

------------------------------------------------------------------------------
             |      Coef.   Std. Err.      z    P>|z|     [95% Conf. Interval]
-------------+----------------------------------------------------------------
mu           |
       _cons |   25.17702   .2873424    87.62   0.000     24.61384     25.7402
-------------+----------------------------------------------------------------
sigma        |
       _cons |   9.040391   .2794137    32.35   0.000      8.49275    9.588031
-------------+----------------------------------------------------------------
shape        |
       _cons |  -4.688725   1.234264    -3.80   0.000    -7.107838   -2.269612
------------------------------------------------------------------------------

Skewness parameter in CP

Coef.: -.83389166

Std. Err.: .21169414

[95% CI]: -1.2488045 , -.41897878

.
. * CS weighted
. censn ageend_rounded [pw=weight2010] if female==0 & inse2010==1,
lefttrun(agestart_r
> ounded) failure(died2011) difficult

Fitting constant-only model:

Iteration 0:   log pseudolikelihood = -93569.748  (not concave)
Iteration 1:   log pseudolikelihood = -69745.151  (not concave)
Iteration 2:   log pseudolikelihood = -68316.215  (not concave)
Iteration 3:   log pseudolikelihood = -67773.325
Iteration 4:   log pseudolikelihood = -67684.126
Iteration 5:   log pseudolikelihood = -67675.956
Iteration 6:   log pseudolikelihood = -67675.945
Iteration 7:   log pseudolikelihood = -67675.945
```

```
                                                Number of obs     =     27,987
                                                Wald chi2(0)      =          .
Log pseudolikelihood = -67675.945               Prob > chi2       =          .

------------------------------------------------------------------------------
             |               Robust
             |      Coef.   Std. Err.      z    P>|z|     [95% Conf. Interval]
-------------+----------------------------------------------------------------
mu           |
       _cons |   19.37432   .4233577    45.76   0.000     18.54456    20.20409
-------------+----------------------------------------------------------------
sigma        |
       _cons |   10.27155   .4361618    23.55   0.000      9.41669    11.12641
-------------+----------------------------------------------------------------
shape        |
       _cons |  -3.334177   .4582202    -7.28   0.000    -4.232273   -2.436082
------------------------------------------------------------------------------

Skewness parameter in CP

Coef.: -.71425818

Std. Err.: .01878486

[95% CI]: -.75107582 , -.67744054

. censn ageend_rounded [pw=weight2010] if female==1 & inse2010==1,
lefttrun(agestart_r
> ounded) failure(died2011) difficult

Fitting constant-only model:

Iteration 0:   log pseudolikelihood = -100139.91  (not concave)
Iteration 1:   log pseudolikelihood = -60542.492  (not concave)
Iteration 2:   log pseudolikelihood = -59160.972  (not concave)
Iteration 3:   log pseudolikelihood = -58171.492
Iteration 4:   log pseudolikelihood = -58069.301  (not concave)
Iteration 5:   log pseudolikelihood = -57827.926
Iteration 6:   log pseudolikelihood = -57580.745
Iteration 7:   log pseudolikelihood = -57566.531
Iteration 8:   log pseudolikelihood = -57566.386
Iteration 9:   log pseudolikelihood = -57566.385

                                                Number of obs     =     36,669
                                                Wald chi2(0)      =          .
Log pseudolikelihood = -57566.385               Prob > chi2       =          .

------------------------------------------------------------------------------
             |               Robust
             |      Coef.   Std. Err.      z    P>|z|     [95% Conf. Interval]
-------------+----------------------------------------------------------------
mu           |
       _cons |   25.30224   .3263267    77.54   0.000     24.66265    25.94183
-------------+----------------------------------------------------------------
sigma        |
       _cons |   9.074467   .3335205    27.21   0.000     8.420779    9.728155
-------------+----------------------------------------------------------------
shape        |
       _cons |  -4.605421   .8123849    -5.67   0.000    -6.197667   -3.013176
------------------------------------------------------------------------------

Skewness parameter in CP

Coef.: -.82883757

Std. Err.: .08467034

[95% CI]: -.99478839 , -.66288674
```

```
.
. * IP weighted
. censn ageend_rounded [pw=ipw2010] if female==0 & inse2010==1, 
lefttrun(agestart_roun
> ded) failure(died2011) difficult

Fitting constant-only model:

Iteration 0:   log pseudolikelihood = -103939.86  (not concave)
Iteration 1:   log pseudolikelihood = -83973.915  (not concave)
Iteration 2:   log pseudolikelihood = -83633.612
Iteration 3:   log pseudolikelihood = -83208.641
Iteration 4:   log pseudolikelihood = -83127.167
Iteration 5:   log pseudolikelihood = -83125.703
Iteration 6:   log pseudolikelihood = -83125.703

                                                Number of obs     =     27,987
                                                Wald chi2(0)      =          .
Log pseudolikelihood = -83125.703               Prob > chi2       =          .

------------------------------------------------------------------------------
             |               Robust
             |      Coef.   Std. Err.      z    P>|z|     [95% Conf. Interval]
-------------+----------------------------------------------------------------
mu           |
       _cons |    17.5884   .4590447    38.32   0.000     16.68869    18.48811
-------------+----------------------------------------------------------------
sigma        |
       _cons |   9.857571   .4486002    21.97   0.000     8.978331    10.73681
-------------+----------------------------------------------------------------
shape        |
       _cons |  -2.960692   .4322386    -6.85   0.000    -3.807864    -2.11352
------------------------------------------------------------------------------

Skewness parameter in CP

Coef.: -.66080965

Std. Err.: .0159538

[95% CI]: -.69207853 , -.62954078

. censn ageend_rounded [pw=ipw2010] if female==1 & inse2010==1, 
lefttrun(agestart_roun
> ded) failure(died2011) difficult

Fitting constant-only model:

Iteration 0:   log pseudolikelihood = -126311.64  (not concave)
Iteration 1:   log pseudolikelihood = -99049.304  (not concave)
Iteration 2:   log pseudolikelihood = -95606.852
Iteration 3:   log pseudolikelihood = -95441.279
Iteration 4:   log pseudolikelihood =  -95379.23
Iteration 5:   log pseudolikelihood = -95379.186
Iteration 6:   log pseudolikelihood = -94113.993
Iteration 7:   log pseudolikelihood = -93961.868
Iteration 8:   log pseudolikelihood =  -93956.38
Iteration 9:   log pseudolikelihood = -93956.375

                                                Number of obs     =     36,669
                                                Wald chi2(0)      =          .
Log pseudolikelihood = -93956.375               Prob > chi2       =          .

------------------------------------------------------------------------------
             |               Robust
             |      Coef.   Std. Err.      z    P>|z|     [95% Conf. Interval]
-------------+----------------------------------------------------------------
```

```
mu           |
       _cons |   21.56338   .3762807    57.31   0.000     20.82589    22.30088
-------------+----------------------------------------------------------------
sigma        |
       _cons |   8.766594   .3525496    24.87   0.000     8.075609    9.457578
-------------+----------------------------------------------------------------
shape        |
       _cons |  -4.341851    .610001    -7.12   0.000    -5.537431   -3.146271
------------------------------------------------------------------------------

Skewness parameter in CP

Coef.: -.81129162

Std. Err.: .04091274

[95% CI]: -.89147911 , -.73110412

.
. log close
      name:  <unnamed>
       log:  F:\SNC\SurveyMortality\LE_Nov2017.txt
  log type:  text
 closed on:   4 Nov 2017, 18:27:00
--------------------------------------------------------------------------------
------
```

Construction of inverse probability weights (Stata code)

Note that, because of privacy preserving issues, we were not allowed to provide the exact date of death (variable dod below). Thus, the Nelson-Aalen estimator for the anonymised dataset is slightly different, than from the one used in the analysis with exact date information.

Original code:

```
gen statpop_enddate2011=d(31.12.2011)

replace statpop_enddate2011=dod if dod<=d(31.12.2011)

stset statpop_enddate2011, origin(statpop_startdate)
failure(died2011) scale(365.25)
```

Reproducible code:

```
stset endage_rounded, origin(startageage_rounded)
failure(died2011)
```

```
use "F:/SNC/SurveyMortality/data/SwissCensuses.csv", clear

### Note that, because of privacy preserving issues, we were not allowed to
### provide the exact date of death (variable dod below). Thus, the
### Nelson-Aalen estimator is not exactly the same as in
### the original analysis.

### Original code:

### gen statpop_enddate2011=d(31.12.2011)
### replace statpop_enddate2011=dod if dod<=d(31.12.2011)

### stset statpop_enddate2011, origin(statpop_startdate) failure(died2011)
###      scale(365.25)

### Reproducible code:

stset endage_rounded, origin(startageage_rounded), failure(died2011)

### Nelson-Aalen estimator
sts gen na2011=na

drop _st _d _t _t0 statpop_enddate2011

*** Use only STATPOP/SE 2010
keep if statyear==2010

logistic inse2010 female##statpop_agecat5##i.died2011 na2011 ///
        i.r10_canton_se i.r10_nat i.r10_civil
predict prob

gen ipw2010=1/prob

drop prob1 na2011
```
