# Supplementary material for: Calibration adjustments to address bias in mortality analyses due to informative sampling—a census-linked survey analysis in Switzerland
Source: PeerJ. 2018 Feb 13;6:e4376. doi: 10.7717/peerj.4376 (PMC5815334; doi:10.7717/peerj.4376)
Supplement: Supplemental Information 2 — Statistical and analysis for main results and supplementary results using exact date information. [file peerj-06-4376-s008.pdf]

Calibration adjustments to address bias in mortality analyses due to informative sampling - a census-linked survey analysis in Switzerland

André Moser

November 06, 2017

This documents provides the statistical analysis code for the main results (Table 1, Table 2, Figure 1), Supplemental Tables 1-3, and Supplemental Figures 1/2, in the manuscript. We used an extended dataset with STATPOP/SE population of the years 2011-2013, exact date of death, and addtional variables collected in the SE, compared to the provided anonymised dataset. Figure 2 is shown in a seperate file.

```
library(reporttools)
library(rms)
library(ggplot2)
library(foreign)
library(survey)

data <- read.dta( paste(path, "SwissCensuses.dta", sep=""))
```

There are 6,729,363 individuals in the STATPOP 2010,

```
sum(data$statpop2010==1, na.rm=T)
```

```
## [1] 6729363
```

6,731,420 individuals in the STATPOP 2011,

```
sum(data$statpop2011==1, na.rm=T)
```

```
## [1] 6731420
```

6,728,505 individuals in the STATPOP 2012,

```
sum(data$statpop2012==1, na.rm=T)
```

```
## [1] 6728505
```

6,733,393 individuals in the STATPOP 2013,

```
sum(data$statpop2013==1, na.rm=T)
```

```
## [1] 6733393
```

older than 15 years.

Table 1

```r
tableNominal(vars=data[,c("female", "r10_statpop_agecat5", "r10_civil", "r10_nat",
            "r10_canton_se")], group=data$died2011, cumsum=F,
            subset=data$statpop2010==1,
            cap="STATPOP 2010: Categorical variables")
```

Variable	Levels	n_0	$\%_0$	n_1	$\%_1$	n_{all}	$\%_{all}$
female	0	3270394	49.0	29804	48.4	3300198	49.0
	1	3397430	51.0	31735	51.6	3429165	51.0
	all	6667824	100.0	61539	100.0	6729363	100.0
r10_statpop_agecat5	15-	453418	6.8	125	0.2	453543	6.7
	20-	497932	7.5	195	0.3	498127	7.4
	25-	535327	8.0	179	0.3	535506	8.0
	30-	542177	8.1	250	0.4	542427	8.1
	35-	562531	8.4	330	0.5	562861	8.4
	40-	636715	9.6	662	1.1	637377	9.5
	45-	651880	9.8	1087	1.8	652967	9.7
	50-	565305	8.5	1525	2.5	566830	8.4
	55-	484779	7.3	2048	3.3	486827	7.2
	60-	456872	6.8	3110	5.0	459982	6.8
	65-	393251	5.9	4259	6.9	397510	5.9
	70-	298540	4.5	5082	8.3	303622	4.5
	75-	248981	3.7	7565	12.3	256546	3.8
	80-	184460	2.8	10711	17.4	195171	2.9
	85-	155656	2.3	24411	39.7	180067	2.7
	120-	0	0.0	0	0.0	0	0.0
	all	6667824	100.0	61539	100.0	6729363	100.0
r10_civil	Single	2224776	33.4	7455	12.1	2232231	33.2
	Married	3449816	51.7	24937	40.5	3474753	51.6
	Widowed	387352	5.8	23109	37.5	410461	6.1
	Other	605880	9.1	6038	9.8	611918	9.1
	all	6667824	100.0	61539	100.0	6729363	100.0
r10_nat	CHE	5147117	77.2	56418	91.7	5203535	77.3
	EU/EFTA	989417	14.8	4309	7.0	993726	14.8
	Other Europe	317233	4.8	538	0.9	317771	4.7
	Other world	214057	3.2	274	0.4	214331	3.2
	all	6667824	100.0	61539	100.0	6729363	100.0
r10_canton_se	ZH	837660	12.6	6681	10.9	844341	12.6
	BE	689868	10.3	7072	11.5	696940	10.4
	LU	318109	4.8	2750	4.5	320859	4.8
	UR	29375	0.4	315	0.5	29690	0.4
	SZ	122585	1.8	1053	1.7	123638	1.8
	OW	29675	0.4	249	0.4	29924	0.4
	NW	34471	0.5	270	0.4	34741	0.5
	GL	32785	0.5	364	0.6	33149	0.5
	ZG	94883	1.4	704	1.1	95587	1.4
	FR	228838	3.4	1948	3.2	230786	3.4
	SO	216854	3.2	2161	3.5	219015	3.2
	BS	162021	2.4	2026	3.3	164047	2.4
	BL	234029	3.5	2195	3.6	236224	3.5
	SH	65688	1.0	713	1.2	66401	1.0
	AR	43940	0.7	444	0.7	44384	0.7
	AI	12738	0.2	135	0.2	12873	0.2
	SG	405528	6.1	3657	5.9	409185	6.1

GR	174072	2.6	1615	2.6	175687	2.6
AG	513802	7.7	4350	7.1	518152	7.7
TG	209311	3.1	1881	3.1	211192	3.1
TI	284417	4.3	2856	4.6	287273	4.3
VD	603119	9.1	5165	8.4	608284	9.0
VS	265481	4.0	2492	4.0	267973	4.0
NE	143272	2.1	1501	2.4	144773	2.1
GE	365634	5.5	3013	4.9	368647	5.5
JU	57600	0.9	642	1.0	58242	0.9
BE-JU	43374	0.6	518	0.8	43892	0.6
City Bern	113968	1.7	1331	2.2	115299	1.7
City Zurich	334727	5.0	3438	5.6	338165	5.0
all	6667824	100.0	61539	100.0	6729363	100.0

Table 1: STATPOP 2010: Categorical variables

```
tableNominal(vars=data[,c("female", "r10_statpop_agecat5", "r10_civil", "r10_nat",
          "r10_canton_se", "inse2010")], group=data$died2011,
          subset=data$inse2010==1, cumsum=F,
          cap="SE 2010: Categorical variables")
```

**Variable**	**Levels**	$\mathbf{n}_0$	$\%_0$	$\mathbf{n}_1$	$\%_1$	$\mathbf{n}_{\text{all}}$	$\%_{\text{all}}$
female	0	151684	48.1	1099	55.8	152783	48.2
	1	163424	51.9	872	44.2	164296	51.8
	all	315108	100.0	1971	100.0	317079	100.0
r10_statpop_agecat5	15-	19890	6.3	6	0.3	19896	6.3
	20-	20467	6.5	10	0.5	20477	6.5
	25-	23935	7.6	3	0.1	23938	7.5
	30-	26445	8.4	8	0.4	26453	8.3
	35-	27456	8.7	11	0.6	27467	8.7
	40-	30282	9.6	21	1.1	30303	9.6
	45-	31172	9.9	42	2.1	31214	9.8
	50-	26896	8.5	52	2.6	26948	8.5
	55-	23522	7.5	70	3.5	23592	7.4
	60-	22204	7.0	122	6.2	22326	7.0
	65-	19730	6.3	180	9.1	19910	6.3
	70-	15000	4.8	163	8.3	15163	4.8
	75-	12520	4.0	300	15.2	12820	4.0
	80-	9040	2.9	323	16.4	9363	3.0
	85-	6549	2.1	660	33.5	7209	2.3
	120-	0	0.0	0	0.0	0	0.0
	all	315108	100.0	1971	100.0	317079	100.0
r10_civil	Single	101868	32.3	189	9.6	102057	32.2
	Married	166452	52.8	999	50.7	167451	52.8
	Widowed	17942	5.7	592	30.0	18534	5.8
	Other	28846	9.2	191	9.7	29037	9.2
	all	315108	100.0	1971	100.0	317079	100.0
r10_nat	CHE	249184	79.1	1779	90.3	250963	79.2
	EU/EFTA	45200	14.3	172	8.7	45372	14.3
	Other Europe	12503	4.0	14	0.7	12517	4.0
	Other world	8221	2.6	6	0.3	8227	2.6
	all	315108	100.0	1971	100.0	317079	100.0
r10_canton_se	ZH	24948	7.9	119	6.0	25067	7.9
	BE	21957	7.0	130	6.6	22087	7.0
	LU	19208	6.1	96	4.9	19304	6.1
	UR	976	0.3	5	0.2	981	0.3

	SZ	3589	1.1	19	1.0	3608	1.1
	OW	828	0.3	4	0.2	832	0.3
	NW	1039	0.3	6	0.3	1045	0.3
	GL	914	0.3	7	0.4	921	0.3
	ZG	5205	1.6	23	1.2	5228	1.6
	FR	6570	2.1	39	2.0	6609	2.1
	SO	6598	2.1	54	2.7	6652	2.1
	BS	5113	1.6	26	1.3	5139	1.6
	BL	7366	2.3	44	2.2	7410	2.3
	SH	2001	0.6	18	0.9	2019	0.6
	AR	1382	0.4	15	0.8	1397	0.4
	AI	381	0.1	2	0.1	383	0.1
	SG	12174	3.9	57	2.9	12231	3.9
	GR	5070	1.6	32	1.6	5102	1.6
	AG	30046	9.5	164	8.3	30210	9.5
	TG	12137	3.8	73	3.7	12210	3.8
	TI	17343	5.5	123	6.2	17466	5.5
	VD	34465	10.9	232	11.8	34697	10.9
	VS	7112	2.3	49	2.5	7161	2.3
	NE	9295	3.0	73	3.7	9368	3.0
	GE	20510	6.5	112	5.7	20622	6.5
	JU	3723	1.2	30	1.5	3753	1.2
	BE-JU	2899	0.9	22	1.1	2921	0.9
	City Bern	13139	4.2	111	5.6	13250	4.2
	City Zurich	39120	12.4	286	14.5	39406	12.4
	all	315108	100.0	1971	100.0	317079	100.0
inse2010	1	315108	100.0	1971	100.0	317079	100.0
	all	315108	100.0	1971	100.0	317079	100.0

Table 2: SE 2010: Categorical variables

```
tableContinuous(vars=data[,c("weight2010", "ipw20102011")], group=data$died2011,
                subset=data$statpop2010==1, cap="STATPOP 2010: Continuous variables")
```

Variable	Levels	n	Min	q_1	$\tilde{x}$	$\bar{x}$	q_3	Max	s	IQR	#NA
weight2010	0	315108	3.3	15.0	16.8	20.6	30.3	75.0	9.4	15.3	6352716
	1	1971	6.4	14.6	16.1	19.4	30.1	43.4	9.3	15.5	59568
		NA	NA	NA	NA	NA	NA	NA	NA	NA	NA
	all	317079	3.3	15.0	16.8	20.6	30.3	75.0	9.4	15.3	6965390
ipw20102011	0	6667824	7.3	16.6	28.8	25.6	33.1	67.9	9.8	16.5	0
	1	61539	5.3	25.2	38.2	42.1	54.8	226.0	22.1	29.6	0
		NA	NA	NA	NA	NA	NA	NA	NA	NA	NA
	all	6729363	5.3	16.7	28.8	25.7	33.3	226.0	10.1	16.6	553106

Table 3: STATPOP 2010: Continuous variables

```
tableContinuous(vars=data[,c("weight2010", "ipw20102011")], group=data$died2011,
                subset=data$inse2010==1, cap="SE 2010: Continuous variables")
```

Variable	Levels	n	Min	q_1	$\tilde{x}$	$\bar{x}$	q_3	Max	s	IQR	#NA
weight2010	0	315108	3.3	15.0	16.8	20.6	30.3	75.0	9.4	15.3	0
	1	1971	6.4	14.6	16.1	19.4	30.1	43.4	9.3	15.5	0
	all	317079	3.3	15.0	16.8	20.6	30.3	75.0	9.4	15.3	0
ipw20102011	0	315108	7.3	15.2	17.6	21.2	30.2	61.8	9.6	15.0	0
	1	1971	5.8	17.0	26.0	30.2	39.3	113.4	17.3	22.3	0

all	317079	5.8	15.2	17.6	21.2	30.2	113.4	9.7	15.0	0

Table 4: SE 2010: Continuous variables

Table 2

```
output <- c()

mod <- glm(inse2010 ~ r10_statpop_agecat5, data=data,
           subset=data$statpop2010==1, family = binomial())

output <- rbind(output,
                data.frame(var=names(mod$coefficients[2:length(mod$coefficients)]),
                           est=exp(mod$coefficients[2:length(mod$coefficients)]),
                           lci=exp(confint.default(mod)[2:length(mod$coefficients),1]),
                           uci=exp(confint.default(mod)[2:length(mod$coefficients),2])))
rm(mod)

mod <- glm(inse2010 ~ female, data=data,
           subset=data$statpop2010==1, family = binomial())

output <- rbind(output,
                data.frame(var=names(mod$coefficients[2:length(mod$coefficients)]),
                           est=exp(mod$coefficients[2:length(mod$coefficients)]),
                           lci=exp(confint.default(mod)[2:length(mod$coefficients),1]),
                           uci=exp(confint.default(mod)[2:length(mod$coefficients),2])))
rm(mod)

mod <- glm(inse2010 ~ r10_nat, data=data,
           subset=data$statpop2010==1, family = binomial())

output <- rbind(output,
                data.frame(var=names(mod$coefficients[2:length(mod$coefficients)]),
                           est=exp(mod$coefficients[2:length(mod$coefficients)]),
                           lci=exp(confint.default(mod)[2:length(mod$coefficients),1]),
                           uci=exp(confint.default(mod)[2:length(mod$coefficients),2])))
rm(mod)

mod <- glm(inse2010 ~ r10_civil, data=data,
           subset=data$statpop2010==1, family = binomial())

output <- rbind(output,
                data.frame(var=names(mod$coefficients[2:length(mod$coefficients)]),
                           est=exp(mod$coefficients[2:length(mod$coefficients)]),
                           lci=exp(confint.default(mod)[2:length(mod$coefficients),1]),
```

```
                           uci=exp(confint.default(mod)[2:length(mod$coefficients),2])))
rm(mod)

mod <- glm(inse2010 ~ r10_canton_se, data=data,
           subset=data$statpop2010==1, family = binomial())

output <- rbind(output,
                data.frame(var=names(mod$coefficients[2:length(mod$coefficients)]),
                           est=exp(mod$coefficients[2:length(mod$coefficients)]),
                           lci=exp(confint.default(mod)[2:length(mod$coefficients),1]),
                           uci=exp(confint.default(mod)[2:length(mod$coefficients),2])))
rm(mod)

mod <- glm(inse2010 ~ died2011, data=data,
           subset=data$statpop2010==1, family = binomial())

output <- rbind(output,
                data.frame(var=names(mod$coefficients[2:length(mod$coefficients)]),
                           est=exp(mod$coefficients[2:length(mod$coefficients)]),
                           lci=exp(confint.default(mod)[2:length(mod$coefficients),1]),
                           uci=exp(confint.default(mod)[2:length(mod$coefficients),2])))
rm(mod)

dimnames(output)[[1]] <- 1:length(dimnames(output)[[1]])

output
```

```
##                          var       est       lci       uci
## 1    r10_statpop_agecat520- 0.9343878 0.9159445 0.9532024
## 2    r10_statpop_agecat525- 1.0198941 1.0004681 1.0396972
## 3    r10_statpop_agecat530- 1.1174232 1.0965803 1.1386622
## 4    r10_statpop_agecat535- 1.1181712 1.0974791 1.1392534
## 5    r10_statpop_agecat540- 1.0879641 1.0682335 1.1080591
## 6    r10_statpop_agecat545- 1.0942136 1.0744830 1.1143065
## 7    r10_statpop_agecat550- 1.0879232 1.0677159 1.1085130
## 8    r10_statpop_agecat555- 1.1100281 1.0887962 1.1316740
## 9    r10_statpop_agecat560- 1.1118561 1.0903167 1.1338210
## 10   r10_statpop_agecat565- 1.1492376 1.1263416 1.1725991
## 11   r10_statpop_agecat570- 1.1457018 1.1211694 1.1707710
## 12   r10_statpop_agecat575- 1.1464542 1.1206745 1.1728270
## 13   r10_statpop_agecat580- 1.0983005 1.0710174 1.1262786
## 14   r10_statpop_agecat585- 0.9089838 0.8843204 0.9343351
## 15                   female 1.0366692 1.0292986 1.0440927
## 16           r10_natEU/EFTA 0.9441447 0.9345296 0.9538587
## 17      r10_natOther Europe 0.8092078 0.7945234 0.8241636
## 18       r10_natOther world 0.7877280 0.7702867 0.8055643
## 19         r10_civilMarried 1.0567839 1.0483939 1.0652410
```

```
## 20          r10_civilWidowed 0.9870434 0.9713627 1.0029773
## 21            r10_civilOther 1.0397864 1.0259945 1.0537636
## 22           r10_canton_seBE 1.0696812 1.0502078 1.0895156
## 23           r10_canton_seLU 2.0922211 2.0523785 2.1328372
## 24           r10_canton_seUR 1.1168060 1.0466629 1.1916499
## 25           r10_canton_seSZ 0.9824343 0.9482447 1.0178567
## 26           r10_canton_seOW 0.9347093 0.8714731 1.0025340
## 27           r10_canton_seNW 1.0135957 0.9518682 1.0793261
## 28           r10_canton_seGL 0.9340134 0.8737523 0.9984305
## 29           r10_canton_seZG 1.8909976 1.8340432 1.9497207
## 30           r10_canton_seFR 0.9635439 0.9374064 0.9904102
## 31           r10_canton_seSO 1.0237648 0.9960445 1.0522566
## 32           r10_canton_seBS 1.0569627 1.0252226 1.0896855
## 33           r10_canton_seBL 1.0584307 1.0309285 1.0866666
## 34           r10_canton_seSH 1.0249405 0.9788160 1.0732386
## 35           r10_canton_seAR 1.0621506 1.0055655 1.1219198
## 36           r10_canton_seAI 1.0022202 0.9046300 1.1103382
## 37           r10_canton_seSG 1.0070443 0.9851831 1.0293907
## 38           r10_canton_seGR 0.9775217 0.9481088 1.0078472
## 39           r10_canton_seAG 2.0235285 1.9891880 2.0584619
## 40           r10_canton_seTG 2.0055278 1.9615391 2.0505030
## 41           r10_canton_seTI 2.1157632 2.0742793 2.1580767
## 42           r10_canton_seVD 1.9770581 1.9445223 2.0101383
## 43           r10_canton_seVS 0.8973728 0.8737921 0.9215899
## 44           r10_canton_seNE 2.2612001 2.2066489 2.3170998
## 45           r10_canton_seGE 1.9366322 1.9004721 1.9734803
## 46           r10_canton_seJU 2.2511091 2.1728492 2.3321876
## 47        r10_canton_seBE-JU 2.3301389 2.2397066 2.4242225
## 48    r10_canton_seCity Bern 4.2435951 4.1511140 4.3381365
## 49 r10_canton_seCity Zurich 4.3109022 4.2408682 4.3820927
## 50                  died2011 0.6670748 0.6377409 0.6977579
```

Supplemental Table 1: CS weights 2010

```
datainse2010 <- data[data$inse2010==1, ]

datainse2010$female <- as.factor(datainse2010$female)
datainse2010$died2011 <- as.factor(datainse2010$died2011)

svo <- svydesign(id = ~SNCID, weights = ~weight2010, data = datainse2010)

round(coef(svytotal(~r10_statpop_agecat5, svo))/sum(datainse2010$weight2010)*100,1)
```

```
##  r10_statpop_agecat515-  r10_statpop_agecat520-  r10_statpop_agecat525-
##                     6.8                     7.3                     7.8
##  r10_statpop_agecat530-  r10_statpop_agecat535-  r10_statpop_agecat540-
```

```
##                      8.0                      8.4                      9.6
##  r10_statpop_agecat545-  r10_statpop_agecat550-  r10_statpop_agecat555-
##                      9.8                      8.6                      7.4
##  r10_statpop_agecat560-  r10_statpop_agecat565-  r10_statpop_agecat570-
##                      7.0                      6.0                      4.6
##  r10_statpop_agecat575-  r10_statpop_agecat580-  r10_statpop_agecat585-
##                      3.8                      2.8                      2.2
## r10_statpop_agecat5120-
##                      0.0
```

```
round(coef(svytotal(~female, svo))/sum(datainse2010$weight2010)*100,1)
```

```
## female0 female1
##    49.1    50.9
```

```
round(coef(svytotal(~r10_nat, svo))/sum(datainse2010$weight2010)*100,1)
```

```
##          r10_natCHE      r10_natEU/EFTA r10_natOther Europe
##                78.2                14.1                 4.8
##  r10_natOther world
##                 2.9
```

```
round(coef(svytotal(~r10_civil, svo))/sum(datainse2010$weight2010)*100,1)
```

```
##  r10_civilSingle r10_civilMarried r10_civilWidowed   r10_civilOther
##             32.6             52.6              5.7              9.1
```

```
round(coef(svytotal(~educ_agg2_2010, svo))/sum(datainse2010$weight2010)*100,1)
```

```
##    educ_agg2_2010Compulsory education or less
##                                          24.9
## educ_agg2_2010Upper secondary level education
##                                          47.9
##        educ_agg2_2010Tertiary level education
##                                          24.9
##                            educ_agg2_2010None
##                                           2.3
```

```
round(coef(svytotal(~employ_agg_2010, svo))/sum(datainse2010$weight2010)*100,1)
```

```
## employ_agg_2010Full-time employed employ_agg_2010Part-time employed
##                              44.4                              18.7
##         employ_agg_2010Unemployed    employ_agg_2010Inactive person
##                               3.0                              33.9
```

```
round(coef(svytotal(~religion2_2010, svo))/sum(datainse2010$weight2010)*100,1)
```

```
##      religion2_2010Protestant Churches
##                                   28.0
##    religion2_2010Roman Catholic Church
##                                   38.6
## religion2_2010No religious affiliation
```

```
##                                  20.1
##         religion2_2010Other/No response
##                                  13.3
```

Supplemental Table 1: IPW results

```
svo <- svydesign(id = ~SNCID, weights = ~ipw20102011, data = datainse2010)

round(coef(svytotal(~r10_statpop_agecat5, svo))/sum(datainse2010$ipw20102011)*100,1)
```

```
##  r10_statpop_agecat515-  r10_statpop_agecat520-  r10_statpop_agecat525-
##                     6.8                     7.5                     8.0
##  r10_statpop_agecat530-  r10_statpop_agecat535-  r10_statpop_agecat540-
##                     8.0                     8.4                     9.5
##  r10_statpop_agecat545-  r10_statpop_agecat550-  r10_statpop_agecat555-
##                     9.7                     8.4                     7.2
##  r10_statpop_agecat560-  r10_statpop_agecat565-  r10_statpop_agecat570-
##                     6.8                     5.9                     4.5
##  r10_statpop_agecat575-  r10_statpop_agecat580-  r10_statpop_agecat585-
##                     3.8                     2.9                     2.6
## r10_statpop_agecat5120-
##                     0.0
```

```
round(coef(svytotal(~female, svo))/sum(datainse2010$ipw20102011)*100,1)
```

```
## female0 female1
##    49.1    50.9
```

```
round(coef(svytotal(~r10_nat, svo))/sum(datainse2010$ipw20102011)*100,1)
```

```
##          r10_natCHE      r10_natEU/EFTA r10_natOther Europe
##                77.4                14.7                 4.7
##  r10_natOther world
##                 3.2
```

```
round(coef(svytotal(~r10_civil, svo))/sum(datainse2010$ipw20102011)*100,1)
```

```
##  r10_civilSingle r10_civilMarried r10_civilWidowed   r10_civilOther
##             33.2             51.7              6.0              9.1
```

```
round(coef(svytotal(~educ_agg2_2010, svo))/sum(datainse2010$ipw20102011)*100,1)
```

```
##    educ_agg2_2010Compulsory education or less
##                                          25.1
## educ_agg2_2010Upper secondary level education
##                                          47.7
##        educ_agg2_2010Tertiary level education
##                                          24.9
##                            educ_agg2_2010None
```

```
##                                                   2.3
```

```
round(coef(svytotal(~employ_agg_2010, svo))/sum(datainse2010$ipw20102011)*100,1)
```

```
## employ_agg_2010Full-time employed employ_agg_2010Part-time employed
##                              44.6                              18.3
##         employ_agg_2010Unemployed    employ_agg_2010Inactive person
##                               3.1                              34.0
```

```
round(coef(svytotal(~religion2_2010, svo))/sum(datainse2010$ipw20102011)*100,1)
```

```
##      religion2_2010Protestant Churches
##                                   27.9
##    religion2_2010Roman Catholic Church
##                                   38.6
## religion2_2010No religious affiliation
##                                   20.1
##        religion2_2010Other/No response
##                                   13.4
```

```
rm(datainse2010)
rm(svo)
```

Supplemental Table 2: STATPOP 2011

```
output <- c()

mod <- glm(inse2011 ~ r11_statpop_agecat5, data=data,
           subset=data$statpop2011==1, family = binomial())

output <- rbind(output,
                data.frame(var=names(mod$coefficients[2:length(mod$coefficients)]),
                           est=exp(mod$coefficients[2:length(mod$coefficients)]),
                           lci=exp(confint.default(mod)[2:length(mod$coefficients),1]),
                           uci=exp(confint.default(mod)[2:length(mod$coefficients),2])))
rm(mod)

mod <- glm(inse2011 ~ female, data=data,
           subset=data$statpop2011==1, family = binomial())

output <- rbind(output,
                data.frame(var=names(mod$coefficients[2:length(mod$coefficients)]),
                           est=exp(mod$coefficients[2:length(mod$coefficients)]),
                           lci=exp(confint.default(mod)[2:length(mod$coefficients),1]),
                           uci=exp(confint.default(mod)[2:length(mod$coefficients),2])))
rm(mod)
```

```r
mod <- glm(inse2011 ~ r11_nat, data=data,
           subset=data$statpop2011==1, family = binomial())

output <- rbind(output,
                data.frame(var=names(mod$coefficients[2:length(mod$coefficients)]),
                           est=exp(mod$coefficients[2:length(mod$coefficients)]),
                           lci=exp(confint.default(mod)[2:length(mod$coefficients),1]),
                           uci=exp(confint.default(mod)[2:length(mod$coefficients),2])))
rm(mod)

mod <- glm(inse2011 ~ r11_civil, data=data,
           subset=data$statpop2011==1, family = binomial())

output <- rbind(output,
                data.frame(var=names(mod$coefficients[2:length(mod$coefficients)]),
                           est=exp(mod$coefficients[2:length(mod$coefficients)]),
                           lci=exp(confint.default(mod)[2:length(mod$coefficients),1]),
                           uci=exp(confint.default(mod)[2:length(mod$coefficients),2])))
rm(mod)

mod <- glm(inse2011 ~ died2012, data=data,
           subset=data$statpop2011==1, family = binomial())

output <- rbind(output,
                data.frame(var=names(mod$coefficients[2:length(mod$coefficients)]),
                           est=exp(mod$coefficients[2:length(mod$coefficients)]),
                           lci=exp(confint.default(mod)[2:length(mod$coefficients),1]),
                           uci=exp(confint.default(mod)[2:length(mod$coefficients),2])))
rm(mod)

dimnames(output)[[1]] <- 1:length(dimnames(output)[[1]])

output
```

```
##                         var       est       lci       uci
## 1  r11_statpop_agecat520- 0.9393291 0.9189065 0.9602057
## 2  r11_statpop_agecat525- 0.9500729 0.9297676 0.9708215
## 3  r11_statpop_agecat530- 0.9969163 0.9758993 1.0183859
## 4  r11_statpop_agecat535- 1.0446549 1.0228299 1.0669457
## 5  r11_statpop_agecat540- 1.0508507 1.0293911 1.0727578
## 6  r11_statpop_agecat545- 1.0628637 1.0414064 1.0847631
## 7  r11_statpop_agecat550- 1.0905695 1.0681360 1.1134743
## 8  r11_statpop_agecat555- 1.0811647 1.0582207 1.1046062
## 9  r11_statpop_agecat560- 1.1095569 1.0857011 1.1339368
## 10 r11_statpop_agecat565- 1.1197506 1.0951631 1.1448901
## 11 r11_statpop_agecat570- 1.0950607 1.0692726 1.1214708
## 12 r11_statpop_agecat575- 1.1009202 1.0737100 1.1288200
```

```
## 13 r11_statpop_agecat580- 0.9857022 0.9584691 1.0137091
## 14 r11_statpop_agecat585- 0.7684505 0.7450102 0.7926283
## 15                 female 1.0287790 1.0210055 1.0366117
## 16          r11_natEU/EFTA 0.9135193 0.9036539 0.9234923
## 17    r11_natOther Europe 0.8226150 0.8067815 0.8387593
## 18     r11_natOther world 0.7716353 0.7536318 0.7900689
## 19       r11_civilMarried 1.1449788 1.1351935 1.1548484
## 20       r11_civilWidowed 1.0077128 0.9904354 1.0252915
## 21          r11_civilOther 1.0680270 1.0529376 1.0833326
## 22                died2012 0.5908120 0.5620408 0.6210561
```

Supplemental Table 2: STATPOP 2012

```
output <- c()

mod <- glm(inse2012 ~ r12_statpop_agecat5, data=data,
           subset=data$statpop2012==1, family = binomial())

output <- rbind(output,
                data.frame(var=names(mod$coefficients[2:length(mod$coefficients)]),
                           est=exp(mod$coefficients[2:length(mod$coefficients)]),
                           lci=exp(confint.default(mod)[2:length(mod$coefficients),1]),
                           uci=exp(confint.default(mod)[2:length(mod$coefficients),2])))
rm(mod)

mod <- glm(inse2012 ~ female, data=data,
           subset=data$statpop2012==1, family = binomial())

output <- rbind(output,
                data.frame(var=names(mod$coefficients[2:length(mod$coefficients)]),
                           est=exp(mod$coefficients[2:length(mod$coefficients)]),
                           lci=exp(confint.default(mod)[2:length(mod$coefficients),1]),
                           uci=exp(confint.default(mod)[2:length(mod$coefficients),2])))
rm(mod)

mod <- glm(inse2012 ~ r12_nat, data=data,
           subset=data$statpop2012==1, family = binomial())

output <- rbind(output,
                data.frame(var=names(mod$coefficients[2:length(mod$coefficients)]),
                           est=exp(mod$coefficients[2:length(mod$coefficients)]),
                           lci=exp(confint.default(mod)[2:length(mod$coefficients),1]),
                           uci=exp(confint.default(mod)[2:length(mod$coefficients),2])))
rm(mod)

mod <- glm(inse2012 ~ r12_civil, data=data,
```

```r
           subset=data$statpop2012==1, family = binomial())

output <- rbind(output,
                data.frame(var=names(mod$coefficients[2:length(mod$coefficients)]),
                           est=exp(mod$coefficients[2:length(mod$coefficients)]),
                           lci=exp(confint.default(mod)[2:length(mod$coefficients),1]),
                           uci=exp(confint.default(mod)[2:length(mod$coefficients),2])))
rm(mod)

mod <- glm(inse2012 ~ died2013, data=data,
           subset=data$statpop2012==1, family = binomial())

output <- rbind(output,
                data.frame(var=names(mod$coefficients[2:length(mod$coefficients)]),
                           est=exp(mod$coefficients[2:length(mod$coefficients)]),
                           lci=exp(confint.default(mod)[2:length(mod$coefficients),1]),
                           uci=exp(confint.default(mod)[2:length(mod$coefficients),2])))
rm(mod)

dimnames(output)[[1]] <- 1:length(dimnames(output)[[1]])

output
```

```
##                        var       est       lci       uci
## 1  r12_statpop_agecat520- 0.9224585 0.9007862 0.9446521
## 2  r12_statpop_agecat525- 0.9228656 0.9014417 0.9447986
## 3  r12_statpop_agecat530- 0.9732619 0.9509980 0.9960470
## 4  r12_statpop_agecat535- 1.0174538 0.9942696 1.0411786
## 5  r12_statpop_agecat540- 1.0528489 1.0293427 1.0768918
## 6  r12_statpop_agecat545- 1.0598791 1.0365059 1.0837794
## 7  r12_statpop_agecat550- 1.0615310 1.0377561 1.0858507
## 8  r12_statpop_agecat555- 1.0839221 1.0591005 1.1093253
## 9  r12_statpop_agecat560- 1.0784890 1.0532950 1.1042855
## 10 r12_statpop_agecat565- 1.1147278 1.0884213 1.1416701
## 11 r12_statpop_agecat570- 1.0888933 1.0617482 1.1167325
## 12 r12_statpop_agecat575- 1.0721109 1.0438889 1.1010959
## 13 r12_statpop_agecat580- 1.0016173 0.9729019 1.0311802
## 14 r12_statpop_agecat585- 0.7549885 0.7311355 0.7796197
## 15                  female 1.0377037 1.0298595 1.0456076
## 16          r12_natEU/EFTA 0.9073941 0.8977022 0.9171907
## 17     r12_natOther Europe 0.8251817 0.8092251 0.8414529
## 18      r12_natOther world 0.7588765 0.7412981 0.7768719
## 19        r12_civilMarried 1.1538323 1.1439167 1.1638338
## 20        r12_civilWidowed 0.9971369 0.9798967 1.0146804
## 21          r12_civilOther 1.0560125 1.0411388 1.0710987
## 22                died2013 0.5734078 0.5453482 0.6029111
```

Supplemental Table 2: STATPOP 2013

```
output <- c()

mod <- glm(inse2013 ~ r13_statpop_agecat5, data=data,
           subset=data$statpop2013==1, family = binomial())

output <- rbind(output,
                data.frame(var=names(mod$coefficients[2:length(mod$coefficients)]),
                           est=exp(mod$coefficients[2:length(mod$coefficients)]),
                           lci=exp(confint.default(mod)[2:length(mod$coefficients),1]),
                           uci=exp(confint.default(mod)[2:length(mod$coefficients),2])))
rm(mod)

mod <- glm(inse2013 ~ female, data=data,
           subset=data$statpop2013==1, family = binomial())

output <- rbind(output,
                data.frame(var=names(mod$coefficients[2:length(mod$coefficients)]),
                           est=exp(mod$coefficients[2:length(mod$coefficients)]),
                           lci=exp(confint.default(mod)[2:length(mod$coefficients),1]),
                           uci=exp(confint.default(mod)[2:length(mod$coefficients),2])))
rm(mod)

mod <- glm(inse2013 ~ r13_nat, data=data,
           subset=data$statpop2013==1, family = binomial())

output <- rbind(output,
                data.frame(var=names(mod$coefficients[2:length(mod$coefficients)]),
                           est=exp(mod$coefficients[2:length(mod$coefficients)]),
                           lci=exp(confint.default(mod)[2:length(mod$coefficients),1]),
                           uci=exp(confint.default(mod)[2:length(mod$coefficients),2])))
rm(mod)

mod <- glm(inse2013 ~ r13_civil, data=data,
           subset=data$statpop2013==1, family = binomial())

output <- rbind(output,
                data.frame(var=names(mod$coefficients[2:length(mod$coefficients)]),
                           est=exp(mod$coefficients[2:length(mod$coefficients)]),
                           lci=exp(confint.default(mod)[2:length(mod$coefficients),1]),
                           uci=exp(confint.default(mod)[2:length(mod$coefficients),2])))
rm(mod)

mod <- glm(inse2013 ~ died2014, data=data,
           subset=data$statpop2013==1, family = binomial())
```

```r
output <- rbind(output,
                data.frame(var=names(mod$coefficients[2:length(mod$coefficients)]),
                           est=exp(mod$coefficients[2:length(mod$coefficients)]),
                           lci=exp(confint.default(mod)[2:length(mod$coefficients),1]),
                           uci=exp(confint.default(mod)[2:length(mod$coefficients),2])))
rm(mod)

dimnames(output)[[1]] <- 1:length(dimnames(output)[[1]])

output
```

```
##                        var       est       lci       uci
## 1  r13_statpop_agecat520- 0.9568284 0.9308014 0.9835830
## 2  r13_statpop_agecat525- 0.9652129 0.9392870 0.9918544
## 3  r13_statpop_agecat530- 1.0352376 1.0077843 1.0634388
## 4  r13_statpop_agecat535- 1.0654670 1.0372101 1.0944937
## 5  r13_statpop_agecat540- 1.0791844 1.0508493 1.1082836
## 6  r13_statpop_agecat545- 1.0910810 1.0627581 1.1201587
## 7  r13_statpop_agecat550- 1.0951459 1.0665046 1.1245564
## 8  r13_statpop_agecat555- 1.1123432 1.0827370 1.1427590
## 9  r13_statpop_agecat560- 1.1293150 1.0987722 1.1607069
## 10 r13_statpop_agecat565- 1.1457762 1.1145402 1.1778876
## 11 r13_statpop_agecat570- 1.1335206 1.1014982 1.1664740
## 12 r13_statpop_agecat575- 1.1135046 1.0805089 1.1475080
## 13 r13_statpop_agecat580- 1.0178578 0.9853934 1.0513916
## 14 r13_statpop_agecat585- 0.7815379 0.7546769 0.8093551
## 15                  female 1.0323318 1.0244287 1.0402958
## 16          r13_natEU/EFTA 0.9169011 0.9071579 0.9267490
## 17     r13_natOther Europe 0.8133172 0.7971975 0.8297628
## 18      r13_natOther world 0.7893027 0.7712260 0.8078031
## 19        r13_civilMarried 1.1407438 1.1307718 1.1508038
## 20        r13_civilWidowed 1.0007116 0.9832151 1.0185195
## 21          r13_civilOther 1.0637598 1.0487884 1.0789448
## 22                died2014 0.5712650 0.5427232 0.6013079
```

Supplemental Table 3: 2010

```r
# Construct month of date of death
data$dod_month <- as.numeric(substring(data$dod, 6, 7))

# STATPOP
table(data$dod_month[data$statpop2010==1 & data$died2011==1])
```

```
##
##    1    2    3    4    5    6    7    8    9   10   11   12
## 5732 4994 5511 5019 4975 4731 4942 5032 4807 5186 5087 5523
```

```r
round(table(data$dod_month[data$statpop2010==1 & data$died2011==1])
/sum(data$statpop2010==1 & data$died2011==1, na.rm=T)*100,1)
```

```
##
##   1   2   3   4   5   6   7   8   9  10  11  12
## 9.3 8.1 9.0 8.2 8.1 7.7 8.0 8.2 7.8 8.4 8.3 9.0
```

```r
# SE
table(data$dod_month[data$inse2010==1 & data$died2011==1])
```

```
##
##   1   2   3   4   5   6   7   8   9  10  11  12
##  95 108 166 164 147 162 161 182 191 199 191 205
```

```r
round(table(data$dod_month[data$inse2010==1 & data$died2011==1])
/sum(data$inse2010==1 & data$died2011==1, na.rm=T)*100,1)
```

```
##
##    1    2    3    4    5    6    7    8    9   10   11   12
##  4.8  5.5  8.4  8.3  7.5  8.2  8.2  9.2  9.7 10.1  9.7 10.4
```

Supplemental Table 3: 2011

```r
# STATPOP
table(data$dod_month[data$statpop2011==1 & data$died2012==1])
```

```
##
##    1    2    3    4    5    6    7    8    9   10   11   12
## 5827 5796 5855 5413 5017 4799 4863 4936 4914 5327 5199 5658
```

```r
round(table(data$dod_month[data$statpop2011==1 & data$died2012==1])
/sum(data$statpop2011==1 & data$died2012==1, na.rm=T)*100,1)
```

```
##
##   1   2   3   4   5   6   7   8   9  10  11  12
## 9.2 9.1 9.2 8.5 7.9 7.5 7.6 7.8 7.7 8.4 8.2 8.9
```

```r
# SE
table(data$dod_month[data$inse2011==1 & data$died2012==1])
```

```
##
##   1   2   3   4   5   6   7   8   9  10  11  12
##  67 101 128 132 128 124 141 131 153 153 156 176
```

```r
round(table(data$dod_month[data$inse2011==1 & data$died2012==1])
/sum(data$inse2011==1 & data$died2012==1, na.rm=T)*100,1)
```

```
##
##    1    2    3    4    5    6    7    8    9   10   11   12
##  4.2  6.4  8.1  8.3  8.1  7.8  8.9  8.2  9.6  9.6  9.8 11.1
```

Supplemental Table 3: 2012

```
# STATPOP
table(data$dod_month[data$statpop2012==1 & data$died2013==1])
```

```
##
##    1    2    3    4    5    6    7    8    9   10   11   12
## 6073 5871 6204 5421 5000 4927 5160 5052 4965 5215 5182 5712
```

```
round(table(data$dod_month[data$statpop2012==1 & data$died2013==1])
/sum(data$statpop2012==1 & data$died2013==1, na.rm=T)*100,1)
```

```
##
##   1   2   3   4   5   6   7   8   9  10  11  12
## 9.4 9.1 9.6 8.4 7.7 7.6 8.0 7.8 7.7 8.1 8.0 8.8
```

```
# SE
table(data$dod_month[data$inse2012==1 & data$died2013==1])
```

```
##
##   1   2   3   4   5   6   7   8   9  10  11  12
##  50 104 145 110 135 121 147 146 154 150 160 151
```

```
round(table(data$dod_month[data$inse2012==1 & data$died2013==1])
/sum(data$inse2012==1 & data$died2013==1, na.rm=T)*100,1)
```

```
##
##    1    2    3    4    5    6    7    8    9   10   11   12
##  3.2  6.6  9.2  7.0  8.6  7.7  9.3  9.3  9.8  9.5 10.2  9.6
```

Supplemental Table 3: 2013

```
# STATPOP
table(data$dod_month[data$statpop2013==1 & data$died2014==1])
```

```
##
##    1    2    3    4    5    6    7    8    9   10   11   12
## 5762 5351 5700 5136 5204 4844 5152 4981 4953 5404 5582 5951
```

```
round(table(data$dod_month[data$statpop2013==1 & data$died2014==1])
/sum(data$statpop2013==1 & data$died2014==1, na.rm=T)*100,1)
```

```
##
##   1   2   3   4   5   6   7   8   9  10  11  12
## 9.0 8.4 8.9 8.0 8.1 7.6 8.0 7.8 7.7 8.4 8.7 9.3
```

```
# SE
table(data$dod_month[data$inse2013==1 & data$died2014==1])
```

```
##
```

```
##   1   2   3   4   5   6   7   8   9  10  11  12
##  34  86 121 135 143 104 139 143 123 157 159 162
```

```
round(table(data$dod_month[data$inse2013==1 & data$died2014==1])
/sum(data$inse2013==1 & data$died2014==1, na.rm=T)*100,1)
```

```
##
##    1    2    3    4    5    6    7    8    9   10   11   12
##  2.3  5.7  8.0  9.0  9.5  6.9  9.2  9.5  8.2 10.4 10.6 10.8
```

Figure 2 and Supplemental Figure 1

```
data$r10_statpop_agecat5 <- as.numeric(data$r10_statpop_agecat5)-1

output <- c()

data$fupt2011 <- (data$statpop2010_enddate2011-data$statpop2010_startdate)/365.25
data$fupt2012 <- (data$statpop2010_enddate2012-data$statpop2010_startdate)/365.25
data$fupt2013 <- (data$statpop2010_enddate2013-data$statpop2010_startdate)/365.25
data$fupt2014 <- (data$statpop2010_enddate2014-data$statpop2010_startdate)/365.25

### Mortality rates of STATPOP population, by gender, with deaths up to year 2014

type <- 1

year <- 2011
sex <- 1

mod <- glm(died2011 ~ factor(r10_statpop_agecat5)-1, data=data,
           offset=log(fupt2011), subset=female==sex & statpop2010==1, family=poisson())

output <- rbind(output, data.frame(agecat=as.numeric(mod$xlevels[[1]]),
                          female=sex, rate=exp(mod$coeff), type=type, yeardod=year))

sex <- 0

mod <- glm(died2011 ~ factor(r10_statpop_agecat5)-1, data=data,
           offset=log(fupt2011), subset=female==sex & statpop2010==1, family=poisson())

output <- rbind(output, data.frame(agecat=as.numeric(mod$xlevels[[1]]),
                          female=sex, rate=exp(mod$coeff), type=type, yeardod=year))


year <- 2012
sex <- 1
```

```
mod <- glm(died2012 ~ factor(r10_statpop_agecat5)-1, data=data,
           offset=log(fupt2012), subset=female==sex & statpop2010==1, family=poisson())

output <- rbind(output, data.frame(agecat=as.numeric(mod$xlevels[[1]]),
                                   female=sex, rate=exp(mod$coeff), type=type, yeardod=year))

sex <- 0

mod <- glm(died2012 ~ factor(r10_statpop_agecat5)-1, data=data,
           offset=log(fupt2012), subset=female==sex & statpop2010==1, family=poisson())

output <- rbind(output, data.frame(agecat=as.numeric(mod$xlevels[[1]]),
                                   female=sex, rate=exp(mod$coeff), type=type, yeardod=year))


year <- 2013
sex <- 1

mod <- glm(died2013 ~ factor(r10_statpop_agecat5)-1, data=data,
           offset=log(fupt2013), subset=female==sex & statpop2010==1, family=poisson())

output <- rbind(output, data.frame(agecat=as.numeric(mod$xlevels[[1]]),
                                   female=sex, rate=exp(mod$coeff), type=type, yeardod=year))

sex <- 0

mod <- glm(died2013 ~ factor(r10_statpop_agecat5)-1, data=data,
           offset=log(fupt2013), subset=female==sex & statpop2010==1, family=poisson())

output <- rbind(output, data.frame(agecat=as.numeric(mod$xlevels[[1]]),
                                   female=sex, rate=exp(mod$coeff), type=type, yeardod=year))


year <- 2014
sex <- 1

mod <- glm(died2014 ~ factor(r10_statpop_agecat5)-1, data=data,
           offset=log(fupt2014), subset=female==sex & statpop2010==1, family=poisson())

output <- rbind(output, data.frame(agecat=as.numeric(mod$xlevels[[1]]),
                                   female=sex, rate=exp(mod$coeff), type=type, yeardod=year))

sex <- 0

mod <- glm(died2014 ~ factor(r10_statpop_agecat5)-1, data=data,
           offset=log(fupt2014), subset=female==sex & statpop2010==1, family=poisson())
```

```
output <- rbind(output, data.frame(agecat=as.numeric(mod$xlevels[[1]]),
                          female=sex, rate=exp(mod$coeff), type=type, yeardod=year))


### Mortality rates of SE population, by gender, with deaths up to year 2014
### using IP weights (type=2)

type <- 2

year <- 2011
sex <- 1

mod <- glm(died2011 ~ factor(r10_statpop_agecat5)-1, data=data, weights=ipw20102011,
           offset=log(fupt2011), subset=female==sex & inse2010==1, family=poisson())

output <- rbind(output, data.frame(agecat=as.numeric(mod$xlevels[[1]]),
                          female=sex, rate=exp(mod$coeff), type=type, yeardod=year))

sex <- 0

mod <- glm(died2011 ~ factor(r10_statpop_agecat5)-1, data=data, weights=ipw20102011,
           offset=log(fupt2011), subset=female==sex & inse2010==1, family=poisson())

output <- rbind(output, data.frame(agecat=as.numeric(mod$xlevels[[1]]),
                          female=sex, rate=exp(mod$coeff), type=type, yeardod=year))


year <- 2012
sex <- 1

mod <- glm(died2012 ~ factor(r10_statpop_agecat5)-1, data=data, weights=ipw20102012,
           offset=log(fupt2012), subset=female==sex & inse2010==1, family=poisson())

output <- rbind(output, data.frame(agecat=as.numeric(mod$xlevels[[1]]),
                          female=sex, rate=exp(mod$coeff), type=type, yeardod=year))

sex <- 0

mod <- glm(died2012 ~ factor(r10_statpop_agecat5)-1, data=data, weights=ipw20102012,
           offset=log(fupt2012), subset=female==sex & inse2010==1, family=poisson())

output <- rbind(output, data.frame(agecat=as.numeric(mod$xlevels[[1]]),
                          female=sex, rate=exp(mod$coeff), type=type, yeardod=year))


year <- 2013
sex <- 1
```

```r
mod <- glm(died2013 ~ factor(r10_statpop_agecat5)-1, data=data, weights=ipw20102013,
           offset=log(fupt2013), subset=female==sex & inse2010==1, family=poisson())

output <- rbind(output, data.frame(agecat=as.numeric(mod$xlevels[[1]]),
                          female=sex, rate=exp(mod$coeff), type=type, yeardod=year))

sex <- 0

mod <- glm(died2013 ~ factor(r10_statpop_agecat5)-1, data=data, weights=ipw20102013,
           offset=log(fupt2013), subset=female==sex & inse2010==1, family=poisson())

output <- rbind(output, data.frame(agecat=as.numeric(mod$xlevels[[1]]),
                          female=sex, rate=exp(mod$coeff), type=type, yeardod=year))


year <- 2014
sex <- 1

mod <- glm(died2014 ~ factor(r10_statpop_agecat5)-1, data=data, weights=ipw20102014,
           offset=log(fupt2014), subset=female==sex & inse2010==1, family=poisson())

output <- rbind(output, data.frame(agecat=as.numeric(mod$xlevels[[1]]),
                          female=sex, rate=exp(mod$coeff), type=type, yeardod=year))

sex <- 0

mod <- glm(died2014 ~ factor(r10_statpop_agecat5)-1, data=data, weights=ipw20102014,
           offset=log(fupt2014), subset=female==sex & inse2010==1, family=poisson())

output <- rbind(output, data.frame(agecat=as.numeric(mod$xlevels[[1]]),
                          female=sex, rate=exp(mod$coeff), type=type, yeardod=year))


### Mortality rates of SE population, by gender, with deaths up to year 2014
### using CS weights 2010 (type=3)

type <- 3

year <- 2011
sex <- 1

mod <- glm(died2011 ~ factor(r10_statpop_agecat5)-1, data=data, weights=weight2010,
           offset=log(fupt2011), subset=female==sex & inse2010==1, family=poisson())

output <- rbind(output, data.frame(agecat=as.numeric(mod$xlevels[[1]]),
                          female=sex, rate=exp(mod$coeff), type=type, yeardod=year))
```

```
sex <- 0

mod <- glm(died2011 ~ factor(r10_statpop_agecat5)-1, data=data, weights=weight2010,
           offset=log(fupt2011), subset=female==sex & inse2010==1, family=poisson())

output <- rbind(output, data.frame(agecat=as.numeric(mod$xlevels[[1]]),
                          female=sex, rate=exp(mod$coeff), type=type, yeardod=year))


year <- 2012
sex <- 1

mod <- glm(died2012 ~ factor(r10_statpop_agecat5)-1, data=data, weights=weight2010,
           offset=log(fupt2012), subset=female==sex & inse2010==1, family=poisson())

output <- rbind(output, data.frame(agecat=as.numeric(mod$xlevels[[1]]),
                          female=sex, rate=exp(mod$coeff), type=type, yeardod=year))

sex <- 0

mod <- glm(died2012 ~ factor(r10_statpop_agecat5)-1, data=data, weights=weight2010,
           offset=log(fupt2012), subset=female==sex & inse2010==1, family=poisson())

output <- rbind(output, data.frame(agecat=as.numeric(mod$xlevels[[1]]),
                          female=sex, rate=exp(mod$coeff), type=type, yeardod=year))


year <- 2013
sex <- 1

mod <- glm(died2013 ~ factor(r10_statpop_agecat5)-1, data=data, weights=weight2010,
           offset=log(fupt2013), subset=female==sex & inse2010==1, family=poisson())

output <- rbind(output, data.frame(agecat=as.numeric(mod$xlevels[[1]]),
                          female=sex, rate=exp(mod$coeff), type=type, yeardod=year))

sex <- 0

mod <- glm(died2013 ~ factor(r10_statpop_agecat5)-1, data=data, weights=weight2010,
           offset=log(fupt2013), subset=female==sex & inse2010==1, family=poisson())

output <- rbind(output, data.frame(agecat=as.numeric(mod$xlevels[[1]]),
                          female=sex, rate=exp(mod$coeff), type=type, yeardod=year))


year <- 2014
sex <- 1
```

```
mod <- glm(died2014 ~ factor(r10_statpop_agecat5)-1, data=data, weights=weight2010,
           offset=log(fupt2014), subset=female==sex & inse2010==1, family=poisson())

output <- rbind(output, data.frame(agecat=as.numeric(mod$xlevels[[1]]),
                          female=sex, rate=exp(mod$coeff), type=type, yeardod=year))

sex <- 0

mod <- glm(died2014 ~ factor(r10_statpop_agecat5)-1, data=data, weights=weight2010,
           offset=log(fupt2014), subset=female==sex & inse2010==1, family=poisson())

output <- rbind(output, data.frame(agecat=as.numeric(mod$xlevels[[1]]),
                          female=sex, rate=exp(mod$coeff), type=type, yeardod=year))


### Mortality rates of SE population, by gender, with deaths up to year 2014
### using no weights (type=4)

type <- 4

year <- 2011
sex <- 1

mod <- glm(died2011 ~ factor(r10_statpop_agecat5)-1, data=data,
           offset=log(fupt2011), subset=female==sex & inse2010==1, family=poisson())

output <- rbind(output, data.frame(agecat=as.numeric(mod$xlevels[[1]]),
                          female=sex, rate=exp(mod$coeff), type=type, yeardod=year))

sex <- 0

mod <- glm(died2011 ~ factor(r10_statpop_agecat5)-1, data=data,
           offset=log(fupt2011), subset=female==sex & inse2010==1, family=poisson())

output <- rbind(output, data.frame(agecat=as.numeric(mod$xlevels[[1]]),
                          female=sex, rate=exp(mod$coeff), type=type, yeardod=year))


year <- 2012
sex <- 1

mod <- glm(died2012 ~ factor(r10_statpop_agecat5)-1, data=data,
           offset=log(fupt2012), subset=female==sex & inse2010==1, family=poisson())

output <- rbind(output, data.frame(agecat=as.numeric(mod$xlevels[[1]]),
                          female=sex, rate=exp(mod$coeff), type=type, yeardod=year))
```

```
sex <- 0

mod <- glm(died2012 ~ factor(r10_statpop_agecat5)-1, data=data,
           offset=log(fupt2012), subset=female==sex & inse2010==1, family=poisson())

output <- rbind(output, data.frame(agecat=as.numeric(mod$xlevels[[1]]),
                          female=sex, rate=exp(mod$coeff), type=type, yeardod=year))


year <- 2013
sex <- 1

mod <- glm(died2013 ~ factor(r10_statpop_agecat5)-1, data=data,
           offset=log(fupt2013), subset=female==sex & inse2010==1, family=poisson())

output <- rbind(output, data.frame(agecat=as.numeric(mod$xlevels[[1]]),
                          female=sex, rate=exp(mod$coeff), type=type, yeardod=year))

sex <- 0

mod <- glm(died2013 ~ factor(r10_statpop_agecat5)-1, data=data,
           offset=log(fupt2013), subset=female==sex & inse2010==1, family=poisson())

output <- rbind(output, data.frame(agecat=as.numeric(mod$xlevels[[1]]),
                          female=sex, rate=exp(mod$coeff), type=type, yeardod=year))


year <- 2014
sex <- 1

mod <- glm(died2014 ~ factor(r10_statpop_agecat5)-1, data=data,
           offset=log(fupt2014), subset=female==sex & inse2010==1, family=poisson())

output <- rbind(output, data.frame(agecat=as.numeric(mod$xlevels[[1]]),
                          female=sex, rate=exp(mod$coeff), type=type, yeardod=year))

sex <- 0

mod <- glm(died2014 ~ factor(r10_statpop_agecat5)-1, data=data,
           offset=log(fupt2014), subset=female==sex & inse2010==1, family=poisson())

output <- rbind(output, data.frame(agecat=as.numeric(mod$xlevels[[1]]),
                          female=sex, rate=exp(mod$coeff), type=type, yeardod=year))

output$agecat <- factor(output$agecat, levels=0:14,
                          labels=c("[15,20)", "[20,25)", "[25,30)", "[30,35)",
                                   "[35,40)", "[40,45)", "[45,50)", "[50,55)",
```

```
                                    "[55,60)", "[60,65)", "[65,70)", "[70,75)" ,
                                    "[75,80)", "[80,85)", ">=85" ))




output$female <- factor(output$female, levels=0:1, labels=c("Men", "Women"))
output$type <- factor(output$type, levels=1:4, labels=c("STATPOP 2010",
                            "SE 2010 (IPW)", "SE 2010 (CSW)", "SE 2010 (unweighted)"))

factor <- 100000
output$rate <- output$rate*factor
dimnames(output)[[1]] <- 1:length(dimnames(output)[[1]])
```

```
### Show results
output
```

```
##       agecat female         rate                 type yeardod
## 1    [15,20)  Women    15.380260         STATPOP 2010    2011
## 2    [20,25)  Women    23.692963         STATPOP 2010    2011
## 3    [25,30)  Women    21.999110         STATPOP 2010    2011
## 4    [30,35)  Women    35.062308         STATPOP 2010    2011
## 5    [35,40)  Women    41.592267         STATPOP 2010    2011
## 6    [40,45)  Women    78.256865         STATPOP 2010    2011
## 7    [45,50)  Women   130.161288         STATPOP 2010    2011
## 8    [50,55)  Women   198.162944         STATPOP 2010    2011
## 9    [55,60)  Women   310.313967         STATPOP 2010    2011
## 10   [60,65)  Women   498.682109         STATPOP 2010    2011
## 11   [65,70)  Women   784.426453         STATPOP 2010    2011
## 12   [70,75)  Women  1250.939370         STATPOP 2010    2011
## 13   [75,80)  Women  2287.394228         STATPOP 2010    2011
## 14   [80,85)  Women  4645.092145         STATPOP 2010    2011
## 15      >=85  Women 13669.350375         STATPOP 2010    2011
## 16   [15,20)    Men    39.206348         STATPOP 2010    2011
## 17   [20,25)    Men    54.172354         STATPOP 2010    2011
## 18   [25,30)    Men    44.584240         STATPOP 2010    2011
## 19   [30,35)    Men    56.967733         STATPOP 2010    2011
## 20   [35,40)    Men    75.507154         STATPOP 2010    2011
## 21   [40,45)    Men   129.286238         STATPOP 2010    2011
## 22   [45,50)    Men   202.047237         STATPOP 2010    2011
## 23   [50,55)    Men   338.889367         STATPOP 2010    2011
## 24   [55,60)    Men   532.989780         STATPOP 2010    2011
## 25   [60,65)    Men   864.083681         STATPOP 2010    2011
## 26   [65,70)    Men  1392.860068         STATPOP 2010    2011
## 27   [70,75)    Men  2208.871129         STATPOP 2010    2011
## 28   [75,80)    Men  3936.307593         STATPOP 2010    2011
## 29   [80,85)    Men  7272.737379         STATPOP 2010    2011
## 30      >=85    Men 16596.803811         STATPOP 2010    2011
```

```
## 31  [15,20)  Women    14.908664         STATPOP 2010    2012
## 32  [20,25)  Women    19.583231         STATPOP 2010    2012
## 33  [25,30)  Women    24.055229         STATPOP 2010    2012
## 34  [30,35)  Women    32.971111         STATPOP 2010    2012
## 35  [35,40)  Women    41.364691         STATPOP 2010    2012
## 36  [40,45)  Women    79.605158         STATPOP 2010    2012
## 37  [45,50)  Women   133.346162         STATPOP 2010    2012
## 38  [50,55)  Women   212.818923         STATPOP 2010    2012
## 39  [55,60)  Women   334.964827         STATPOP 2010    2012
## 40  [60,65)  Women   511.988445         STATPOP 2010    2012
## 41  [65,70)  Women   815.207700         STATPOP 2010    2012
## 42  [70,75)  Women  1313.487696         STATPOP 2010    2012
## 43  [75,80)  Women  2438.208086         STATPOP 2010    2012
## 44  [80,85)  Women  5107.377192         STATPOP 2010    2012
## 45     >=85  Women 14514.484078         STATPOP 2010    2012
## 46  [15,20)    Men    42.388859         STATPOP 2010    2012
## 47  [20,25)    Men    47.594771         STATPOP 2010    2012
## 48  [25,30)    Men    44.533236         STATPOP 2010    2012
## 49  [30,35)    Men    56.724017         STATPOP 2010    2012
## 50  [35,40)    Men    81.075903         STATPOP 2010    2012
## 51  [40,45)    Men   130.596935         STATPOP 2010    2012
## 52  [45,50)    Men   204.085855         STATPOP 2010    2012
## 53  [50,55)    Men   353.823037         STATPOP 2010    2012
## 54  [55,60)    Men   557.651319         STATPOP 2010    2012
## 55  [60,65)    Men   919.518490         STATPOP 2010    2012
## 56  [65,70)    Men  1442.527010         STATPOP 2010    2012
## 57  [70,75)    Men  2271.858128         STATPOP 2010    2012
## 58  [75,80)    Men  4113.722387         STATPOP 2010    2012
## 59  [80,85)    Men  7637.382563         STATPOP 2010    2012
## 60     >=85    Men 17424.266732         STATPOP 2010    2012
## 61  [15,20)  Women    14.916432         STATPOP 2010    2013
## 62  [20,25)  Women    19.049650         STATPOP 2010    2013
## 63  [25,30)  Women    22.489631         STATPOP 2010    2013
## 64  [30,35)  Women    33.178134         STATPOP 2010    2013
## 65  [35,40)  Women    46.589488         STATPOP 2010    2013
## 66  [40,45)  Women    80.783676         STATPOP 2010    2013
## 67  [45,50)  Women   136.777318         STATPOP 2010    2013
## 68  [50,55)  Women   219.382220         STATPOP 2010    2013
## 69  [55,60)  Women   352.344082         STATPOP 2010    2013
## 70  [60,65)  Women   530.243766         STATPOP 2010    2013
## 71  [65,70)  Women   848.548012         STATPOP 2010    2013
## 72  [70,75)  Women  1384.240588         STATPOP 2010    2013
## 73  [75,80)  Women  2611.263290         STATPOP 2010    2013
## 74  [80,85)  Women  5500.885598         STATPOP 2010    2013
## 75     >=85  Women 15179.297934         STATPOP 2010    2013
## 76  [15,20)    Men    42.488941         STATPOP 2010    2013
## 77  [20,25)    Men    47.429249         STATPOP 2010    2013
## 78  [25,30)    Men    45.790949         STATPOP 2010    2013
```

```
## 79  [30,35)    Men    57.191740         STATPOP 2010    2013
## 80  [35,40)    Men    84.558627         STATPOP 2010    2013
## 81  [40,45)    Men   135.994060         STATPOP 2010    2013
## 82  [45,50)    Men   210.433699         STATPOP 2010    2013
## 83  [50,55)    Men   368.793606         STATPOP 2010    2013
## 84  [55,60)    Men   591.144283         STATPOP 2010    2013
## 85  [60,65)    Men   943.956209         STATPOP 2010    2013
## 86  [65,70)    Men  1485.995844         STATPOP 2010    2013
## 87  [70,75)    Men  2392.321088         STATPOP 2010    2013
## 88  [75,80)    Men  4309.917821         STATPOP 2010    2013
## 89  [80,85)    Men  8126.829424         STATPOP 2010    2013
## 90     >=85    Men 18106.733018         STATPOP 2010    2013
## 91  [15,20)  Women    14.581768         STATPOP 2010    2014
## 92  [20,25)  Women    18.885512         STATPOP 2010    2014
## 93  [25,30)  Women    23.792770         STATPOP 2010    2014
## 94  [30,35)  Women    32.911359         STATPOP 2010    2014
## 95  [35,40)  Women    48.315198         STATPOP 2010    2014
## 96  [40,45)  Women    83.054871         STATPOP 2010    2014
## 97  [45,50)  Women   138.388574         STATPOP 2010    2014
## 98  [50,55)  Women   228.654148         STATPOP 2010    2014
## 99  [55,60)  Women   357.878563         STATPOP 2010    2014
## 100 [60,65)  Women   549.741069         STATPOP 2010    2014
## 101 [65,70)  Women   883.563996         STATPOP 2010    2014
## 102 [70,75)  Women  1453.069385         STATPOP 2010    2014
## 103 [75,80)  Women  2778.273182         STATPOP 2010    2014
## 104 [80,85)  Women  5839.386519         STATPOP 2010    2014
## 105    >=85  Women 15620.656445         STATPOP 2010    2014
## 106 [15,20)    Men    41.035243         STATPOP 2010    2014
## 107 [20,25)    Men    47.253309         STATPOP 2010    2014
## 108 [25,30)    Men    45.689102         STATPOP 2010    2014
## 109 [30,35)    Men    57.799847         STATPOP 2010    2014
## 110 [35,40)    Men    87.822944         STATPOP 2010    2014
## 111 [40,45)    Men   136.639712         STATPOP 2010    2014
## 112 [45,50)    Men   220.696593         STATPOP 2010    2014
## 113 [50,55)    Men   380.091211         STATPOP 2010    2014
## 114 [55,60)    Men   609.904520         STATPOP 2010    2014
## 115 [60,65)    Men   984.993943         STATPOP 2010    2014
## 116 [65,70)    Men  1538.795648         STATPOP 2010    2014
## 117 [70,75)    Men  2485.227398         STATPOP 2010    2014
## 118 [75,80)    Men  4507.669716         STATPOP 2010    2014
## 119 [80,85)    Men  8506.688905         STATPOP 2010    2014
## 120    >=85    Men 18578.520375         STATPOP 2010    2014
## 121 [15,20)  Women    15.684615        SE 2010 (IPW)    2011
## 122 [20,25)  Women    27.715709        SE 2010 (IPW)    2011
## 123 [25,30)  Women     9.716310        SE 2010 (IPW)    2011
## 124 [30,35)  Women    27.841045        SE 2010 (IPW)    2011
## 125 [35,40)  Women    60.433223        SE 2010 (IPW)    2011
## 126 [40,45)  Women    81.036117        SE 2010 (IPW)    2011
```

```
## 127 [45,50)  Women   114.701944        SE 2010 (IPW)    2011
## 128 [50,55)  Women   179.922025        SE 2010 (IPW)    2011
## 129 [55,60)  Women   291.736465        SE 2010 (IPW)    2011
## 130 [60,65)  Women   438.619262        SE 2010 (IPW)    2011
## 131 [65,70)  Women   800.759720        SE 2010 (IPW)    2011
## 132 [70,75)  Women  1393.420275        SE 2010 (IPW)    2011
## 133 [75,80)  Women  2161.942484        SE 2010 (IPW)    2011
## 134 [80,85)  Women  4665.918951        SE 2010 (IPW)    2011
## 135    >=85  Women 12810.055012        SE 2010 (IPW)    2011
## 136 [15,20)    Men    38.210198        SE 2010 (IPW)    2011
## 137 [20,25)    Men    66.460779        SE 2010 (IPW)    2011
## 138 [25,30)    Men    56.949622        SE 2010 (IPW)    2011
## 139 [30,35)    Men    39.680743        SE 2010 (IPW)    2011
## 140 [35,40)    Men    73.923647        SE 2010 (IPW)    2011
## 141 [40,45)    Men   103.655757        SE 2010 (IPW)    2011
## 142 [45,50)    Men   228.583557        SE 2010 (IPW)    2011
## 143 [50,55)    Men   324.506382        SE 2010 (IPW)    2011
## 144 [55,60)    Men   535.927395        SE 2010 (IPW)    2011
## 145 [60,65)    Men   852.643033        SE 2010 (IPW)    2011
## 146 [65,70)    Men  1405.391029        SE 2010 (IPW)    2011
## 147 [70,75)    Men  2235.184810        SE 2010 (IPW)    2011
## 148 [75,80)    Men  3812.699732        SE 2010 (IPW)    2011
## 149 [80,85)    Men  7671.487100        SE 2010 (IPW)    2011
## 150    >=85    Men 16231.446146        SE 2010 (IPW)    2011
## 151 [15,20)  Women    14.447509        SE 2010 (IPW)    2012
## 152 [20,25)  Women    24.816956        SE 2010 (IPW)    2012
## 153 [25,30)  Women    22.713902        SE 2010 (IPW)    2012
## 154 [30,35)  Women    26.886889        SE 2010 (IPW)    2012
## 155 [35,40)  Women    52.017538        SE 2010 (IPW)    2012
## 156 [40,45)  Women    77.176968        SE 2010 (IPW)    2012
## 157 [45,50)  Women   118.901889        SE 2010 (IPW)    2012
## 158 [50,55)  Women   196.007636        SE 2010 (IPW)    2012
## 159 [55,60)  Women   353.125964        SE 2010 (IPW)    2012
## 160 [60,65)  Women   484.483328        SE 2010 (IPW)    2012
## 161 [65,70)  Women   808.092668        SE 2010 (IPW)    2012
## 162 [70,75)  Women  1318.514001        SE 2010 (IPW)    2012
## 163 [75,80)  Women  2373.755434        SE 2010 (IPW)    2012
## 164 [80,85)  Women  5074.020115        SE 2010 (IPW)    2012
## 165    >=85  Women 13666.502739        SE 2010 (IPW)    2012
## 166 [15,20)    Men    40.629824        SE 2010 (IPW)    2012
## 167 [20,25)    Men    59.245098        SE 2010 (IPW)    2012
## 168 [25,30)    Men    50.021893        SE 2010 (IPW)    2012
## 169 [30,35)    Men    55.705254        SE 2010 (IPW)    2012
## 170 [35,40)    Men    85.467709        SE 2010 (IPW)    2012
## 171 [40,45)    Men   116.421434        SE 2010 (IPW)    2012
## 172 [45,50)    Men   216.241968        SE 2010 (IPW)    2012
## 173 [50,55)    Men   340.291332        SE 2010 (IPW)    2012
## 174 [55,60)    Men   555.932389        SE 2010 (IPW)    2012
```

```
## 175 [60,65)    Men   940.193524        SE 2010 (IPW)    2012
## 176 [65,70)    Men  1451.391427        SE 2010 (IPW)    2012
## 177 [70,75)    Men  2199.333817        SE 2010 (IPW)    2012
## 178 [75,80)    Men  4096.313171        SE 2010 (IPW)    2012
## 179 [80,85)    Men  7840.670297        SE 2010 (IPW)    2012
## 180    >=85    Men 17346.539058        SE 2010 (IPW)    2012
## 181 [15,20)  Women    13.054957        SE 2010 (IPW)    2013
## 182 [20,25)  Women    21.615197        SE 2010 (IPW)    2013
## 183 [25,30)  Women    23.322351        SE 2010 (IPW)    2013
## 184 [30,35)  Women    29.733758        SE 2010 (IPW)    2013
## 185 [35,40)  Women    54.769887        SE 2010 (IPW)    2013
## 186 [40,45)  Women    74.086111        SE 2010 (IPW)    2013
## 187 [45,50)  Women   125.644561        SE 2010 (IPW)    2013
## 188 [50,55)  Women   193.726376        SE 2010 (IPW)    2013
## 189 [55,60)  Women   374.806915        SE 2010 (IPW)    2013
## 190 [60,65)  Women   500.709369        SE 2010 (IPW)    2013
## 191 [65,70)  Women   849.125485        SE 2010 (IPW)    2013
## 192 [70,75)  Women  1371.316289        SE 2010 (IPW)    2013
## 193 [75,80)  Women  2559.020267        SE 2010 (IPW)    2013
## 194 [80,85)  Women  5556.271869        SE 2010 (IPW)    2013
## 195    >=85  Women 14582.812370        SE 2010 (IPW)    2013
## 196 [15,20)    Men    41.159052        SE 2010 (IPW)    2013
## 197 [20,25)    Men    62.647232        SE 2010 (IPW)    2013
## 198 [25,30)    Men    43.909027        SE 2010 (IPW)    2013
## 199 [30,35)    Men    59.027649        SE 2010 (IPW)    2013
## 200 [35,40)    Men    93.477442        SE 2010 (IPW)    2013
## 201 [40,45)    Men   124.164388        SE 2010 (IPW)    2013
## 202 [45,50)    Men   215.104181        SE 2010 (IPW)    2013
## 203 [50,55)    Men   345.963266        SE 2010 (IPW)    2013
## 204 [55,60)    Men   581.741658        SE 2010 (IPW)    2013
## 205 [60,65)    Men   984.871604        SE 2010 (IPW)    2013
## 206 [65,70)    Men  1448.240873        SE 2010 (IPW)    2013
## 207 [70,75)    Men  2345.894553        SE 2010 (IPW)    2013
## 208 [75,80)    Men  4244.783718        SE 2010 (IPW)    2013
## 209 [80,85)    Men  8165.166015        SE 2010 (IPW)    2013
## 210    >=85    Men 17658.386464        SE 2010 (IPW)    2013
## 211 [15,20)  Women    12.212780        SE 2010 (IPW)    2014
## 212 [20,25)  Women    22.773451        SE 2010 (IPW)    2014
## 213 [25,30)  Women    23.350364        SE 2010 (IPW)    2014
## 214 [30,35)  Women    29.123226        SE 2010 (IPW)    2014
## 215 [35,40)  Women    52.370768        SE 2010 (IPW)    2014
## 216 [40,45)  Women    77.327303        SE 2010 (IPW)    2014
## 217 [45,50)  Women   131.321278        SE 2010 (IPW)    2014
## 218 [50,55)  Women   209.664953        SE 2010 (IPW)    2014
## 219 [55,60)  Women   362.815403        SE 2010 (IPW)    2014
## 220 [60,65)  Women   521.062804        SE 2010 (IPW)    2014
## 221 [65,70)  Women   871.126183        SE 2010 (IPW)    2014
## 222 [70,75)  Women  1461.575233        SE 2010 (IPW)    2014
```

```
## 223 [75,80)  Women  2762.836441        SE 2010 (IPW)    2014
## 224 [80,85)  Women  5846.337931        SE 2010 (IPW)    2014
## 225    >=85  Women 15001.567906        SE 2010 (IPW)    2014
## 226 [15,20)    Men    43.496695        SE 2010 (IPW)    2014
## 227 [20,25)    Men    57.695570        SE 2010 (IPW)    2014
## 228 [25,30)    Men    48.304200        SE 2010 (IPW)    2014
## 229 [30,35)    Men    57.600581        SE 2010 (IPW)    2014
## 230 [35,40)    Men   100.521760        SE 2010 (IPW)    2014
## 231 [40,45)    Men   126.635596        SE 2010 (IPW)    2014
## 232 [45,50)    Men   216.981483        SE 2010 (IPW)    2014
## 233 [50,55)    Men   370.106818        SE 2010 (IPW)    2014
## 234 [55,60)    Men   603.188809        SE 2010 (IPW)    2014
## 235 [60,65)    Men   992.637845        SE 2010 (IPW)    2014
## 236 [65,70)    Men  1530.377833        SE 2010 (IPW)    2014
## 237 [70,75)    Men  2465.561408        SE 2010 (IPW)    2014
## 238 [75,80)    Men  4455.407102        SE 2010 (IPW)    2014
## 239 [80,85)    Men  8517.180075        SE 2010 (IPW)    2014
## 240    >=85    Men 18015.253971        SE 2010 (IPW)    2014
## 241 [15,20)  Women    21.456667        SE 2010 (CSW)    2011
## 242 [20,25)  Women    45.277300        SE 2010 (CSW)    2011
## 243 [25,30)  Women     4.243447        SE 2010 (CSW)    2011
## 244 [30,35)  Women    36.895489        SE 2010 (CSW)    2011
## 245 [35,40)  Women    64.181996        SE 2010 (CSW)    2011
## 246 [40,45)  Women    47.652872        SE 2010 (CSW)    2011
## 247 [45,50)  Women    75.705444        SE 2010 (CSW)    2011
## 248 [50,55)  Women   121.973648        SE 2010 (CSW)    2011
## 249 [55,60)  Women   213.280274        SE 2010 (CSW)    2011
## 250 [60,65)  Women   329.103396        SE 2010 (CSW)    2011
## 251 [65,70)  Women   533.728428        SE 2010 (CSW)    2011
## 252 [70,75)  Women   759.292139        SE 2010 (CSW)    2011
## 253 [75,80)  Women  1573.960969        SE 2010 (CSW)    2011
## 254 [80,85)  Women  2620.025281        SE 2010 (CSW)    2011
## 255    >=85  Women  7249.268123        SE 2010 (CSW)    2011
## 256 [15,20)    Men    40.608544        SE 2010 (CSW)    2011
## 257 [20,25)    Men    78.746894        SE 2010 (CSW)    2011
## 258 [25,30)    Men    19.121511        SE 2010 (CSW)    2011
## 259 [30,35)    Men    14.100386        SE 2010 (CSW)    2011
## 260 [35,40)    Men    30.461653        SE 2010 (CSW)    2011
## 261 [40,45)    Men    76.867284        SE 2010 (CSW)    2011
## 262 [45,50)    Men   199.434395        SE 2010 (CSW)    2011
## 263 [50,55)    Men   245.975991        SE 2010 (CSW)    2011
## 264 [55,60)    Men   344.806118        SE 2010 (CSW)    2011
## 265 [60,65)    Men   696.648295        SE 2010 (CSW)    2011
## 266 [65,70)    Men  1299.360920        SE 2010 (CSW)    2011
## 267 [70,75)    Men  1538.356480        SE 2010 (CSW)    2011
## 268 [75,80)    Men  3303.494231        SE 2010 (CSW)    2011
## 269 [80,85)    Men  5180.875425        SE 2010 (CSW)    2011
## 270    >=85    Men 13286.071315        SE 2010 (CSW)    2011
```

```
## 271 [15,20)  Women    14.607857        SE 2010 (CSW)    2012
## 272 [20,25)  Women    22.610082        SE 2010 (CSW)    2012
## 273 [25,30)  Women    11.564514        SE 2010 (CSW)    2012
## 274 [30,35)  Women    19.841053        SE 2010 (CSW)    2012
## 275 [35,40)  Women    62.137067        SE 2010 (CSW)    2012
## 276 [40,45)  Women    36.704996        SE 2010 (CSW)    2012
## 277 [45,50)  Women    90.807483        SE 2010 (CSW)    2012
## 278 [50,55)  Women   161.669577        SE 2010 (CSW)    2012
## 279 [55,60)  Women   277.067267        SE 2010 (CSW)    2012
## 280 [60,65)  Women   399.356502        SE 2010 (CSW)    2012
## 281 [65,70)  Women   660.085426        SE 2010 (CSW)    2012
## 282 [70,75)  Women  1147.225340        SE 2010 (CSW)    2012
## 283 [75,80)  Women  1871.054320        SE 2010 (CSW)    2012
## 284 [80,85)  Women  3282.476001        SE 2010 (CSW)    2012
## 285    >=85  Women  8966.303744        SE 2010 (CSW)    2012
## 286 [15,20)    Men    31.562778        SE 2010 (CSW)    2012
## 287 [20,25)    Men    44.683345        SE 2010 (CSW)    2012
## 288 [25,30)    Men    16.457285        SE 2010 (CSW)    2012
## 289 [30,35)    Men    32.615906        SE 2010 (CSW)    2012
## 290 [35,40)    Men    38.971495        SE 2010 (CSW)    2012
## 291 [40,45)    Men    83.349257        SE 2010 (CSW)    2012
## 292 [45,50)    Men   185.482726        SE 2010 (CSW)    2012
## 293 [50,55)    Men   263.154411        SE 2010 (CSW)    2012
## 294 [55,60)    Men   443.093638        SE 2010 (CSW)    2012
## 295 [60,65)    Men   863.379818        SE 2010 (CSW)    2012
## 296 [65,70)    Men  1227.919814        SE 2010 (CSW)    2012
## 297 [70,75)    Men  1780.443705        SE 2010 (CSW)    2012
## 298 [75,80)    Men  3553.545049        SE 2010 (CSW)    2012
## 299 [80,85)    Men  6274.010504        SE 2010 (CSW)    2012
## 300    >=85    Men 13713.128983        SE 2010 (CSW)    2012
## 301 [15,20)  Women    11.007204        SE 2010 (CSW)    2013
## 302 [20,25)  Women    17.187313        SE 2010 (CSW)    2013
## 303 [25,30)  Women     7.713748        SE 2010 (CSW)    2013
## 304 [30,35)  Women    24.535162        SE 2010 (CSW)    2013
## 305 [35,40)  Women    65.499893        SE 2010 (CSW)    2013
## 306 [40,45)  Women    67.901928        SE 2010 (CSW)    2013
## 307 [45,50)  Women   120.278670        SE 2010 (CSW)    2013
## 308 [50,55)  Women   158.817458        SE 2010 (CSW)    2013
## 309 [55,60)  Women   259.228715        SE 2010 (CSW)    2013
## 310 [60,65)  Women   408.437662        SE 2010 (CSW)    2013
## 311 [65,70)  Women   718.421464        SE 2010 (CSW)    2013
## 312 [70,75)  Women  1138.866562        SE 2010 (CSW)    2013
## 313 [75,80)  Women  2066.817700        SE 2010 (CSW)    2013
## 314 [80,85)  Women  3860.621450        SE 2010 (CSW)    2013
## 315    >=85  Women  9935.063566        SE 2010 (CSW)    2013
## 316 [15,20)    Men    40.405691        SE 2010 (CSW)    2013
## 317 [20,25)    Men    47.685162        SE 2010 (CSW)    2013
## 318 [25,30)    Men    30.771165        SE 2010 (CSW)    2013
```

```
## 319 [30,35)    Men    40.012109        SE 2010 (CSW)    2013
## 320 [35,40)    Men    52.669787        SE 2010 (CSW)    2013
## 321 [40,45)    Men    86.193368        SE 2010 (CSW)    2013
## 322 [45,50)    Men   171.997779        SE 2010 (CSW)    2013
## 323 [50,55)    Men   300.908336        SE 2010 (CSW)    2013
## 324 [55,60)    Men   569.837920        SE 2010 (CSW)    2013
## 325 [60,65)    Men   859.399329        SE 2010 (CSW)    2013
## 326 [65,70)    Men  1303.685053        SE 2010 (CSW)    2013
## 327 [70,75)    Men  2077.278485        SE 2010 (CSW)    2013
## 328 [75,80)    Men  3851.692498        SE 2010 (CSW)    2013
## 329 [80,85)    Men  6700.217988        SE 2010 (CSW)    2013
## 330    >=85    Men 14781.732965        SE 2010 (CSW)    2013
## 331 [15,20)  Women    14.688662        SE 2010 (CSW)    2014
## 332 [20,25)  Women    17.038524        SE 2010 (CSW)    2014
## 333 [25,30)  Women    15.321961        SE 2010 (CSW)    2014
## 334 [30,35)  Women    21.331563        SE 2010 (CSW)    2014
## 335 [35,40)  Women    59.400449        SE 2010 (CSW)    2014
## 336 [40,45)  Women    60.080205        SE 2010 (CSW)    2014
## 337 [45,50)  Women   107.031298        SE 2010 (CSW)    2014
## 338 [50,55)  Women   189.927821        SE 2010 (CSW)    2014
## 339 [55,60)  Women   280.057475        SE 2010 (CSW)    2014
## 340 [60,65)  Women   425.408635        SE 2010 (CSW)    2014
## 341 [65,70)  Women   739.424154        SE 2010 (CSW)    2014
## 342 [70,75)  Women  1225.132817        SE 2010 (CSW)    2014
## 343 [75,80)  Women  2325.806829        SE 2010 (CSW)    2014
## 344 [80,85)  Women  4319.360759        SE 2010 (CSW)    2014
## 345    >=85  Women 10787.564510        SE 2010 (CSW)    2014
## 346 [15,20)    Men    39.444701        SE 2010 (CSW)    2014
## 347 [20,25)    Men    53.044022        SE 2010 (CSW)    2014
## 348 [25,30)    Men    37.706197        SE 2010 (CSW)    2014
## 349 [30,35)    Men    37.324914        SE 2010 (CSW)    2014
## 350 [35,40)    Men    65.038989        SE 2010 (CSW)    2014
## 351 [40,45)    Men    91.424783        SE 2010 (CSW)    2014
## 352 [45,50)    Men   183.549675        SE 2010 (CSW)    2014
## 353 [50,55)    Men   329.176764        SE 2010 (CSW)    2014
## 354 [55,60)    Men   566.524369        SE 2010 (CSW)    2014
## 355 [60,65)    Men   894.774731        SE 2010 (CSW)    2014
## 356 [65,70)    Men  1376.427430        SE 2010 (CSW)    2014
## 357 [70,75)    Men  2181.257112        SE 2010 (CSW)    2014
## 358 [75,80)    Men  4019.313475        SE 2010 (CSW)    2014
## 359 [80,85)    Men  7196.404350        SE 2010 (CSW)    2014
## 360    >=85    Men 15402.287561        SE 2010 (CSW)    2014
## 361 [15,20)  Women    20.645554 SE 2010 (unweighted)    2011
## 362 [20,25)  Women    38.138208 SE 2010 (unweighted)    2011
## 363 [25,30)  Women     8.167941 SE 2010 (unweighted)    2011
## 364 [30,35)  Women    44.382437 SE 2010 (unweighted)    2011
## 365 [35,40)  Women    50.265497 SE 2010 (unweighted)    2011
## 366 [40,45)  Women    45.858058 SE 2010 (unweighted)    2011
```

```
## 367 [45,50)  Women    96.517870 SE 2010 (unweighted)    2011
## 368 [50,55)  Women   125.409473 SE 2010 (unweighted)    2011
## 369 [55,60)  Women   233.784901 SE 2010 (unweighted)    2011
## 370 [60,65)  Women   369.309858 SE 2010 (unweighted)    2011
## 371 [65,70)  Women   534.433037 SE 2010 (unweighted)    2011
## 372 [70,75)  Women   718.049265 SE 2010 (unweighted)    2011
## 373 [75,80)  Women  1684.317914 SE 2010 (unweighted)    2011
## 374 [80,85)  Women  2648.064442 SE 2010 (unweighted)    2011
## 375    >=85  Women  7639.300960 SE 2010 (unweighted)    2011
## 376 [15,20)    Men    39.245108 SE 2010 (unweighted)    2011
## 377 [20,25)    Men    60.176419 SE 2010 (unweighted)    2011
## 378 [25,30)    Men    17.127945 SE 2010 (unweighted)    2011
## 379 [30,35)    Men    15.487298 SE 2010 (unweighted)    2011
## 380 [35,40)    Men    29.592019 SE 2010 (unweighted)    2011
## 381 [40,45)    Men    93.282595 SE 2010 (unweighted)    2011
## 382 [45,50)    Men   172.714604 SE 2010 (unweighted)    2011
## 383 [50,55)    Men   262.146560 SE 2010 (unweighted)    2011
## 384 [55,60)    Men   363.033508 SE 2010 (unweighted)    2011
## 385 [60,65)    Men   735.158886 SE 2010 (unweighted)    2011
## 386 [65,70)    Men  1313.294880 SE 2010 (unweighted)    2011
## 387 [70,75)    Men  1532.425302 SE 2010 (unweighted)    2011
## 388 [75,80)    Men  3271.311362 SE 2010 (unweighted)    2011
## 389 [80,85)    Men  4885.207208 SE 2010 (unweighted)    2011
## 390    >=85    Men 13491.370145 SE 2010 (unweighted)    2011
## 391 [15,20)  Women    15.464556 SE 2010 (unweighted)    2012
## 392 [20,25)  Women    19.045162 SE 2010 (unweighted)    2012
## 393 [25,30)  Women    12.236181 SE 2010 (unweighted)    2012
## 394 [30,35)  Women    25.858444 SE 2010 (unweighted)    2012
## 395 [35,40)  Women    53.799156 SE 2010 (unweighted)    2012
## 396 [40,45)  Women    39.260551 SE 2010 (unweighted)    2012
## 397 [45,50)  Women   106.078657 SE 2010 (unweighted)    2012
## 398 [50,55)  Women   169.578873 SE 2010 (unweighted)    2012
## 399 [55,60)  Women   271.373108 SE 2010 (unweighted)    2012
## 400 [60,65)  Women   422.362520 SE 2010 (unweighted)    2012
## 401 [65,70)  Women   671.823608 SE 2010 (unweighted)    2012
## 402 [70,75)  Women  1130.017789 SE 2010 (unweighted)    2012
## 403 [75,80)  Women  1935.177892 SE 2010 (unweighted)    2012
## 404 [80,85)  Women  3317.825556 SE 2010 (unweighted)    2012
## 405    >=85  Women  9379.725566 SE 2010 (unweighted)    2012
## 406 [15,20)    Men    34.299023 SE 2010 (unweighted)    2012
## 407 [20,25)    Men    40.072894 SE 2010 (unweighted)    2012
## 408 [25,30)    Men    17.105799 SE 2010 (unweighted)    2012
## 409 [30,35)    Men    30.937813 SE 2010 (unweighted)    2012
## 410 [35,40)    Men    36.946818 SE 2010 (unweighted)    2012
## 411 [40,45)    Men    93.198479 SE 2010 (unweighted)    2012
## 412 [45,50)    Men   179.024795 SE 2010 (unweighted)    2012
## 413 [50,55)    Men   288.427577 SE 2010 (unweighted)    2012
## 414 [55,60)    Men   449.880530 SE 2010 (unweighted)    2012
```

```
## 415 [60,65)    Men   852.537359 SE 2010 (unweighted)    2012
## 416 [65,70)    Men  1267.009999 SE 2010 (unweighted)    2012
## 417 [70,75)    Men  1845.728817 SE 2010 (unweighted)    2012
## 418 [75,80)    Men  3557.932039 SE 2010 (unweighted)    2012
## 419 [80,85)    Men  6038.180914 SE 2010 (unweighted)    2012
## 420    >=85    Men 13923.237498 SE 2010 (unweighted)    2012
## 421 [15,20)  Women    13.753190 SE 2010 (unweighted)    2013
## 422 [20,25)  Women    15.878930 SE 2010 (unweighted)    2013
## 423 [25,30)  Women     8.161524 SE 2010 (unweighted)    2013
## 424 [30,35)  Women    27.106260 SE 2010 (unweighted)    2013
## 425 [35,40)  Women    59.820720 SE 2010 (unweighted)    2013
## 426 [40,45)  Women    72.038819 SE 2010 (unweighted)    2013
## 427 [45,50)  Women   133.017536 SE 2010 (unweighted)    2013
## 428 [50,55)  Women   179.672138 SE 2010 (unweighted)    2013
## 429 [55,60)  Women   256.513604 SE 2010 (unweighted)    2013
## 430 [60,65)  Women   441.258550 SE 2010 (unweighted)    2013
## 431 [65,70)  Women   726.927169 SE 2010 (unweighted)    2013
## 432 [70,75)  Women  1170.016688 SE 2010 (unweighted)    2013
## 433 [75,80)  Women  2136.452036 SE 2010 (unweighted)    2013
## 434 [80,85)  Women  3813.778276 SE 2010 (unweighted)    2013
## 435    >=85  Women 10154.831313 SE 2010 (unweighted)    2013
## 436 [15,20)    Men    39.223569 SE 2010 (unweighted)    2013
## 437 [20,25)    Men    40.097720 SE 2010 (unweighted)    2013
## 438 [25,30)    Men    31.379307 SE 2010 (unweighted)    2013
## 439 [30,35)    Men    36.118517 SE 2010 (unweighted)    2013
## 440 [35,40)    Men    46.833623 SE 2010 (unweighted)    2013
## 441 [40,45)    Men    95.510317 SE 2010 (unweighted)    2013
## 442 [45,50)    Men   172.869444 SE 2010 (unweighted)    2013
## 443 [50,55)    Men   330.333997 SE 2010 (unweighted)    2013
## 444 [55,60)    Men   581.639291 SE 2010 (unweighted)    2013
## 445 [60,65)    Men   836.665717 SE 2010 (unweighted)    2013
## 446 [65,70)    Men  1365.134510 SE 2010 (unweighted)    2013
## 447 [70,75)    Men  2109.630550 SE 2010 (unweighted)    2013
## 448 [75,80)    Men  3924.672879 SE 2010 (unweighted)    2013
## 449 [80,85)    Men  6665.052569 SE 2010 (unweighted)    2013
## 450    >=85    Men 15216.762417 SE 2010 (unweighted)    2013
## 451 [15,20)  Women    18.056559 SE 2010 (unweighted)    2014
## 452 [20,25)  Women    14.294908 SE 2010 (unweighted)    2014
## 453 [25,30)  Women    16.327812 SE 2010 (unweighted)    2014
## 454 [30,35)  Women    24.033885 SE 2010 (unweighted)    2014
## 455 [35,40)  Women    59.253035 SE 2010 (unweighted)    2014
## 456 [40,45)  Women    65.531434 SE 2010 (unweighted)    2014
## 457 [45,50)  Women   117.567947 SE 2010 (unweighted)    2014
## 458 [50,55)  Women   205.165882 SE 2010 (unweighted)    2014
## 459 [55,60)  Women   286.970484 SE 2010 (unweighted)    2014
## 460 [60,65)  Women   462.344516 SE 2010 (unweighted)    2014
## 461 [65,70)  Women   758.616895 SE 2010 (unweighted)    2014
## 462 [70,75)  Women  1240.075467 SE 2010 (unweighted)    2014
```

```
## 463 [75,80)  Women  2363.626097 SE 2010 (unweighted)    2014
## 464 [80,85)  Women  4279.831524 SE 2010 (unweighted)    2014
## 465     >=85 Women 10952.281523 SE 2010 (unweighted)    2014
## 466 [15,20)    Men    36.787546 SE 2010 (unweighted)    2014
## 467 [20,25)    Men    45.129634 SE 2010 (unweighted)    2014
## 468 [25,30)    Men    34.247037 SE 2010 (unweighted)    2014
## 469 [30,35)    Men    36.779934 SE 2010 (unweighted)    2014
## 470 [35,40)    Men    55.490512 SE 2010 (unweighted)    2014
## 471 [40,45)    Men    98.358112 SE 2010 (unweighted)    2014
## 472 [45,50)    Men   189.097926 SE 2010 (unweighted)    2014
## 473 [50,55)    Men   349.823228 SE 2010 (unweighted)    2014
## 474 [55,60)    Men   585.923069 SE 2010 (unweighted)    2014
## 475 [60,65)    Men   900.420635 SE 2010 (unweighted)    2014
## 476 [65,70)    Men  1403.658926 SE 2010 (unweighted)    2014
## 477 [70,75)    Men  2196.843476 SE 2010 (unweighted)    2014
## 478 [75,80)    Men  4106.057230 SE 2010 (unweighted)    2014
## 479 [80,85)    Men  7147.451423 SE 2010 (unweighted)    2014
## 480     >=85   Men 15952.414381 SE 2010 (unweighted)    2014
```

```r
p <- ggplot(data=output, aes(x=agecat, y=rate, color=type))
p1 <- p + geom_point(size=0.7) + geom_line(aes(group=type), size=0.3)
p1 <- p1 + scale_y_log10(breaks=c(5,10,20,100,1000,10000)) + facet_grid(yeardod~female)
p1 <- p1 +  ylab("Mortality rate (per 100,000 person-years)") + xlab("Age categories")
p1 <- p1 + theme_bw()
p1 <- p1 + theme(strip.background = element_rect(fill="#F5F5F5"),
             legend.key = element_blank(),
             axis.text.x = element_text(angle=45, vjust=0.6),
             legend.position="bottom")
p1 <- p1 + scale_color_brewer("", palette = "Set1")
p1
```

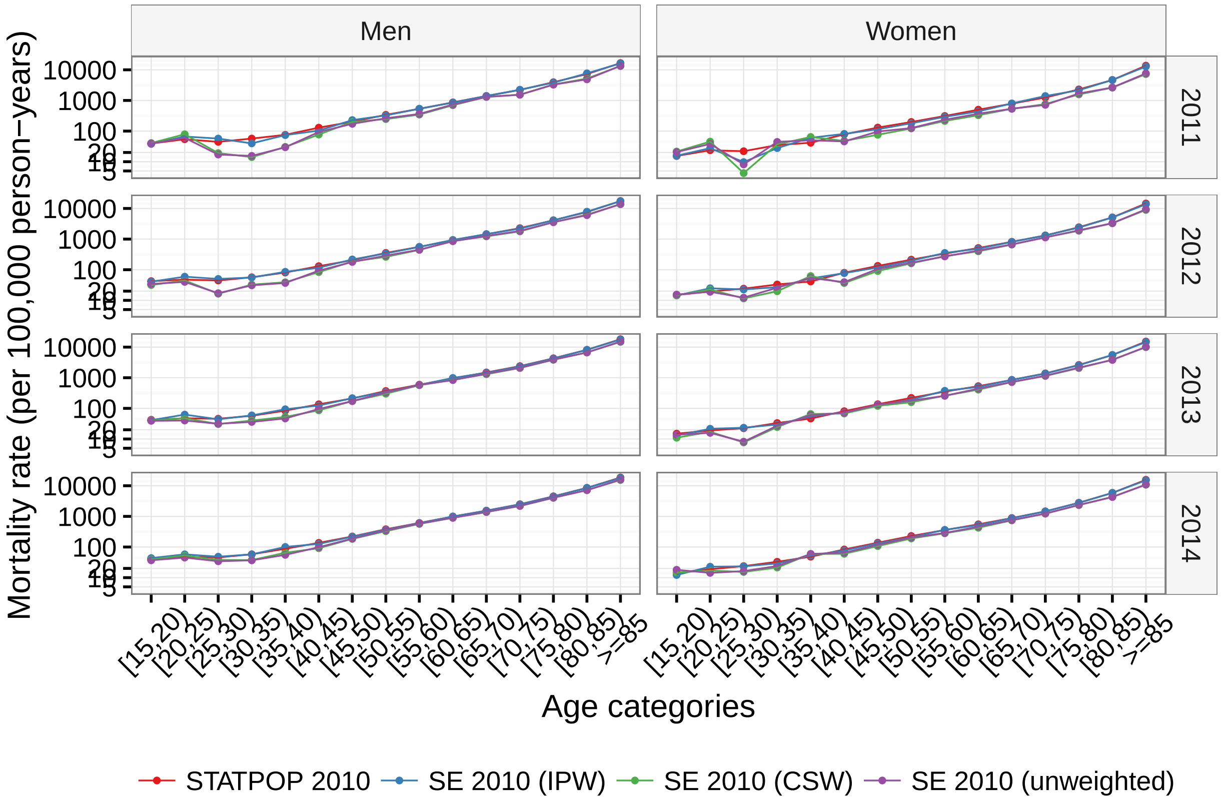


```
ggsave(file="F:\\SNC\\SurveyMortality\\SupFig1.tiff", plot=p1,
       width=19.05, height=22.23, dpi=600, unit="cm",
       compression="lzw", type="cairo")
ggsave(file="F:\\SNC\\SurveyMortality\\SupFig1.png", plot=p1,
       width=19.05, height=22.23, dpi=600, unit="cm")
ggsave(file="F:\\SNC\\SurveyMortality\\SupFig1.pdf", plot=p1,
       width=19.05, height=22.23, dpi=600, unit="cm")
```

```
p <- ggplot(data=output[output$yeardod==2011, ], aes(x=agecat, y=rate, color=type))
p1 <- p + geom_point(size=0.7) + geom_line(aes(group=type), size=0.3)
p1 <- p1 + scale_y_log10(breaks=c(5,10,20,100,1000,10000)) + facet_grid(female~.)
p1 <- p1 +  ylab("Mortality rate (per 100,000 person-years)") + xlab("Age categories")
p1 <- p1 + theme_bw()
p1 <- p1 + theme(strip.background = element_rect(fill="#F5F5F5"),
                 legend.key = element_blank(),
                 axis.text.x = element_text(angle=45, vjust=0.6))
p1 <- p1 + scale_color_brewer("", palette = "Set1")
p1
```

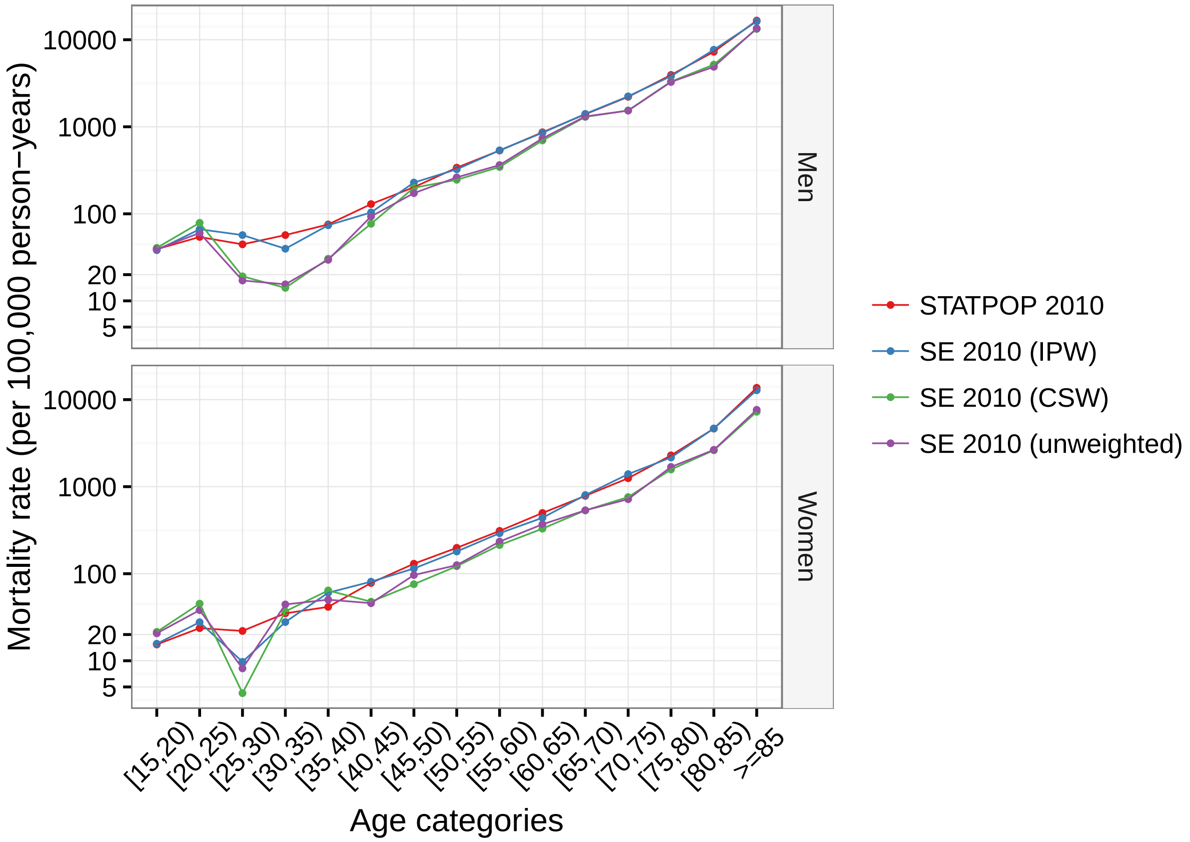


```
ggsave(file="F:\\SNC\\SurveyMortality\\Fig1.tiff", plot=p1,
       width=19.05, height=22.23, dpi=600, unit="cm",
       compression="lzw", type="cairo")
ggsave(file="F:\\SNC\\SurveyMortality\\Fig1.png", plot=p1,
       width=19.05, height=22.23, dpi=600, unit="cm")
ggsave(file="F:\\SNC\\SurveyMortality\\Fig1.pdf", plot=p1,
       width=19.05, height=22.23, dpi=600, unit="cm")
```

Supplemental Figure 2

```
data$r11_statpop_agecat5 <- as.numeric(data$r11_statpop_agecat5)-1
data$r12_statpop_agecat5 <- as.numeric(data$r12_statpop_agecat5)-1
data$r13_statpop_agecat5 <- as.numeric(data$r13_statpop_agecat5)-1

output <- c()

data$fupt2011 <- (data$statpop2010_enddate2011-data$statpop2010_startdate)/365.25
data$fupt2012 <- (data$statpop2011_enddate2012-data$statpop2011_startdate)/365.25
data$fupt2013 <- (data$statpop2012_enddate2013-data$statpop2012_startdate)/365.25
data$fupt2014 <- (data$statpop2013_enddate2014-data$statpop2013_startdate)/365.25
```

```
### Mortality rates of STATPOP population, by gender

type <- 1

year <- 2011
sex <- 1

mod <- glm(died2011 ~ factor(r10_statpop_agecat5)-1, data=data,
           offset=log(fupt2011), subset=female==sex & statpop2010==1, family=poisson())

output <- rbind(output, data.frame(agecat=as.numeric(mod$xlevels[[1]]),
                          female=sex, rate=exp(mod$coeff), type=type, yeardod=year))

sex <- 0

mod <- glm(died2011 ~ factor(r10_statpop_agecat5)-1, data=data,
           offset=log(fupt2011), subset=female==sex & statpop2010==1, family=poisson())

output <- rbind(output, data.frame(agecat=as.numeric(mod$xlevels[[1]]),
                          female=sex, rate=exp(mod$coeff), type=type, yeardod=year))


year <- 2012
sex <- 1

mod <- glm(died2012 ~ factor(r11_statpop_agecat5)-1, data=data,
           offset=log(fupt2012), subset=female==sex & statpop2011==1, family=poisson())

output <- rbind(output, data.frame(agecat=as.numeric(mod$xlevels[[1]]),
                          female=sex, rate=exp(mod$coeff), type=type, yeardod=year))

sex <- 0

mod <- glm(died2012 ~ factor(r11_statpop_agecat5)-1, data=data,
           offset=log(fupt2012), subset=female==sex & statpop2011==1, family=poisson())

output <- rbind(output, data.frame(agecat=as.numeric(mod$xlevels[[1]]),
                          female=sex, rate=exp(mod$coeff), type=type, yeardod=year))


year <- 2013
sex <- 1

mod <- glm(died2013 ~ factor(r12_statpop_agecat5)-1, data=data,
           offset=log(fupt2013), subset=female==sex & statpop2012==1, family=poisson())

output <- rbind(output, data.frame(agecat=as.numeric(mod$xlevels[[1]]),
```

```
                          female=sex, rate=exp(mod$coeff), type=type, yeardod=year))

sex <- 0

mod <- glm(died2013 ~ factor(r12_statpop_agecat5)-1, data=data,
           offset=log(fupt2013), subset=female==sex & statpop2012==1, family=poisson())

output <- rbind(output, data.frame(agecat=as.numeric(mod$xlevels[[1]]),
                          female=sex, rate=exp(mod$coeff), type=type, yeardod=year))


year <- 2014
sex <- 1

mod <- glm(died2014 ~ factor(r13_statpop_agecat5)-1, data=data,
           offset=log(fupt2014), subset=female==sex & statpop2013==1, family=poisson())

output <- rbind(output, data.frame(agecat=as.numeric(mod$xlevels[[1]]),
                          female=sex, rate=exp(mod$coeff), type=type, yeardod=year))

sex <- 0

mod <- glm(died2014 ~ factor(r13_statpop_agecat5)-1, data=data,
           offset=log(fupt2014), subset=female==sex & statpop2013==1, family=poisson())

output <- rbind(output, data.frame(agecat=as.numeric(mod$xlevels[[1]]),
                          female=sex, rate=exp(mod$coeff), type=type, yeardod=year))



### Mortality rates of SE population, by gender, using CS weights (type=2)

type <- 2

year <- 2011
sex <- 1

mod <- glm(died2011 ~ factor(r10_statpop_agecat5)-1, data=data, weights=weight2010,
           offset=log(fupt2011), subset=female==sex & inse2010==1, family=poisson())

output <- rbind(output, data.frame(agecat=as.numeric(mod$xlevels[[1]]),
                          female=sex, rate=exp(mod$coeff), type=type, yeardod=year))

sex <- 0

mod <- glm(died2011 ~ factor(r10_statpop_agecat5)-1, data=data, weights=weight2010,
           offset=log(fupt2011), subset=female==sex & inse2010==1, family=poisson())
```

```
output <- rbind(output, data.frame(agecat=as.numeric(mod$xlevels[[1]]),
                          female=sex, rate=exp(mod$coeff), type=type, yeardod=year))


year <- 2012
sex <- 1

mod <- glm(died2012 ~ factor(r11_statpop_agecat5)-1, data=data, weights=weight2011,
           offset=log(fupt2012), subset=female==sex & inse2011==1, family=poisson())

output <- rbind(output, data.frame(agecat=as.numeric(mod$xlevels[[1]]),
                          female=sex, rate=exp(mod$coeff), type=type, yeardod=year))

sex <- 0

mod <- glm(died2012 ~ factor(r11_statpop_agecat5)-1, data=data, weights=weight2011,
           offset=log(fupt2012), subset=female==sex & inse2011==1, family=poisson())

output <- rbind(output, data.frame(agecat=as.numeric(mod$xlevels[[1]]),
                          female=sex, rate=exp(mod$coeff), type=type, yeardod=year))


year <- 2013
sex <- 1

mod <- glm(died2013 ~ factor(r12_statpop_agecat5)-1, data=data, weights=weight2012,
           offset=log(fupt2013), subset=female==sex & inse2012==1, family=poisson())

output <- rbind(output, data.frame(agecat=as.numeric(mod$xlevels[[1]]),
                          female=sex, rate=exp(mod$coeff), type=type, yeardod=year))

sex <- 0

mod <- glm(died2013 ~ factor(r12_statpop_agecat5)-1, data=data, weights=weight2012,
           offset=log(fupt2013), subset=female==sex & inse2012==1, family=poisson())

output <- rbind(output, data.frame(agecat=as.numeric(mod$xlevels[[1]]),
                          female=sex, rate=exp(mod$coeff), type=type, yeardod=year))


year <- 2014
sex <- 1

mod <- glm(died2014 ~ factor(r13_statpop_agecat5)-1, data=data, weights=weight2013,
           offset=log(fupt2014), subset=female==sex & inse2013==1, family=poisson())

output <- rbind(output, data.frame(agecat=as.numeric(mod$xlevels[[1]]),
```

```
                          female=sex, rate=exp(mod$coeff), type=type, yeardod=year))

sex <- 0

mod <- glm(died2014 ~ factor(r13_statpop_agecat5)-1, data=data, weights=weight2013,
           offset=log(fupt2014), subset=female==sex & inse2013==1, family=poisson())

output <- rbind(output, data.frame(agecat=as.numeric(mod$xlevels[[1]]),
                          female=sex, rate=exp(mod$coeff), type=type, yeardod=year))


output <- output[output$agecat>=3, ]

output$agecat <- factor(output$agecat, levels=0:14,
                           labels=c("[15,20)", "[20,25)", "[25,30)", "[30,35)",
                                    "[35,40)", "[40,45)", "[45,50)", "[50,55)",
                                    "[55,60)", "[60,65)", "[65,70)", "[70,75)" ,
                                    "[75,80)", "[80,85)", ">=85" ))

output$female <- factor(output$female, levels=0:1, labels=c("Men", "Women"))
output$type <- factor(output$type, levels=1:2, labels=c("STATPOP",
                            "SE (CSW)"))

factor <- 100000
output$rate <- output$rate*factor
dimnames(output)[[1]] <- 1:length(dimnames(output)[[1]])
```

```
### Show results
output
```

```
##      agecat female         rate    type yeardod
## 1   [30,35)  Women    35.062308 STATPOP    2011
## 2   [35,40)  Women    41.592267 STATPOP    2011
## 3   [40,45)  Women    78.256865 STATPOP    2011
## 4   [45,50)  Women   130.161288 STATPOP    2011
## 5   [50,55)  Women   198.162944 STATPOP    2011
## 6   [55,60)  Women   310.313967 STATPOP    2011
## 7   [60,65)  Women   498.682109 STATPOP    2011
## 8   [65,70)  Women   784.426453 STATPOP    2011
## 9   [70,75)  Women  1250.939370 STATPOP    2011
## 10  [75,80)  Women  2287.394228 STATPOP    2011
## 11  [80,85)  Women  4645.092145 STATPOP    2011
## 12     >=85  Women 13669.350375 STATPOP    2011
## 13  [30,35)    Men    56.967733 STATPOP    2011
## 14  [35,40)    Men    75.507154 STATPOP    2011
## 15  [40,45)    Men   129.286238 STATPOP    2011
## 16  [45,50)    Men   202.047237 STATPOP    2011
## 17  [50,55)    Men   338.889367 STATPOP    2011
```

```
## 18  [55,60)    Men   532.989780  STATPOP    2011
## 19  [60,65)    Men   864.083681  STATPOP    2011
## 20  [65,70)    Men  1392.860068  STATPOP    2011
## 21  [70,75)    Men  2208.871129  STATPOP    2011
## 22  [75,80)    Men  3936.307593  STATPOP    2011
## 23  [80,85)    Men  7272.737379  STATPOP    2011
## 24     >=85    Men 16596.803811  STATPOP    2011
## 25  [30,35)  Women    25.485154  STATPOP    2012
## 26  [35,40)  Women    40.771513  STATPOP    2012
## 27  [40,45)  Women    69.132307  STATPOP    2012
## 28  [45,50)  Women   130.962279  STATPOP    2012
## 29  [50,55)  Women   197.904733  STATPOP    2012
## 30  [55,60)  Women   334.607082  STATPOP    2012
## 31  [60,65)  Women   498.432033  STATPOP    2012
## 32  [65,70)  Women   781.873916  STATPOP    2012
## 33  [70,75)  Women  1207.991590  STATPOP    2012
## 34  [75,80)  Women  2228.529815  STATPOP    2012
## 35  [80,85)  Women  4867.922119  STATPOP    2012
## 36     >=85  Women 14245.605348  STATPOP    2012
## 37  [30,35)    Men    55.528449  STATPOP    2012
## 38  [35,40)    Men    75.793805  STATPOP    2012
## 39  [40,45)    Men   120.303903  STATPOP    2012
## 40  [45,50)    Men   182.435978  STATPOP    2012
## 41  [50,55)    Men   333.641333  STATPOP    2012
## 42  [55,60)    Men   536.261288  STATPOP    2012
## 43  [60,65)    Men   899.763774  STATPOP    2012
## 44  [65,70)    Men  1371.619464  STATPOP    2012
## 45  [70,75)    Men  2111.055563  STATPOP    2012
## 46  [75,80)    Men  3865.215003  STATPOP    2012
## 47  [80,85)    Men  7008.841818  STATPOP    2012
## 48     >=85    Men 17014.939032  STATPOP    2012
## 49  [30,35)  Women    25.714254  STATPOP    2013
## 50  [35,40)  Women    48.471749  STATPOP    2013
## 51  [40,45)  Women    68.919688  STATPOP    2013
## 52  [45,50)  Women   116.988831  STATPOP    2013
## 53  [50,55)  Women   198.045814  STATPOP    2013
## 54  [55,60)  Women   319.588294  STATPOP    2013
## 55  [60,65)  Women   496.661279  STATPOP    2013
## 56  [65,70)  Women   766.324361  STATPOP    2013
## 57  [70,75)  Women  1227.692108  STATPOP    2013
## 58  [75,80)  Women  2250.900975  STATPOP    2013
## 59  [80,85)  Women  4709.929853  STATPOP    2013
## 60     >=85  Women 14499.946759  STATPOP    2013
## 61  [30,35)    Men    54.715965  STATPOP    2013
## 62  [35,40)    Men    68.964615  STATPOP    2013
## 63  [40,45)    Men   122.660346  STATPOP    2013
## 64  [45,50)    Men   184.938981  STATPOP    2013
## 65  [50,55)    Men   328.739546  STATPOP    2013
```

```
## 66  [55,60)    Men   543.256241  STATPOP    2013
## 67  [60,65)    Men   868.206288  STATPOP    2013
## 68  [65,70)    Men  1343.736575  STATPOP    2013
## 69  [70,75)    Men  2152.700534  STATPOP    2013
## 70  [75,80)    Men  3681.500867  STATPOP    2013
## 71  [80,85)    Men  7203.545356  STATPOP    2013
## 72     >=85    Men 17158.774572  STATPOP    2013
## 73  [30,35)  Women    27.592801  STATPOP    2014
## 74  [35,40)  Women    44.534536  STATPOP    2014
## 75  [40,45)  Women    62.197836  STATPOP    2014
## 76  [45,50)  Women   110.947545  STATPOP    2014
## 77  [50,55)  Women   194.079410  STATPOP    2014
## 78  [55,60)  Women   298.414699  STATPOP    2014
## 79  [60,65)  Women   458.476079  STATPOP    2014
## 80  [65,70)  Women   751.711597  STATPOP    2014
## 81  [70,75)  Women  1221.621228  STATPOP    2014
## 82  [75,80)  Women  2196.718240  STATPOP    2014
## 83  [80,85)  Women  4519.839685  STATPOP    2014
## 84     >=85  Women 13853.492331  STATPOP    2014
## 85  [30,35)    Men    55.447559  STATPOP    2014
## 86  [35,40)    Men    76.276411  STATPOP    2014
## 87  [40,45)    Men   116.065093  STATPOP    2014
## 88  [45,50)    Men   179.267273  STATPOP    2014
## 89  [50,55)    Men   307.619415  STATPOP    2014
## 90  [55,60)    Men   530.059329  STATPOP    2014
## 91  [60,65)    Men   843.207753  STATPOP    2014
## 92  [65,70)    Men  1362.444209  STATPOP    2014
## 93  [70,75)    Men  2068.395413  STATPOP    2014
## 94  [75,80)    Men  3564.881722  STATPOP    2014
## 95  [80,85)    Men  6815.237795  STATPOP    2014
## 96     >=85    Men 16573.565079  STATPOP    2014
## 97  [30,35)  Women    36.895489 SE (CSW)    2011
## 98  [35,40)  Women    64.181996 SE (CSW)    2011
## 99  [40,45)  Women    47.652872 SE (CSW)    2011
## 100 [45,50)  Women    75.705444 SE (CSW)    2011
## 101 [50,55)  Women   121.973648 SE (CSW)    2011
## 102 [55,60)  Women   213.280274 SE (CSW)    2011
## 103 [60,65)  Women   329.103396 SE (CSW)    2011
## 104 [65,70)  Women   533.728428 SE (CSW)    2011
## 105 [70,75)  Women   759.292139 SE (CSW)    2011
## 106 [75,80)  Women  1573.960969 SE (CSW)    2011
## 107 [80,85)  Women  2620.025281 SE (CSW)    2011
## 108    >=85  Women  7249.268123 SE (CSW)    2011
## 109 [30,35)    Men    14.100386 SE (CSW)    2011
## 110 [35,40)    Men    30.461653 SE (CSW)    2011
## 111 [40,45)    Men    76.867284 SE (CSW)    2011
## 112 [45,50)    Men   199.434395 SE (CSW)    2011
## 113 [50,55)    Men   245.975991 SE (CSW)    2011
```

```
## 114 [55,60)    Men   344.806118 SE (CSW)    2011
## 115 [60,65)    Men   696.648295 SE (CSW)    2011
## 116 [65,70)    Men  1299.360920 SE (CSW)    2011
## 117 [70,75)    Men  1538.356480 SE (CSW)    2011
## 118 [75,80)    Men  3303.494231 SE (CSW)    2011
## 119 [80,85)    Men  5180.875425 SE (CSW)    2011
## 120    >=85    Men 13286.071315 SE (CSW)    2011
## 121 [30,35)  Women    12.259392 SE (CSW)    2012
## 122 [35,40)  Women    46.725521 SE (CSW)    2012
## 123 [40,45)  Women    42.565044 SE (CSW)    2012
## 124 [45,50)  Women    72.387047 SE (CSW)    2012
## 125 [50,55)  Women   153.714257 SE (CSW)    2012
## 126 [55,60)  Women   245.327048 SE (CSW)    2012
## 127 [60,65)  Women   328.551409 SE (CSW)    2012
## 128 [65,70)  Women   505.597390 SE (CSW)    2012
## 129 [70,75)  Women   937.938426 SE (CSW)    2012
## 130 [75,80)  Women  1313.434501 SE (CSW)    2012
## 131 [80,85)  Women  2730.393376 SE (CSW)    2012
## 132    >=85  Women  7025.869985 SE (CSW)    2012
## 133 [30,35)    Men    46.783155 SE (CSW)    2012
## 134 [35,40)    Men    64.178790 SE (CSW)    2012
## 135 [40,45)    Men   109.379289 SE (CSW)    2012
## 136 [45,50)    Men   131.180082 SE (CSW)    2012
## 137 [50,55)    Men   220.740787 SE (CSW)    2012
## 138 [55,60)    Men   357.503371 SE (CSW)    2012
## 139 [60,65)    Men   668.982101 SE (CSW)    2012
## 140 [65,70)    Men  1190.903497 SE (CSW)    2012
## 141 [70,75)    Men  1542.188004 SE (CSW)    2012
## 142 [75,80)    Men  2801.273729 SE (CSW)    2012
## 143 [80,85)    Men  5542.320745 SE (CSW)    2012
## 144    >=85    Men 10237.462033 SE (CSW)    2012
## 145 [30,35)  Women    11.323609 SE (CSW)    2013
## 146 [35,40)  Women    12.385474 SE (CSW)    2013
## 147 [40,45)  Women    66.762551 SE (CSW)    2013
## 148 [45,50)  Women    77.290273 SE (CSW)    2013
## 149 [50,55)  Women   147.410516 SE (CSW)    2013
## 150 [55,60)  Women   220.622520 SE (CSW)    2013
## 151 [60,65)  Women   331.242620 SE (CSW)    2013
## 152 [65,70)  Women   557.814077 SE (CSW)    2013
## 153 [70,75)  Women   894.805740 SE (CSW)    2013
## 154 [75,80)  Women  1022.743087 SE (CSW)    2013
## 155 [80,85)  Women  2531.077084 SE (CSW)    2013
## 156    >=85  Women  7960.059586 SE (CSW)    2013
## 157 [30,35)    Men    49.905976 SE (CSW)    2013
## 158 [35,40)    Men     5.659516 SE (CSW)    2013
## 159 [40,45)    Men    97.493674 SE (CSW)    2013
## 160 [45,50)    Men   199.932475 SE (CSW)    2013
## 161 [50,55)    Men   201.184538 SE (CSW)    2013
```

```
## 162 [55,60)    Men   331.644887 SE (CSW)    2013
## 163 [60,65)    Men   611.553884 SE (CSW)    2013
## 164 [65,70)    Men  1125.550042 SE (CSW)    2013
## 165 [70,75)    Men  1510.732732 SE (CSW)    2013
## 166 [75,80)    Men  3011.930342 SE (CSW)    2013
## 167 [80,85)    Men  4771.942046 SE (CSW)    2013
## 168    >=85    Men 10169.148810 SE (CSW)    2013
## 169 [30,35)  Women     7.051458 SE (CSW)    2014
## 170 [35,40)  Women    54.166569 SE (CSW)    2014
## 171 [40,45)  Women    10.926493 SE (CSW)    2014
## 172 [45,50)  Women    87.760568 SE (CSW)    2014
## 173 [50,55)  Women   124.792454 SE (CSW)    2014
## 174 [55,60)  Women   267.853105 SE (CSW)    2014
## 175 [60,65)  Women   363.052761 SE (CSW)    2014
## 176 [65,70)  Women   492.332203 SE (CSW)    2014
## 177 [70,75)  Women   654.619153 SE (CSW)    2014
## 178 [75,80)  Women  1370.321368 SE (CSW)    2014
## 179 [80,85)  Women  2546.424262 SE (CSW)    2014
## 180    >=85  Women  6801.968468 SE (CSW)    2014
## 181 [30,35)    Men    25.189327 SE (CSW)    2014
## 182 [35,40)    Men    77.700629 SE (CSW)    2014
## 183 [40,45)    Men    84.094991 SE (CSW)    2014
## 184 [45,50)    Men   129.863804 SE (CSW)    2014
## 185 [50,55)    Men   265.941088 SE (CSW)    2014
## 186 [55,60)    Men   346.891577 SE (CSW)    2014
## 187 [60,65)    Men   633.386378 SE (CSW)    2014
## 188 [65,70)    Men  1045.501380 SE (CSW)    2014
## 189 [70,75)    Men  1436.359325 SE (CSW)    2014
## 190 [75,80)    Men  2712.867779 SE (CSW)    2014
## 191 [80,85)    Men  4981.144258 SE (CSW)    2014
## 192    >=85    Men 10260.301721 SE (CSW)    2014
```

```
p <- ggplot(data=output, aes(x=agecat, y=rate, color=type))
p1 <- p + geom_point(size=0.7) + geom_line(aes(group=type), size=0.3)
p1 <- p1 + scale_y_log10(breaks=c(5,10,20,100,1000,10000)) + facet_grid(yeardod~female)
p1 <- p1 +  ylab("Mortality rate (per 100,000 person-years)") + xlab("Age categories")
p1 <- p1 + theme_bw()
p1 <- p1 + theme(strip.background = element_rect(fill="#F5F5F5"),
                 legend.key = element_blank(),
                 axis.text.x = element_text(angle=45, vjust=0.6),
                 legend.position="bottom")
p1 <- p1 + scale_color_brewer("", palette = "Set1")
p1
```

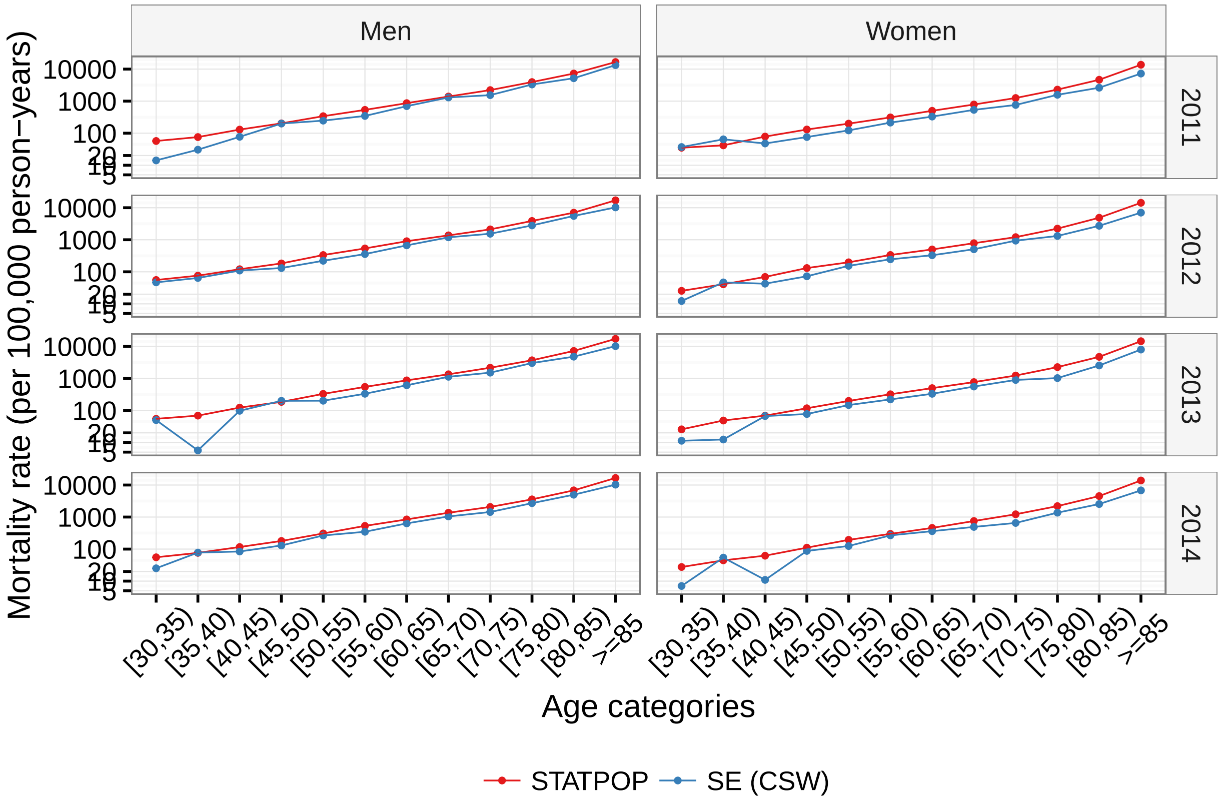


```
ggsave(file="F:\\SNC\\SurveyMortality\\SupFig2.tiff", plot=p1,
       width=19.05, height=22.23, dpi=600, unit="cm",
       compression="lzw", type="cairo")
ggsave(file="F:\\SNC\\SurveyMortality\\SupFig2.png", plot=p1,
       width=19.05, height=22.23, dpi=600, unit="cm")
ggsave(file="F:\\SNC\\SurveyMortality\\SupFig2.pdf", plot=p1,
       width=19.05, height=22.23, dpi=600, unit="cm")
```

Life expectancy (Stata code)

Own written Stata command based on:

Moser A., Clough-Gorr K., Zwahlen M. 2015. Modeling absolute differences in life expectancy with a censored skew-normal regression approach. PeerJ 3:e1162. DOI: 10.7717/peerj.1162.

Stata code and command is available at https://github.com/MoserGitHub/censn.

```
-------------------------------------------------------------------------------------
------
      name:  <unnamed>
       log:  F:\SNC\SurveyMortality\LE_orig_Nov2017.txt
  log type:  text
 opened on:   4 Nov 2017, 14:45:09

.
. use using "F:\SNC\SurveyMortality\data\SwissCensuses.dta", clear
(STATPOP 2010 person data, original)

.
. adopath + "F:\SNC\SurveyMortality\ado\"
  [1]  (BASE)      "C:\Program Files (x86)\Stata14\ado\base/"
  [2]  (SITE)      "C:\Program Files (x86)\Stata14\ado\site/"
  [3]              "."
  [4]  (PERSONAL)  "c:\ado\personal/"
  [5]  (PLUS)      "c:\ado\plus/"
  [6]  (OLDPLACE)  "c:\ado/"
  [7]              "F:\SNC\SurveyMortality\ado\"

.
. keep if statpop2010==1
(553,106 observations deleted)

.
. gen agestart=(statpop2010_startdate-dob)/365.25

. gen ageend=(statpop2010_enddate2011-dob)/365.25

.
. replace agestart=agestart-30
(6,729,363 real changes made)

. replace ageend=ageend-30
(6,729,363 real changes made)

.
. * Restrict to individuals aged 30 years or older
. drop if agestart<0
(1,487,176 observations deleted)

.
. * STATPOP 2010
.
. censn ageend if female==0, lefttrun(agestart) failure(died2011)

Fitting constant-only model:

Iteration 0:   log likelihood = -203980.21  (not concave)
Iteration 1:   log likelihood = -128122.38  (not concave)
Iteration 2:   log likelihood = -120807.65
Iteration 3:   log likelihood = -119626.51
Iteration 4:   log likelihood = -119490.28
Iteration 5:   log likelihood = -119486.94
Iteration 6:   log likelihood = -119486.93

                                                Number of obs     =  2,543,103
                                                Wald chi2(0)      =          .
Log likelihood = -119486.93                     Prob > chi2       =          .

------------------------------------------------------------------------------
             |      Coef.   Std. Err.      z    P>|z|     [95% Conf. Interval]
-------------+----------------------------------------------------------------
mu           |
       _cons |   50.91063   .0568967   894.79   0.000     50.79912    51.02215
-------------+----------------------------------------------------------------
sigma        |
```

```
       _cons |   11.81454   .0443558   266.36   0.000      11.7276    11.90148
-------------+----------------------------------------------------------------
shape        |
       _cons |  -4.936925   .1105467   -44.66   0.000    -5.153592   -4.720257
------------------------------------------------------------------------------

Skewness parameter in CP

Coef.: -.8477204

Std. Err.: .00029257

[95% CI]: -.84829383 , -.84714697

. censn ageend if female==1, lefttrun(agestart) failure(died2011)

Fitting constant-only model:

Iteration 0:   log likelihood = -204870.34  (not concave)
Iteration 1:   log likelihood = -197569.23  (not concave)
Iteration 2:   log likelihood = -125087.72
Iteration 3:   log likelihood = -121848.83
Iteration 4:   log likelihood = -120934.15
Iteration 5:   log likelihood = -120911.86
Iteration 6:   log likelihood = -120911.69
Iteration 7:   log likelihood = -120911.69

                                                Number of obs     =  2,699,084
                                                Wald chi2(0)      =          .
Log likelihood = -120911.69                     Prob > chi2       =          .

------------------------------------------------------------------------------
             |      Coef.   Std. Err.      z    P>|z|     [95% Conf. Interval]
-------------+----------------------------------------------------------------
mu           |
       _cons |   54.77967   .0525087  1043.25   0.000     54.67676    54.88259
-------------+----------------------------------------------------------------
sigma        |
       _cons |   10.93973   .0401365   272.56   0.000     10.86107     11.0184
-------------+----------------------------------------------------------------
shape        |
       _cons |  -5.190304   .1015822   -51.09   0.000    -5.389401   -4.991206
------------------------------------------------------------------------------

Skewness parameter in CP

Coef.: -.86015865

Std. Err.: .00022725

[95% CI]: -.86060405 , -.85971325

.
. * SE 2010
.
. * Unweighted
. censn ageend if female==0 & inse2010==1, lefttrun(agestart) failure(died2011)
diffic
> ult

Fitting constant-only model:

Iteration 0:   log likelihood = -9157.4857  (not concave)
Iteration 1:   log likelihood = -5661.1491  (not concave)
Iteration 2:   log likelihood = -4891.1927  (not concave)
Iteration 3:   log likelihood = -4718.4709  (not concave)
Iteration 4:   log likelihood = -4701.5464
Iteration 5:   log likelihood = -4699.8626
```

```
Iteration 6:   log likelihood = -4699.8225
Iteration 7:   log likelihood = -4699.8224

                                                Number of obs     =    120,916
                                                Wald chi2(0)      =          .
Log likelihood = -4699.8224                     Prob > chi2       =          .

------------------------------------------------------------------------------
             |      Coef.   Std. Err.      z    P>|z|     [95% Conf. Interval]
-------------+----------------------------------------------------------------
mu           |
       _cons |   53.32098   .2830639   188.37   0.000     52.76619    53.87578
-------------+----------------------------------------------------------------
sigma        |
       _cons |    11.6091   .2230082    52.06   0.000     11.17201    12.04619
-------------+----------------------------------------------------------------
shape        |
       _cons |  -5.177814   .7836814    -6.61   0.000    -6.713802   -3.641827
------------------------------------------------------------------------------

Skewness parameter in CP

Coef.: -.85958137

Std. Err.: .07756589

[95% CI]: -1.0116077 , -.70755501

. censn ageend if female==1 & inse2010==1, lefttrun(agestart) failure(died2011)
diffic
> ult

Fitting constant-only model:

Iteration 0:   log likelihood = -8930.7283  (not concave)
Iteration 1:   log likelihood = -4912.0185  (not concave)
Iteration 2:   log likelihood = -4819.6846  (not concave)
Iteration 3:   log likelihood = -4559.9762  (not concave)
Iteration 4:   log likelihood = -4466.8084  (not concave)
Iteration 5:   log likelihood = -4162.7452  (not concave)
Iteration 6:   log likelihood = -4051.9712
Iteration 7:   log likelihood = -4051.5653
Iteration 8:   log likelihood = -3976.5086
Iteration 9:   log likelihood = -3973.9568
Iteration 10:  log likelihood = -3972.4284
Iteration 11:  log likelihood = -3972.2223
Iteration 12:  log likelihood = -3972.1943
Iteration 13:  log likelihood = -3972.1941

                                                Number of obs     =    131,852
                                                Wald chi2(0)      =          .
Log likelihood = -3972.1941                     Prob > chi2       =          .

------------------------------------------------------------------------------
             |      Coef.   Std. Err.      z    P>|z|     [95% Conf. Interval]
-------------+----------------------------------------------------------------
mu           |
       _cons |   58.86686   .3212832   183.22   0.000     58.23715    59.49656
-------------+----------------------------------------------------------------
sigma        |
       _cons |   11.50686   .2495473    46.11   0.000     11.01775    11.99596
-------------+----------------------------------------------------------------
shape        |
       _cons |  -10.73226   6.593758    -1.63   0.104    -23.65579    2.191267
------------------------------------------------------------------------------

Skewness parameter in CP
```

```
Coef.: -.96063923

Std. Err.: .90773842

[95% CI]: -2.7397739 , .81849539

.
. * CS weighted
. censn ageend [pw=weight2010] if female==0 & inse2010==1, lefttrun(agestart)
failure(
> died2011) difficult

Fitting constant-only model:

Iteration 0:   log pseudolikelihood = -185052.97  (not concave)
Iteration 1:   log pseudolikelihood = -112259.09  (not concave)
Iteration 2:   log pseudolikelihood = -99649.745  (not concave)
Iteration 3:   log pseudolikelihood = -95523.759  (not concave)
Iteration 4:   log pseudolikelihood = -94596.247  (not concave)
Iteration 5:   log pseudolikelihood = -94237.176
Iteration 6:   log pseudolikelihood = -94222.891
Iteration 7:   log pseudolikelihood = -94215.534
Iteration 8:   log pseudolikelihood = -94215.452
Iteration 9:   log pseudolikelihood = -94215.452

                                                Number of obs     =    120,916
                                                Wald chi2(0)      =          .
Log pseudolikelihood = -94215.452               Prob > chi2       =          .

------------------------------------------------------------------------------
             |               Robust
             |      Coef.   Std. Err.      z    P>|z|     [95% Conf. Interval]
-------------+----------------------------------------------------------------
mu           |
       _cons |   53.35408   .3200224   166.72   0.000     52.72685    53.98131
-------------+----------------------------------------------------------------
sigma        |
       _cons |   11.59281    .263112    44.06   0.000     11.07712     12.1085
-------------+----------------------------------------------------------------
shape        |
       _cons |  -4.431152   .4982506    -8.89   0.000    -5.407705   -3.454598
------------------------------------------------------------------------------

Skewness parameter in CP

Coef.: -.81751627

Std. Err.: .02368981

[95% CI]: -.86394745 , -.7710851

. censn ageend [pw=weight2010] if female==1 & inse2010==1, lefttrun(agestart)
failure(
> died2011) difficult

Fitting constant-only model:

Iteration 0:   log pseudolikelihood = -173915.31  (not concave)
Iteration 1:   log pseudolikelihood = -92448.893  (not concave)
Iteration 2:   log pseudolikelihood = -88863.195  (not concave)
Iteration 3:   log pseudolikelihood = -84096.035  (not concave)
Iteration 4:   log pseudolikelihood =  -82809.11  (not concave)
Iteration 5:   log pseudolikelihood = -80802.247  (not concave)
Iteration 6:   log pseudolikelihood = -77428.911
Iteration 7:   log pseudolikelihood = -76348.798
Iteration 8:   log pseudolikelihood = -76148.285
Iteration 9:   log pseudolikelihood = -76037.856  (not concave)
Iteration 10:  log pseudolikelihood = -75723.826  (not concave)
```

```
Iteration 11:  log pseudolikelihood = -74769.716
Iteration 12:  log pseudolikelihood = -74756.328
Iteration 13:  log pseudolikelihood = -74753.888
Iteration 14:  log pseudolikelihood = -74753.745
Iteration 15:  log pseudolikelihood = -74753.744

                                                Number of obs     =    131,852
                                                Wald chi2(0)      =          .
Log pseudolikelihood = -74753.744               Prob > chi2       =          .

------------------------------------------------------------------------------
             |               Robust
             |      Coef.   Std. Err.      z    P>|z|     [95% Conf. Interval]
-------------+----------------------------------------------------------------
mu           |
       _cons |   59.19869   .3833272   154.43   0.000     58.44738    59.94999
-------------+----------------------------------------------------------------
sigma        |
       _cons |   11.49346    .347541    33.07   0.000     10.81229    12.17463
-------------+----------------------------------------------------------------
shape        |
       _cons |   -8.59729   3.213214    -2.68   0.007    -14.89507   -2.299506
------------------------------------------------------------------------------

Skewness parameter in CP

Coef.: -.94216693

Std. Err.: .69825536

[95% CI]: -2.3107223 , .42638841

.
. * IP weighted
. censn ageend [pw=ipw20102011] if female==0 & inse2010==1, lefttrun(agestart)
failure
> (died2011) difficult

Fitting constant-only model:

Iteration 0:   log pseudolikelihood = -202229.14  (not concave)
Iteration 1:   log pseudolikelihood = -135485.16  (not concave)
Iteration 2:   log pseudolikelihood = -133481.83  (not concave)
Iteration 3:   log pseudolikelihood = -126737.97  (not concave)
Iteration 4:   log pseudolikelihood = -121380.72  (not concave)
Iteration 5:   log pseudolikelihood = -118450.75
Iteration 6:   log pseudolikelihood = -118307.33
Iteration 7:   log pseudolikelihood = -118297.77
Iteration 8:   log pseudolikelihood = -118297.74
Iteration 9:   log pseudolikelihood = -118297.74

                                                Number of obs     =    120,916
                                                Wald chi2(0)      =          .
Log pseudolikelihood = -118297.74               Prob > chi2       =          .

------------------------------------------------------------------------------
             |               Robust
             |      Coef.   Std. Err.      z    P>|z|     [95% Conf. Interval]
-------------+----------------------------------------------------------------
mu           |
       _cons |   50.92433   .3480457   146.32   0.000     50.24217    51.60648
-------------+----------------------------------------------------------------
sigma        |
       _cons |     11.721   .3029672    38.69   0.000      11.1272    12.31481
-------------+----------------------------------------------------------------
shape        |
       _cons |  -4.369367   .4449166    -9.82   0.000    -5.241388   -3.497347
------------------------------------------------------------------------------
```

```
Skewness parameter in CP

Coef.: -.8132418

Std. Err.: .01730091

[95% CI]: -.84715096 , -.77933264

. censn ageend [pw=ipw20102011] if female==1 & inse2010==1, lefttrun(agestart)
failure
> (died2011) difficult

Fitting constant-only model:

Iteration 0:   log pseudolikelihood = -204774.93  (not concave)
Iteration 1:   log pseudolikelihood = -141345.89  (not concave)
Iteration 2:   log pseudolikelihood = -132135.46  (not concave)
Iteration 3:   log pseudolikelihood =    -124297  (not concave)
Iteration 4:   log pseudolikelihood = -120640.55
Iteration 5:   log pseudolikelihood = -120188.26  (not concave)
Iteration 6:   log pseudolikelihood = -117491.46
Iteration 7:   log pseudolikelihood = -116210.43
Iteration 8:   log pseudolikelihood = -116144.22
Iteration 9:   log pseudolikelihood = -116132.02
Iteration 10:  log pseudolikelihood = -116131.94
Iteration 11:  log pseudolikelihood = -116131.94

                                                Number of obs     =    131,852
                                                Wald chi2(0)      =          .
Log pseudolikelihood = -116131.94               Prob > chi2       =          .

------------------------------------------------------------------------------
             |               Robust
             |      Coef.   Std. Err.      z    P>|z|     [95% Conf. Interval]
-------------+----------------------------------------------------------------
mu           |
       _cons |   54.80633    .348012   157.48   0.000     54.12424    55.48842
-------------+----------------------------------------------------------------
sigma        |
       _cons |    10.7091   .2875369    37.24   0.000     10.14553    11.27266
-------------+----------------------------------------------------------------
shape        |
       _cons |  -6.422058   .7590689    -8.46   0.000    -7.909806    -4.93431
------------------------------------------------------------------------------

Skewness parameter in CP

Coef.: -.90336406

Std. Err.: .07169454

[95% CI]: -1.0438828 , -.76284535

.
. replace agestart=agestart-35
(5,242,187 real changes made)

. replace ageend=ageend-35
(5,242,187 real changes made)

.
. * Restrict to individuals aged 65 years or older
. drop if agestart<0
(3,909,271 observations deleted)

.
. * STATPOP 2010
```

```
.
. censn ageend if female==0, lefttrun(agestart) failure(died2011)

Fitting constant-only model:

Iteration 0:   log likelihood = -102596.17  (not concave)
Iteration 1:   log likelihood = -86254.222
Iteration 2:   log likelihood = -84759.259
Iteration 3:   log likelihood = -83778.249
Iteration 4:   log likelihood = -83583.849
Iteration 5:   log likelihood = -83575.044
Iteration 6:   log likelihood = -83574.984
Iteration 7:   log likelihood = -83574.984

                                                Number of obs     =    572,936
                                                Wald chi2(0)      =          .
Log likelihood = -83574.984                     Prob > chi2       =          .

------------------------------------------------------------------------------
             |      Coef.   Std. Err.      z    P>|z|     [95% Conf. Interval]
-------------+----------------------------------------------------------------
mu           |
       _cons |   17.62691   .0853129   206.61   0.000      17.4597    17.79412
-------------+----------------------------------------------------------------
sigma        |
       _cons |   9.895403    .082609   119.79   0.000     9.733492    10.05731
-------------+----------------------------------------------------------------
shape        |
       _cons |  -3.329085   .0985781   -33.77   0.000    -3.522295   -3.135875
------------------------------------------------------------------------------

Skewness parameter in CP

Coef.: -.71360779

Std. Err.: .00020774

[95% CI]: -.71401496 , -.71320061

. censn ageend if female==1, lefttrun(agestart) failure(died2011)

Fitting constant-only model:

Iteration 0:   log likelihood = -122736.49  (not concave)
Iteration 1:   log likelihood = -100307.28  (not concave)
Iteration 2:   log likelihood = -97481.748
Iteration 3:   log likelihood = -97414.454
Iteration 4:   log likelihood = -97369.444
Iteration 5:   log likelihood = -97368.901
Iteration 6:   log likelihood = -97368.901

                                                Number of obs     =    759,980
                                                Wald chi2(0)      =          .
Log likelihood = -97368.901                     Prob > chi2       =          .

------------------------------------------------------------------------------
             |      Coef.   Std. Err.      z    P>|z|     [95% Conf. Interval]
-------------+----------------------------------------------------------------
mu           |
       _cons |   21.67194   .0562216   385.47   0.000     21.56174    21.78213
-------------+----------------------------------------------------------------
sigma        |
       _cons |   8.811544   .0511003   172.44   0.000     8.711389    8.911699
-------------+----------------------------------------------------------------
shape        |
       _cons |  -3.311655   .0745128   -44.44   0.000    -3.457697   -3.165612
------------------------------------------------------------------------------
```

```
Skewness parameter in CP

Coef.: -.71136644

Std. Err.: .00008992

[95% CI]: -.71154268 , -.71119019

.
. * SE 2010
.
. * Unweighted
. censn ageend if female==0 & inse2010==1, lefttrun(agestart) failure(died2011)
diffic
> ult

Fitting constant-only model:

Iteration 0:   log likelihood = -4700.6764  (not concave)
Iteration 1:   log likelihood = -3583.4826  (not concave)
Iteration 2:   log likelihood =  -3450.901
Iteration 3:   log likelihood =  -3408.459  (not concave)
Iteration 4:   log likelihood =  -3388.058  (not concave)
Iteration 5:   log likelihood = -3379.5622
Iteration 6:   log likelihood = -3379.1616
Iteration 7:   log likelihood = -3379.0658
Iteration 8:   log likelihood = -3379.0657

                                                Number of obs     =     27,886
                                                Wald chi2(0)      =          .
Log likelihood = -3379.0657                     Prob > chi2       =          .

------------------------------------------------------------------------------
             |      Coef.   Std. Err.      z    P>|z|     [95% Conf. Interval]
-------------+----------------------------------------------------------------
mu           |
       _cons |   19.31829   .3950729    48.90   0.000     18.54396    20.09262
-------------+----------------------------------------------------------------
sigma        |
       _cons |    10.3504   .3993859    25.92   0.000     9.567622    11.13319
-------------+----------------------------------------------------------------
shape        |
       _cons |  -3.988343   .6431322    -6.20   0.000    -5.248859   -2.727827
------------------------------------------------------------------------------

Skewness parameter in CP

Coef.: -.78341694

Std. Err.: .0470075

[95% CI]: -.87554995 , -.69128394

. censn ageend if female==1 & inse2010==1, lefttrun(agestart) failure(died2011)
diffic
> ult

Fitting constant-only model:

Iteration 0:   log likelihood =  -5242.606  (not concave)
Iteration 1:   log likelihood = -3223.1781  (not concave)
Iteration 2:   log likelihood = -3121.4439
Iteration 3:   log likelihood = -3095.9115
Iteration 4:   log likelihood = -3084.4262
Iteration 5:   log likelihood =  -3084.278
Iteration 6:   log likelihood = -3084.2743
Iteration 7:   log likelihood =  -3084.274
Iteration 8:   log likelihood = -3074.4087  (not concave)
```

```
Iteration 9:   log likelihood =  -3062.822
Iteration 10:  log likelihood = -3059.7895
Iteration 11:  log likelihood = -3059.3931
Iteration 12:  log likelihood = -3059.3643
Iteration 13:  log likelihood = -3059.3642

                                                Number of obs     =     36,579
                                                Wald chi2(0)      =          .
Log likelihood = -3059.3642                     Prob > chi2       =          .

------------------------------------------------------------------------------
             |      Coef.   Std. Err.      z    P>|z|     [95% Conf. Interval]
-------------+----------------------------------------------------------------
mu           |
       _cons |   25.17236   .2877932    87.47   0.000      24.6083    25.73643
-------------+----------------------------------------------------------------
sigma        |
       _cons |   9.048526   .2801547    32.30   0.000     8.499433    9.597619
-------------+----------------------------------------------------------------
shape        |
       _cons |  -4.696613    1.23662    -3.80   0.000    -7.120344   -2.272882
------------------------------------------------------------------------------

Skewness parameter in CP

Coef.: -.83435892

Std. Err.: .21247471

[95% CI]: -1.2508017 , -.41791613

.
. * CS weighted
. censn ageend [pw=weight2010] if female==0 & inse2010==1, lefttrun(agestart)
failure(
> died2011) difficult

Fitting constant-only model:

Iteration 0:   log pseudolikelihood = -93410.679  (not concave)
Iteration 1:   log pseudolikelihood = -68987.358  (not concave)
Iteration 2:   log pseudolikelihood = -67809.102
Iteration 3:   log pseudolikelihood = -67624.374
Iteration 4:   log pseudolikelihood = -67623.397
Iteration 5:   log pseudolikelihood = -67623.395

                                                Number of obs     =     27,886
                                                Wald chi2(0)      =          .
Log pseudolikelihood = -67623.395               Prob > chi2       =          .

------------------------------------------------------------------------------
             |               Robust
             |      Coef.   Std. Err.      z    P>|z|     [95% Conf. Interval]
-------------+----------------------------------------------------------------
mu           |
       _cons |   19.36009    .426696    45.37   0.000     18.52378     20.1964
-------------+----------------------------------------------------------------
sigma        |
       _cons |    10.2886   .4397643    23.40   0.000     9.426675    11.15052
-------------+----------------------------------------------------------------
shape        |
       _cons |   -3.34009   .4593412    -7.27   0.000    -4.240383   -2.439798
------------------------------------------------------------------------------

Skewness parameter in CP

Coef.: -.7150109
```

```
Std. Err.: .0189132

[95% CI]: -.7520801 , -.67794169

. censn ageend [pw=weight2010] if female==1 & inse2010==1, lefttrun(agestart)
failure(
> died2011) difficult

Fitting constant-only model:

Iteration 0:   log pseudolikelihood = -100050.83  (not concave)
Iteration 1:   log pseudolikelihood = -60606.273  (not concave)
Iteration 2:   log pseudolikelihood = -59276.844  (not concave)
Iteration 3:   log pseudolikelihood = -58196.748
Iteration 4:   log pseudolikelihood = -57602.433
Iteration 5:   log pseudolikelihood = -57564.789
Iteration 6:   log pseudolikelihood =   -57561.7
Iteration 7:   log pseudolikelihood = -57561.628
Iteration 8:   log pseudolikelihood = -57561.627

                                                Number of obs     =     36,579
                                                Wald chi2(0)      =          .
Log pseudolikelihood = -57561.627               Prob > chi2       =          .

------------------------------------------------------------------------------
             |               Robust
             |      Coef.   Std. Err.      z    P>|z|     [95% Conf. Interval]
-------------+----------------------------------------------------------------
mu           |
       _cons |   25.29731   .3267965    77.41   0.000      24.6568    25.93782
-------------+----------------------------------------------------------------
sigma        |
       _cons |   9.081732   .3346371    27.14   0.000     8.425855    9.737608
-------------+----------------------------------------------------------------
shape        |
       _cons |  -4.617616   .8146375    -5.67   0.000    -6.214276   -3.020955
------------------------------------------------------------------------------

Skewness parameter in CP

Coef.: -.82959126

Std. Err.: .08523924

[95% CI]: -.99665711 , -.66252542

.
. * IP weighted
. censn ageend [pw=ipw20102011] if female==0 & inse2010==1, lefttrun(agestart)
failure
> (died2011) difficult

Fitting constant-only model:

Iteration 0:   log pseudolikelihood = -103771.59  (not concave)
Iteration 1:   log pseudolikelihood = -85930.913  (not concave)
Iteration 2:   log pseudolikelihood = -84276.578  (not concave)
Iteration 3:   log pseudolikelihood = -83342.147
Iteration 4:   log pseudolikelihood = -83068.955
Iteration 5:   log pseudolikelihood = -83061.605
Iteration 6:   log pseudolikelihood = -83061.602

                                                Number of obs     =     27,886
                                                Wald chi2(0)      =          .
Log pseudolikelihood = -83061.602               Prob > chi2       =          .

------------------------------------------------------------------------------
             |               Robust
```

```
             |      Coef.   Std. Err.      z    P>|z|     [95% Conf. Interval]
-------------+----------------------------------------------------------------
mu           |
       _cons |   17.56986   .4635729    37.90   0.000     16.66127    18.47845
-------------+----------------------------------------------------------------
sigma        |
       _cons |   9.875256   .4529657    21.80   0.000      8.98746    10.76305
-------------+----------------------------------------------------------------
shape        |
       _cons |   -2.96837   .4330508    -6.85   0.000    -3.817134   -2.119606
------------------------------------------------------------------------------

Skewness parameter in CP

Coef.: -.66203515

Std. Err.: .01603815

[95% CI]: -.69346935 , -.63060096

. censn ageend [pw=ipw20102011] if female==1 & inse2010==1, lefttrun(agestart)
failure
> (died2011) difficult

Fitting constant-only model:

Iteration 0:   log pseudolikelihood = -126229.66  (not concave)
Iteration 1:   log pseudolikelihood = -99069.231
Iteration 2:   log pseudolikelihood = -95917.981  (not concave)
Iteration 3:   log pseudolikelihood = -94044.275
Iteration 4:   log pseudolikelihood = -93953.638
Iteration 5:   log pseudolikelihood =  -93950.73
Iteration 6:   log pseudolikelihood = -93950.729

                                                Number of obs     =     36,579
                                                Wald chi2(0)      =          .
Log pseudolikelihood = -93950.729               Prob > chi2       =          .

------------------------------------------------------------------------------
             |               Robust
             |      Coef.   Std. Err.      z    P>|z|     [95% Conf. Interval]
-------------+----------------------------------------------------------------
mu           |
       _cons |   21.55338   .3779895    57.02   0.000     20.81253    22.29423
-------------+----------------------------------------------------------------
sigma        |
       _cons |   8.777285   .3544104    24.77   0.000     8.082653    9.471916
-------------+----------------------------------------------------------------
shape        |
       _cons |   -4.35407   .6103511    -7.13   0.000    -5.550337   -3.157804
------------------------------------------------------------------------------

Skewness parameter in CP

Coef.: -.81216124

Std. Err.: .04097481

[95% CI]: -.89247039 , -.73185209

.
. log close
      name:  <unnamed>
       log:  F:\SNC\SurveyMortality\LE_orig_Nov2017.txt
  log type:  text
 closed on:   5 Nov 2017, 10:08:37
-------------------------------------------------------------------------------
------
```

Calibration adjustments to address bias in mortality analyses due to informative sampling - a census-linked survey analysis in Switzerland

André Moser

November 05, 2017

We used the estimates from censored skew-normal regression models (seperate Stata output) to produce the graph.

Figure 2

```
library(ggplot2)
plotdata <- read.csv("F:\\SNC\\SurveyMortality\\data\\LE30.csv")

plotdata$est <- as.numeric(as.character(plotdata$est))
plotdata$uci <- as.numeric(as.character(plotdata$uci))
plotdata$lci <- as.numeric(as.character(plotdata$lci))
plotdata$year <- 2011
plotdata$type <- factor(plotdata$type, levels=1:4,
                        labels=c("STATPOP 2010", "SE 2010 (unweighted)",
                                 "SE 2010 (IPW)", "SE 2010 (CSW)"))

plotdata$female <- factor(plotdata$female, levels=0:1, labels=c("Men", "Women"))

plotdata
```

```
##                   type female      est      lci      uci year
## 1         STATPOP 2010    Men 50.91063 50.79912 51.02215 2011
## 2         STATPOP 2010  Women 54.77967 54.67676 54.88259 2011
## 3 SE 2010 (unweighted)    Men 53.32098 52.76619 53.87578 2011
## 4 SE 2010 (unweighted)  Women 58.86686 58.23715 59.49656 2011
## 5        SE 2010 (IPW)    Men 50.92433 50.24217 51.60648 2011
## 6        SE 2010 (IPW)  Women 54.80633 54.12424 55.48842 2011
## 7        SE 2010 (CSW)    Men 53.35408 52.72685 53.98131 2011
## 8        SE 2010 (CSW)  Women 59.19869 58.44738 59.94999 2011
```

```
p <- ggplot(data=plotdata, aes(x=factor(year), y=est, color=type) )
p1 <- p + geom_point(position=position_dodge(width=0.2)) + geom_linerange(aes(ymax=uci, ymin=lci
p1
```

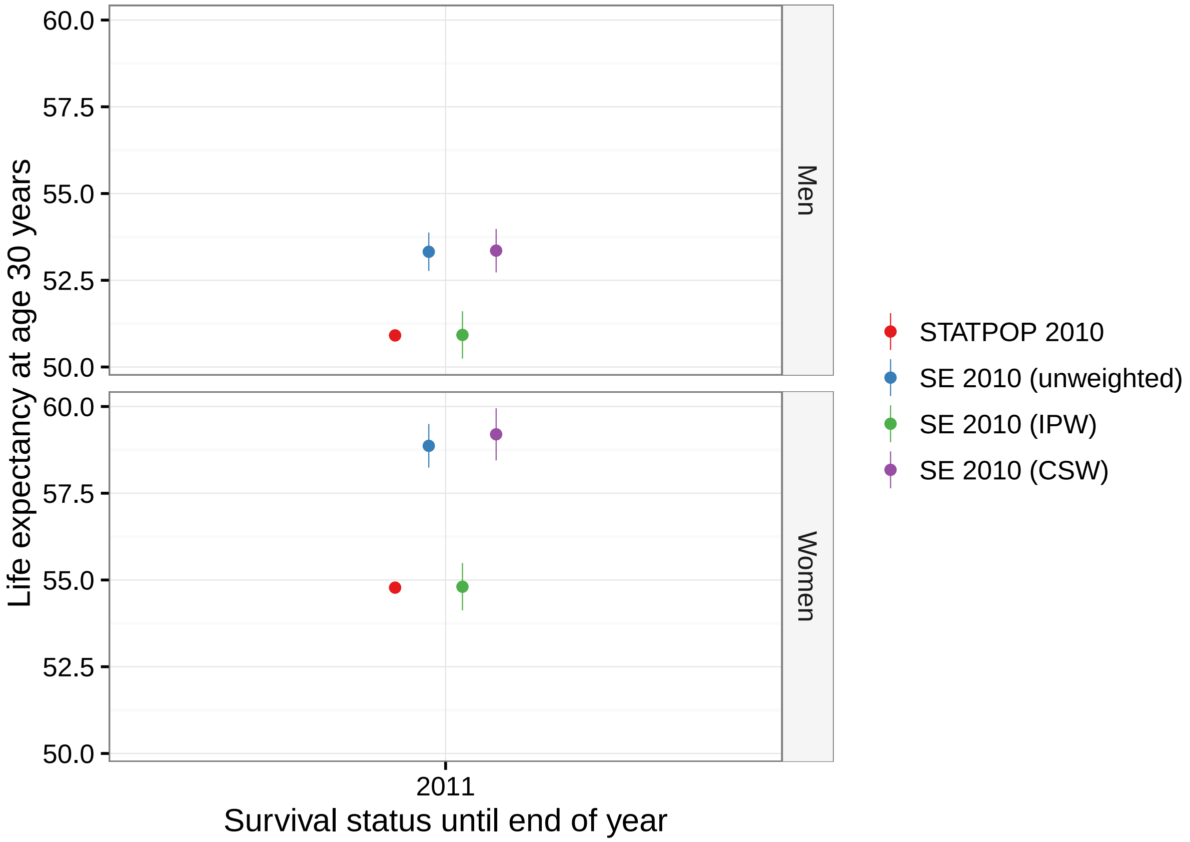


```
ggsave(file="F:\\SNC\\SurveyMortality\\LE30.tiff", plot=p1, width=19.05, height=22.23, dpi=600,
ggsave(file="F:\\SNC\\SurveyMortality\\LE30.jpeg", plot=p1, width=19.05, height=22.23, dpi=600,
```

Code for simulation (R code)

Used packages:

```
library(sn)

library(Hmisc)

library(survey)

library(foreign)
```

```
## Code for simulation study

library(sn)
library(Hmisc)
library(survey)
library(foreign)

set.seed(4527156)

n.sim <- 1000

### Define model for dying: Overall proportion of death given by qexp(x),
because exp parametric model assumed
hazards.died <- c(qexp(0.1))
est.badhealth.died <- log(4)

### Intercepts for sampling and nonresponse
intercepts.sampling <- c(qlogis(0.01), qlogis(0.1))
intercepts.nonresponse <- c(qlogis(0.1), qlogis(0.25))

outputwrite <- c()
outwrite <- c()
outwritefemale <- c()
outwriteage70 <- c()
outwriteage4070 <- c()

for (v in 1:length(hazards.died)) {

  hazard.died <- hazards.died[v]

for (j in 1:length(intercepts.sampling)) {

  intercept.sampling <- intercepts.sampling[j]

for (u in 1:length(intercepts.nonresponse)) {

  intercept.nonresponse <- intercepts.nonresponse[u]

    names.output <- Cs(unweighted, popweights, ipw1, ipw2)

list.output <- vector("list", length(names.output))
list.output.femalecoeff <- vector("list", length(names.output))
list.output.age4070coeff <- vector("list", length(names.output))
list.output.age70coeff <- vector("list", length(names.output))

list.output.se <- vector("list", length(names.output))
list.output.femalecoeff.se <- vector("list", length(names.output))
list.output.age4070coeff.se <- vector("list", length(names.output))
list.output.age70coeff.se <- vector("list", length(names.output))

names(list.output) <- names.output
names(list.output.femalecoeff) <- names.output
names(list.output.age4070coeff) <- names.output
names(list.output.age70coeff) <- names.output

names(list.output.se) <- paste(names.output, "_se", sep="")
names(list.output.femalecoeff.se) <- paste(names.output, "_se", sep="")
names(list.output.age4070coeff.se) <- paste(names.output, "_se", sep="")
names(list.output.age70coeff.se) <- paste(names.output, "_se", sep="")

list.output <- lapply(list.output, FUN=function(x) vector("double", n.sim))
list.output.femalecoeff <- lapply(list.output.femalecoeff, FUN=function(x)
vector("double", n.sim))
list.output.age4070coeff <- lapply(list.output.age4070coeff, FUN=function(x)
vector("double", n.sim))
list.output.age70coeff <- lapply(list.output.age70coeff, FUN=function(x)
vector("double", n.sim))
```

```
list.output.se <- lapply(list.output.se, FUN=function(x) vector("double",
n.sim))
list.output.femalecoeff.se <- lapply(list.output.femalecoeff.se, FUN=function(x)
vector("double", n.sim))
list.output.age4070coeff.se <- lapply(list.output.age4070coeff.se,
FUN=function(x) vector("double", n.sim))
list.output.age70coeff.se <- lapply(list.output.age70coeff.se, FUN=function(x)
vector("double", n.sim))

ratetotal <- vector("double", n.sim)
age4070coeff <- vector("double", n.sim)
age70coeff <- vector("double", n.sim)
femalecoeff <- vector("double", n.sim)

for (i in 1:n.sim) {

### Population size
n <- 1000000

############ Constructing population
############ Variables: age, female, u (categorical variable), died

### Age, centering and squaring
age <- rsn(n, dp=cp2dp(c(46.1, 12.7, 0.922), family="SN"))
age4070 <- ifelse(age>=40 & age<70, 1, 0)
age70 <- ifelse(age>=70, 1, 0)

### Women are assumed to be older; 51.2% women assumed
Lfemale = qlogis(0.51)
female <- rbinom(n, 1, plogis(Lfemale))

### Bad health status
Lbadhealth = qlogis(0.1) + log(3)*(age70) + log(0.7)*(female==1)
badhealth <- rbinom(n, 1, plogis(Lbadhealth))
#mod <- glm(badhealth~age4070+age70+female, family = binomial())
#exp(mod$coefficients)

#Linst <- qlogis(0.005)+log(5)*(Lbadhealth==1)
#inst <- rbinom(n, 1, plogis(Linst))

### Specify population model for log odds of "future" dying
# true event time: Exp => E(T|X)=1/[hazard*exp(X*beta)]
h <- hazard.died*exp(est.badhealth.died*(badhealth==1))
#h <- hazard.died

### Survival prop assumed to be equally distributed in [0,1]
deathtime <- -log(runif(n))/h

### Censoring after 1 year
died <- ifelse(deathtime <= rep(1,n), 1, 0)
survtime <- pmin(deathtime, rep(1,n))
#coxph(Surv(survtime, died)~female+age4070+age70+badhealth)
#coxph(Surv(survtime, died)~age4070+age70+badhealth, subset=female==0)
#coxph(Surv(survtime, died)~badhealth)
#coxph(Surv(survtime, died)~female+age4070+age70)

mod <- glm(died~age4070+age70+female, offset=log(survtime), family=poisson())
#mod <- glm(died~age70+age4070, offset=log(survtime), family=poisson())
ratetotal[i] <- mod$coeff[names(mod$coeff)%in%"(Intercept)"]
age4070coeff[i] <- mod$coeff[names(mod$coeff)%in%"age4070"]
age70coeff[i] <- mod$coeff[names(mod$coeff)%in%"age70"]
femalecoeff[i] <- mod$coeff[names(mod$coeff)%in%"female"]

############ End construction population

############ Constructing sampling

####### 1) Sampling with bias -> "Missing at random" (MAR)
```

```
LMAR1 <- intercept.sampling
mar.sample1 <- rbinom(n, 1, plogis(LMAR1))

# "Log odds of MAR non-responding" (independent of mar.sample1)
Lnonresponse.mar <- intercept.nonresponse + log(4)*(badhealth==1)
mar.sample2 <- mar.sample1
mar.sample2[mar.sample1==1] <- rbinom(sum(mar.sample1==1), 1,
(1-plogis(Lnonresponse.mar[mar.sample1==1])))

pop.mar.wghts <- rep(plogis(LMAR1), n)
pop.mar.wghts[mar.sample1==1] <-
pop.mar.wghts[mar.sample1==1]*(1-plogis(Lnonresponse.mar[mar.sample1==1]))
pop.mar.wghts <- 1/pop.mar.wghts

############ End construction sampling

############ Modelling

df <- data.frame(id=1:length(age), time=survtime, age70=age70, age4070=age4070,
female=female, badhealth=badhealth, died=died, marsample2=mar.sample2,
popmarwghts=pop.mar.wghts)

#### Inverse probability (IP) modelling

### IP model MAR: Using only u and proxy variable death (proxy for badhealth)
mod <- glm(marsample2~died, data=df, family=binomial())
df$ipw.sampling.mar.biased1 <- 1/predict(mod, type="response")

### IP model MAR: Using only u and proxy variable death, plus variables for
dying
mod <- glm(marsample2~female+age70+age4070, data=df, family=binomial())
df$ipw.sampling.mar.biased2 <- 1/predict(mod, type="response")

dfmarsample2 <- df[df$marsample2==1,]


#### Output modelling

####### Nonresponse is MAR

### Sampling with nonresponse, unweighted
mod <- glm(died~age70+age4070+female, offset=log(time), family=poisson(),
data=dfmarsample2)

list.output$unweighted[i] <- mod$coeff[names(mod$coeff)%in%"(Intercept)"]
list.output.age4070coeff$unweighted[i] <-
mod$coeff[names(mod$coeff)%in%"age4070"]
list.output.age70coeff$unweighted[i] <- mod$coeff[names(mod$coeff)%in%"age70"]
list.output.femalecoeff$unweighted[i] <- mod$coeff[names(mod$coeff)%in%"female"]

list.output.se$unweighted_se[i] <-
sqrt(vcov(mod)[dimnames(vcov(mod))[[1]]%in%"(Intercept)",
dimnames(vcov(mod))[[2]]%in%"(Intercept)"])
list.output.age4070coeff.se$unweighted_se[i] <-
sqrt(vcov(mod)[dimnames(vcov(mod))[[1]]%in%"age4070",
dimnames(vcov(mod))[[2]]%in%"age4070"])
list.output.age70coeff.se$unweighted_se[i] <-
sqrt(vcov(mod)[dimnames(vcov(mod))[[1]]%in%"age70",
dimnames(vcov(mod))[[2]]%in%"age70"])
list.output.femalecoeff.se$unweighted_se[i] <-
sqrt(vcov(mod)[dimnames(vcov(mod))[[1]]%in%"female",
dimnames(vcov(mod))[[2]]%in%"female"])

### Sampling with nonresponse, weighted with known calibrated survey weights
samdes <- svydesign(id=~id, weights=~popmarwghts, data=dfmarsample2)
mod <- svyglm(died~age70+age4070+female, offset=log(time), family=poisson(),
design=samdes)
```

```
list.output$popweights[i] <- mod$coeff[names(mod$coeff)%in%"(Intercept)"]
list.output.age4070coeff$popweights[i] <-
mod$coeff[names(mod$coeff)%in%"age4070"]
list.output.age70coeff$popweights[i] <- mod$coeff[names(mod$coeff)%in%"age70"]
list.output.femalecoeff$popweights[i] <- mod$coeff[names(mod$coeff)%in%"female"]

list.output.se$popweights_se[i] <-
sqrt(vcov(mod)[dimnames(vcov(mod))[[1]]%in%"(Intercept)",
dimnames(vcov(mod))[[2]]%in%"(Intercept)"])
list.output.age4070coeff.se$popweights_se[i] <-
sqrt(vcov(mod)[dimnames(vcov(mod))[[1]]%in%"age4070",
dimnames(vcov(mod))[[2]]%in%"age4070"])
list.output.age70coeff.se$popweights_se[i] <-
sqrt(vcov(mod)[dimnames(vcov(mod))[[1]]%in%"age70",
dimnames(vcov(mod))[[2]]%in%"age70"])
list.output.femalecoeff.se$popweights_se[i] <-
sqrt(vcov(mod)[dimnames(vcov(mod))[[1]]%in%"female",
dimnames(vcov(mod))[[2]]%in%"female"])


### Sampling with nonresponse, IP weighted
samdes <- svydesign(id=~id, weights=~ipw.sampling.mar.biased1,
data=dfmarsample2)
mod <- svyglm(died~age70+age4070+female, offset=log(time), family=poisson(),
design=samdes)

list.output$ipw1[i] <- mod$coeff[names(mod$coeff)%in%"(Intercept)"]
list.output.age4070coeff$ipw1[i] <- mod$coeff[names(mod$coeff)%in%"age4070"]
list.output.age70coeff$ipw1[i] <- mod$coeff[names(mod$coeff)%in%"age70"]
list.output.femalecoeff$ipw1[i] <- mod$coeff[names(mod$coeff)%in%"female"]

list.output.se$ipw1_se[i] <-
sqrt(vcov(mod)[dimnames(vcov(mod))[[1]]%in%"(Intercept)",
dimnames(vcov(mod))[[2]]%in%"(Intercept)"])
list.output.age4070coeff.se$ipw1_se[i] <-
sqrt(vcov(mod)[dimnames(vcov(mod))[[1]]%in%"age4070",
dimnames(vcov(mod))[[2]]%in%"age4070"])
list.output.age70coeff.se$ipw1_se[i] <-
sqrt(vcov(mod)[dimnames(vcov(mod))[[1]]%in%"age70",
dimnames(vcov(mod))[[2]]%in%"age70"])
list.output.femalecoeff.se$ipw1_se[i] <-
sqrt(vcov(mod)[dimnames(vcov(mod))[[1]]%in%"female",
dimnames(vcov(mod))[[2]]%in%"female"])

### Sampling with nonresponse, IP weighted
samdes <- svydesign(id=~id, weights=~ipw.sampling.mar.biased2,
data=dfmarsample2)
mod <- svyglm(died~age70+age4070+female, offset=log(time), family=poisson(),
design=samdes)

list.output$ipw2[i] <- mod$coeff[names(mod$coeff)%in%"(Intercept)"]
list.output.age4070coeff$ipw2[i] <- mod$coeff[names(mod$coeff)%in%"age4070"]
list.output.age70coeff$ipw2[i] <- mod$coeff[names(mod$coeff)%in%"age70"]
list.output.femalecoeff$ipw2[i] <- mod$coeff[names(mod$coeff)%in%"female"]

list.output.se$ipw2_se[i] <-
sqrt(vcov(mod)[dimnames(vcov(mod))[[1]]%in%"(Intercept)",
dimnames(vcov(mod))[[2]]%in%"(Intercept)"])
list.output.age4070coeff.se$ipw2_se[i] <-
sqrt(vcov(mod)[dimnames(vcov(mod))[[1]]%in%"age4070",
dimnames(vcov(mod))[[2]]%in%"age4070"])
list.output.age70coeff.se$ipw2_se[i] <-
sqrt(vcov(mod)[dimnames(vcov(mod))[[1]]%in%"age70",
dimnames(vcov(mod))[[2]]%in%"age70"])
list.output.femalecoeff.se$ipw2_se[i] <-
sqrt(vcov(mod)[dimnames(vcov(mod))[[1]]%in%"female",
dimnames(vcov(mod))[[2]]%in%"female"])
```

```
}

outwrite <- rbind(outwrite, data.frame(covariate="rate",
deathprop=pexp(hazard.died), sampprop=plogis(intercept.sampling),
nrprop=plogis(intercept.nonresponse), popval=ratetotal,
as.data.frame(list.output), as.data.frame(list.output.se)))

outwritefemale <- rbind(outwritefemale, data.frame(covariate="female",
deathprop=pexp(hazard.died), sampprop=plogis(intercept.sampling),
nrprop=plogis(intercept.nonresponse), popval=femalecoeff,
as.data.frame(list.output.femalecoeff),
as.data.frame(list.output.femalecoeff.se)))

outwriteage70 <- rbind(outwriteage70, data.frame(covariate="age70",
deathprop=pexp(hazard.died), sampprop=plogis(intercept.sampling),
nrprop=plogis(intercept.nonresponse), popval=age70coeff,
as.data.frame(list.output.age70coeff),
as.data.frame(list.output.age70coeff.se)))

outwriteage4070 <- rbind(outwriteage4070, data.frame(covariate="age4070",
deathprop=pexp(hazard.died), sampprop=plogis(intercept.sampling),
nrprop=plogis(intercept.nonresponse), popval=age4070coeff,
as.data.frame(list.output.age4070coeff),
as.data.frame(list.output.age4070coeff.se)))

}
}
}

outputwrite <- rbind(outputwrite, outwrite, outwritefemale, outwriteage70,
outwriteage4070 )
outputwrite
```

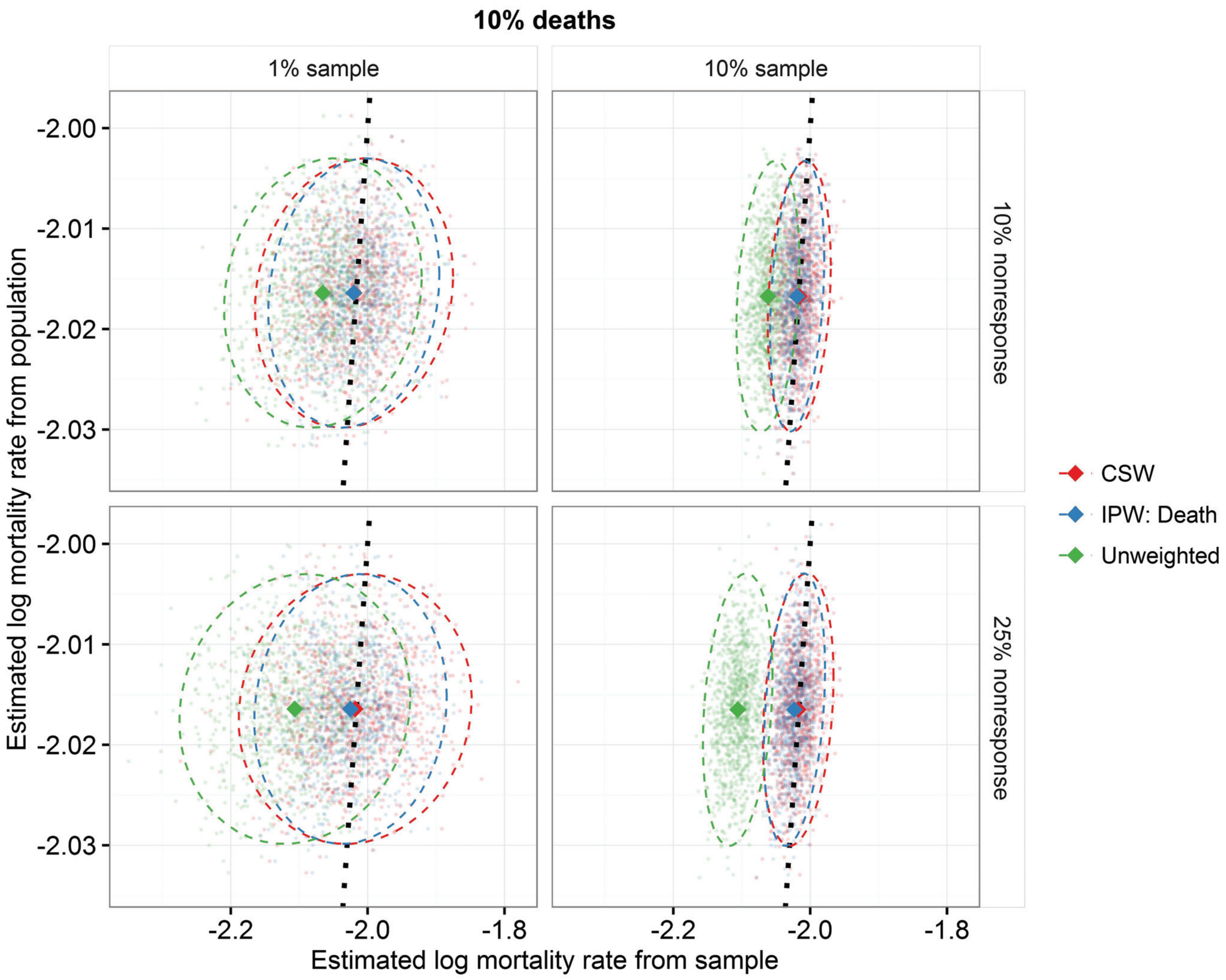
10% deaths
1% sample
10% sample
10% nonresponse
25% nonresponse
-2.00
-2.01
-2.02
-2.03
-2.2
-2.0
-1.8
Estimated log mortality rate from population
Estimated log mortality rate from sample
CSW
IPW: Death
Unweighted

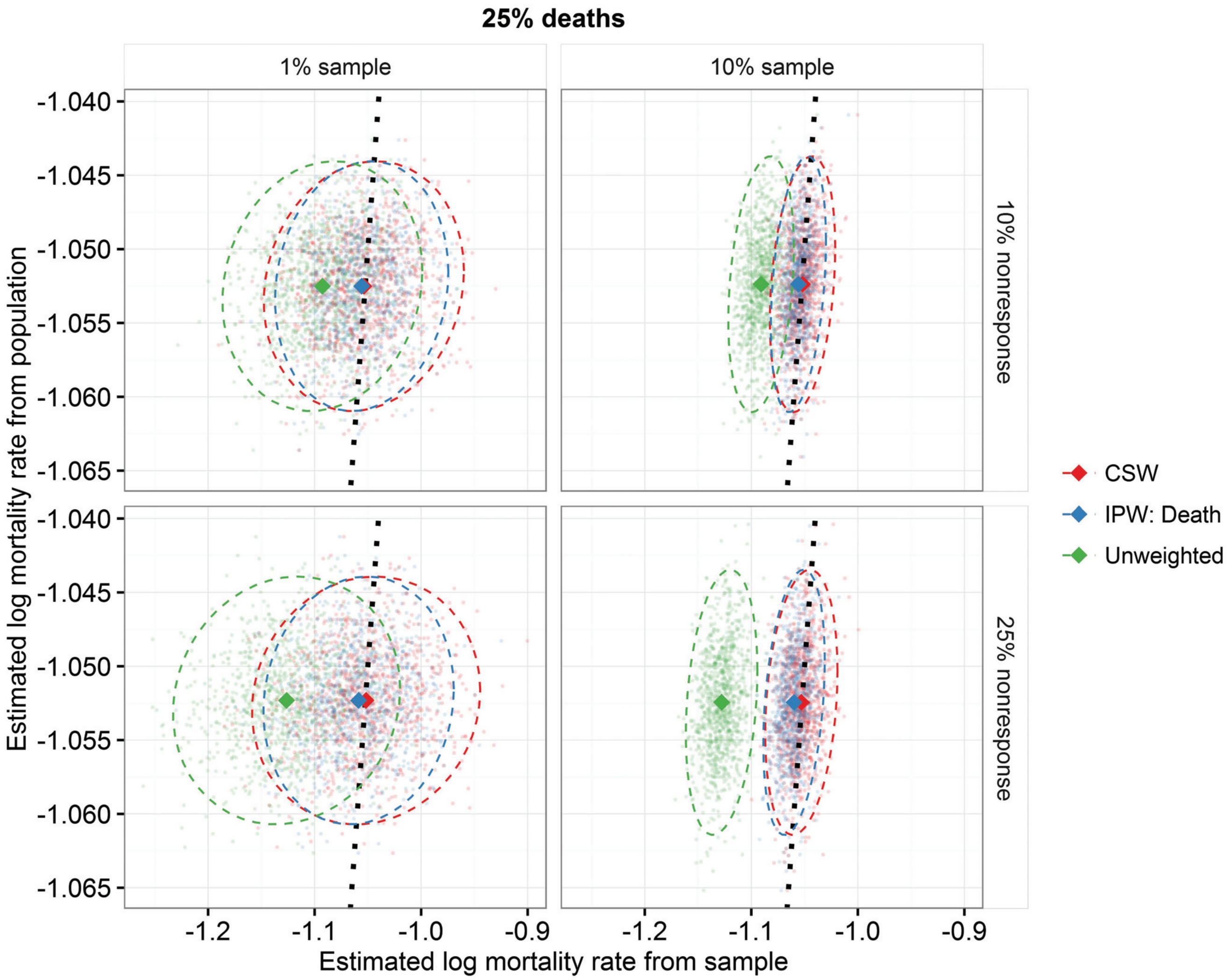
25% deaths
1% sample
10% sample
10% nonresponse
25% nonresponse
-1.040
-1.045
-1.050
-1.055
-1.060
-1.065
-1.2
-1.1
-1.0
-0.9
Estimated log mortality rate from population
Estimated log mortality rate from sample
CSW
IPW: Death
Unweighted

Construction of inverse probability weights (Stata code)

```
use "F:\SNC\SurveyMortality\data\SwissCensuses.dta", clear

*** Weight construction for STATPOP/SE 2010, only

stset statpop2010_enddate2011 if statpop2010==1, origin(statpop2010_startdate)
failure(died2011) scale(365.25)
sts gen na2011=na if statpop2010==1
stset statpop2010_enddate2012 if statpop2010==1, origin(statpop2010_startdate)
failure(died2012) scale(365.25)
sts gen na2012=na if statpop2010==1
stset statpop2010_enddate2013 if statpop2010==1, origin(statpop2010_startdate)
failure(died2013) scale(365.25)
sts gen na2013=na if statpop2010==1
stset statpop2010_enddate2014 if statpop2010==1, origin(statpop2010_startdate)
failure(died2014) scale(365.25)
sts gen na2014=na if statpop2010==1

replace na2011=na2011*10000
replace na2012=na2012*10000
replace na2013=na2013*10000
replace na2014=na2014*10000

drop _st _d _t _t0

logistic inse2010 female##r10_statpop_agecat5##i.died2011 na2011 i.r10_canton_se
i.r10_nat i.r10_civil
predict prob1

logistic inse2010 female##r10_statpop_agecat5##i.died2012 na2012 i.r10_canton_se
i.r10_nat i.r10_civil
predict prob2

logistic inse2010 female##r10_statpop_agecat5##i.died2013 na2013 i.r10_canton_se
i.r10_nat i.r10_civil
predict prob3

logistic inse2010 female##r10_statpop_agecat5##i.died2014 na2014 i.r10_canton_se
i.r10_nat i.r10_civil
predict prob4

gen ipw20102011=1/prob1
gen ipw20102012=1/prob2
gen ipw20102013=1/prob3
gen ipw20102014=1/prob4

drop prob1 prob2 prob3 prob4
```
